# Supplementary material for: Factors influencing active tuberculosis case-finding policy development and implementation: a scoping review
Source: BMJ Open. 2019 Dec 11;9(12):e031284. doi: 10.1136/bmjopen-2019-031284 (PMC6924749; doi:10.1136/bmjopen-2019-031284)
Supplement: Supplementary data [file bmjopen-2019-031284supp002.pdf]

Additional file 2: Literature search strategy for MEDLINE

We implemented the search strategy on 24 January 2018. The search yielded 4640 results.

1 exp Tuberculosis/ or exp Mycobacterium tuberculosis/

2 (tuberculosis or tb or pulmonary consumption or consumption, pulmonary or pulmonary phthisis or tuberculoses).ti,ab.

3 1 or 2

4 exp Mass screening/ or exp Mass chest x-ray/ or exp Contact tracing/ or exp Health surveys/ or exp Cross-sectional studies/ or exp Epidemiologic studies/ or exp Systematic review/

5 (Mass chest x ray$ or screen$ or mass screening$ or population screening$ or survey$ or screening survey$ or household survey or health survey$ or cross-sectional or detect$ or active case or case detect$ or case finding or tuberculosis case finding or active case finding or contact tracing or contact examination or contact screening or contact investigation or employment testing or intensified case finding or prevalence stud$ or prevalen* or inciden* or algorithm or undiagnosed or checking or pre-entry or passive or TB suspect$ or notification or notified).ti,ab.

6 4 or 5

7 (view$ or barrier$ or block$ or obstacle$ or hinder$ or constrain$ or facilitat$ or enabl$ or benefit$ or opportun$ or attitude$ or opinion$ or belief$ or perceiv$ or perception$ or aware$ or personal view$ or motivat$ or reason$ or incentiv$ or cost$ or cost-effective$ or cost analys$ or cost comparison or cost-minimization or cost measure$ or affordab$ or resource$ or resource allocation or resource$ or efficiency).ti,ab.

Biermann O, *et al. BMJ Open* 2019; 9:e031284. doi: 10.1136/bmjopen-2019-031284

8 exp Attitude/ or exp Motivation/ or exp Cost/ or exp Cost analysis/ or exp Resource allocation/

9 7 or 8

10 exp Policy Making/ or exp Health Plan Implementation/ or exp Health Priorities/

11 (policy mak$ or policy develop$ or policy analys$ or health polic$ or policy implement$ or health plan implement$ or priorit$ or health priorit$).ti,ab.

12 10 or 11

13 9 or 12

14 (((((((((((((((((hard$ adj2 reach) or hard$) adj2 locate) or hard$) adj2 find) or hard$) adj2 treat) or difficult) adj2 locate) or Difficult) adj2 engage) or social$ exclu$ or social inequalit$ or difficult$) adj2 reach) or difficult$) adj2 find) or difficult$) adj2 treat).ti,ab.

15 ((geograph$ or transport$ or physical) and barrier$).ti,ab.

16 ((low$ or poor$ or negative) and (quality adj2 life)).ti,ab.

17 ((vulnerable or disadvantaged or at risk or high risk or low socioeconomic status or neglect$ or marginal$ or forgotten or non-associative or unengaged or hidden or excluded or transient or inaccessible or underserved or stigma$ or inequitable) and (people or population$ or communit$ or neighbourhood$1 or neighborhood$1 or group$ or area$1 or demograph$ or patient$ or social$)).ti,ab.

18 vulnerable populations/

19 17 or 18

20 poverty area/

21 (refuser$1 or non-user$1 or discriminat$ or shame or prejud$ or racism or racial discriminat$).ti,ab.

22 social support/ or *social conditions/ or stigma/ or Social Isolation/ or *quality of life/ or Prejudice/ or Socioeconomic Factors/

23 (immobile or (disabled and (house bound or home bound)) or ((house or home) and bound)).ti,ab.

24 Homebound Persons/

25 23 or 24

26 ((hous$ and (quality or damp$ or standard$ or afford$ or condition$ or dilapidat$)) or ((emergency or temporary or inadequate or poor$ or overcrowd$ or over-crowd$ or over-subscribed) and (hous$ or accommodation or shelter$ or hostel$ or dwelling$))).ti,ab.

27 housing/st

28 26 or 27

29 (rough sleep$ or runaway$1 or ((homeless$ or street or Destitut$) and (population or person$1 or people or group$ or individual$1 or shelter$ or hostel$ or accommodation$1))).ti,ab.

30 exp homeless persons/

31 29 or 30

32 ((drug$ or substance) and (illegal or misus$ or abuse or intravenous or IV or problem use$ or illicit use$ or addict$ or dependen$ or dependant or delinquency)).ti,ab.

33 *Substance-Related Disorders/ or Drug users/

34 32 or 33

35 ((alcohol$ and (misus$ or abuse or problem$ use$ or problem drink$ or illicit use$ or addict$ or dependen$ or dependant or delinquency)) or alcoholic$1).ti,ab.

36 *Alcohol- Related Disorders/ or Alcoholics/

37 35 or 36

38 (prostitution or sex work$ or transactional sex$ or prostitute$1).ti,ab.

39 Prostitution/

40 38 or 39

41 (poverty or deprivation or financial hardship$).ti,ab.

42 ((low-income or low income or low pay or low paid or poor or deprived or debt$ or arrear$) and (people or person$1 or population$1 or communit$ or group$ or social group$ or neighbourhood$1 or neighborhood$1 or famil$)).ti,ab.

43 poverty/

44 (low$ and social class$).ti,ab.

45 (traveller$1 or gypsies or gypsy or Romany or roma).ti,ab.

46 gypsies/

47 45 or 46

48 (mental$ and (health or ill or illness)).ti,ab.

Biermann O, *et al. BMJ Open* 2019; 9:e031284. doi: 10.1136/bmjopen-2019-031284

49 *mental health/ or Mentally Ill Persons/

50 48 or 49

51 ((((health care worker$1 or health care) adj2 service provi$) or health-care) adj2 provi$).ti,ab.

52 (outreach adj2 worker$1).ti,ab.

53 Community health aides/

54 52 or 53

55 (support adj2 worker$1).ti,ab.

56 (case adj2 worker$1).ti,ab.

57 (social adj2 worker$1).ti,ab.

58 social care professional$1.ti,ab.

59 ((social care adj2 service provi$) or (social-care adj2 provi$)).ti,ab.

60 (((language$ or communicat$) and (barrier$ or understand$ or strateg$ or proficien$)) or translat$ or interpret$ or (cultur$ and competen$)).ti,ab.

61 Communication Barriers/ or *Language/

62 60 or 61

63 (((immigrant$ or migrant$ or asylum or refugee$ or undocumented or foreign born or born) adj overseas) or (displaced and (people or person$1))).ti,ab.

Biermann O, *et al. BMJ Open* 2019; 9:e031284. doi: 10.1136/bmjopen-2019-031284

64 ((((Emigration.mp. and Immigration/) or refugees/ or Transients.mp.) and migrants/) or Emigrants.mp.) and immigrants/ [mp=title, abstract, original title, name of substance word, subject heading word, keyword heading word, protocol supplementary concept word, rare disease supplementary concept word, unique identifier, synonyms]

65 63 or 64

66 exp Diabetes Mellitus/

67 diabet$.tw,ot.

68 (IDDM or NIDDM or MODY or T1DM or T2DM or T1D or T2D).tw,ot.

69 (non insulin$ depend$ or noninsulin$ depend$ or non insulin?depend$ or noninsulin?depend$).tw,ot.

70 (insulin$ depend$ or insulin?depend$).tw,ot.

71 or/66-70

72 14 or 15 or 16 or 19 or 20 or 21 or 22 or 25 or 28 or 31 or 34 or 37 or 40 or 41 or 42 or 43 or 44 or 47 or 50 or 51 or 54 or 55 or 56 or 57 or 58 or 59 or 62 or 65 or 71

73 3 and 6 and 13 and 72

74 limit 73 to yr="1968 -Current"

75 animals/ not humans/

76 74 not 75

Biermann O, *et al*. *BMJ Open* 2019; 9:e031284. doi: 10.1136/bmjopen-2019-031284
