# Supplementary material for: Factors influencing active tuberculosis case-finding policy development and implementation: a scoping review
Source: BMJ Open. 2019 Dec 11;9(12):e031284. doi: 10.1136/bmjopen-2019-031284 (PMC6924749; doi:10.1136/bmjopen-2019-031284)
Supplement: Supplementary data [file bmjopen-2019-031284supp003.pdf]

Additional file 3: Abridged version of the data charting table

The abridged data charting table contains information on author, year of publication and study design (column 1), the country where the study was implemented and the target population (column 2), ACF antecedents (column 3), ACF components (column 4), factors influencing ACF policy implementation at the level of the health system (column 5) and the individual and community level (column 6).

	1	2	3	4	5	6
**ID**	**Author, year, design**	**Country, target population**	**ACF antecedents**	**ACF components**	**Factors influencing ACF policy implementation:** health system context	**Factors influencing ACF policy implementation:** individual and community context
1	Abubakar, 2012, review	Low-burden, migrants			Screening site characteristics; diagnostic test availability; molecular assay costs	
2	Adejumo, 2016, qualitative	Nigeria, community			CHW age, work experience, knowledge	
3	Akkerman, 2016, letter to editor	the Netherlands, asylum-seekers			High-volume CXR readers; cooperation between public health and clinical workers	

Biermann O, *et al. BMJ Open* 2019; 9:e031284. doi: 10.1136/bmjopen-2019-031284

4	Alvarez*, 2011, descriptive	Low-burden, migrants	despite lack of evidence, ACF is major global health priority			
5	Ayakaka, 2017, qualitative	Uganda, contacts		Education and incentivization for health workers; persuasion; enablement	CHW travel costs, pay, skills; personalized, enabling services; staff shortages; fear of TB; patient communication; language barrier; difficulty locating households; lack of ACF guidelines	Fear of stigma; discrimination; among contacts: limited knowledge, mistrust, travel costs, avoidant behavior; privacy provided by home evaluation
6	Ayles, 2013, quantitative	South Africa, Zambia, contact			Linkage and retention in care	Empowerment to seek care early
7	Azman*, 2014, quantitative	China, India, South Africa, community				
8	Baxter, 2017, review	Worldwide, contacts			Using molecular epidemiological testing; ensuring adequate systems; local expertise and capability; communication; coordination; prompt action; addressing fear; error; lack of resources	Stigma; language barrier; high mobility

9	Bell*, 2013, quantitative	USA, migrants				
10	Bloss, 2011, case study	USA, outbreak			Institutional knowledge about TB control; limited human resources	Community trust and rapport
11	Van den Bosch, 2000, descriptive	UK, migrants		Incentivization for screening participants; health education for general public		Change of address; false address; language barrier; mistrust of authorities; fear of TB and stigma
12	Brewin, 2006, qualitative	UK, migrants			Integration of health and social services; waiting time	Feelings of reassurance, responsibility, anxiety linked to ACF; lack of public health information
13	Chemtob, 2003, qualitative	Israel, migrants			Adequate health facilities; funds for transportation; attitudes; dialogue with immigrants and providers in planning and implementation	Socio-cultural barriers; anti-immigrant sentiments; fear of deportation; no standard term for TB; empowerment to assume greater responsibility for health promotion

14	Cook, 2012, descriptive	Canada, aboriginal community			Foundation of routine ACF including interview skills, dedicated resources to undertake ACF, follow-up	Community buy-in
15	Corbett, 2013, review	Africa, PLHIV	Radiological screening widely implemented before 1970s	Target group: populations with high prevalence	Sensitivity of diagnostic tests; communication and interaction with the community; transportation	
16	Corbett, 2010, quantitative	Zimbabwe, congregate setting	ACF implemented in HIC since 1920s; ACF discouraged due to costs and weak treatment programmes			
17	Datiko, 2017, quantitative	Ethiopia, community		Linkage to laboratory services and treatment through a transport network		
18	de Vries, 2017, review	Worldwide, high-risk		Awareness; cultural sensitivity; access to high-quality	Structural barriers; cultural and language barriers	Low perceived susceptibility; stigma and its social consequences

				translation services; cross-cultural training of providers		
19	Dierberg, 2016, quantitative	India, refugees			Use of Xpert MTB/RIF; available time and human resources; lack of a laboratory technician; trained providers	High mobility; awareness; stigma
20	Eang, 2012, quantitative	Cambodia, contacts	Growing interest in ACF in LMIC			
21	Erkens*, 2008, quantitative	the Netherlands, migrants				
22	Mhimbira, 2017, review	Worldwide, contacts		Health promotion activities; education for health workers		
23	Fox, 2018, quantitative	Vietnam, contacts	ACF in high-prevalence settings remains limited			

Biermann O, *et al. BMJ Open* 2019; 9:e031284. doi: 10.1136/bmjopen-2019-031284

24	Fox, 2013, review	Worldwide, contacts, congregate setting	Growing interest ACF in LMICs			
25	Fox, 2015, quantitative	Vietnam, contacts	WHO recommendations for ACF		Proximity of services	Discrimination; lack of knowledge; misconceptions; attitudes and (traditional) beliefs; difficulty remembering appointments
26	Getnet, 2017, quantitative	Ethiopia, pastoralists			Proximity of services; diagnostic algorithm and tools used; quality of diagnostic services; provider expertise; burden on CHWs; health education for TB patients	Health seeking behavior; lack of confidence in CHWs; personal or cultural beliefs; gender norms
27	Godfrey-Faussett, 2003, review	Worldwide, high HIV prevalence setting	Movement towards decentralized diagnostic centers		Fear; stigma	
28	Golub, 2005, review	Worldwide, contacts	Mass radiography as ACF strategy in HICs before 1960s; WHO		Workload and cost; method of case detection; training of laboratory techni-	

			recommendation against indiscriminate mass screening (1974); exploration of new ACF strategies; recognized global urgency		cians, clinicians and field staff; appropriate bacteriological examination; laboratory network	
29	O'Hara, 2015, quantitative	South Africa, health care workers			Cost and confidentiality of services; waiting times	Sex, occupation, time since a previous TB test
30	Harper, 1996, quantitative	Nepal, remote community			Cost; lack of staff and funding; absenteeism; use of 'alternative' services; accessibility of services	Low confidence amongst users and general under-usage of services
31	Hinderaker, 2011, quantitative	Low-income, low-income community	Testing of different ACF approaches since 1960s, WHO targets set 2005	Social mobilization; health education for general public; communication; private sector engagement;	Regular supervision; availability of district laboratory supervisor	

				health systems strengthening; incentivization for health workers and TB patients		
32	Hogan, 2005, quantitative	UK, migrants			Service capacity; lack of national guidance; inconsistency in implementing ACF	
33	Honarvar, 2014, quantitative	Iran, migrants				Stigma; high mobility; poverty; low literacy; tribal behaviors; traditional beliefs; gender norms; language barrier; self-discrimination
34	Karki, 2017, quantitative	Papua New Guinea, community	Role of ACF uncertain since 1970s; WHO recommendation against indiscriminate mass screening (1974)		Poor health services; lack of outreach services; limited funds at district level; cost; availability of staff and the payment of allowances; staff skills and experience; health education for general public	

35	Kerrigan, 2017, qualitative	South Africa, contacts	Despite lack of evidence, ACF is major global health priority		Provider training, safety; incentivization for screening participants; lack of resources, logistics; involvement of culturally respected, valued stakeholder/organization venues; community-based mobilization and events	Stigma; preference of different screening locations; travel time and costs; gossip
36	Khan*, 2015, quantitative	Canada, migrants				
37	Klinkenberg, 2009, review	EU, migrants			Access to healthcare for migrants; availability of a good follow-up system; integration with healthcare	
38	Kranzer, 2010, review	Worldwide, PLHIV	Mass radiography in Czechoslovakia 1961-72 showing decrease in prevalence	Target population; screening strategy; laboratory capacity; operational feasibility; costs	Feasibility and costs; laboratory capacity	

Manuscript for submission to *BMJ Open* Olivia Biermann et al.

39	Kranzer, 2012, review	South Africa, deprived community		Incentivization for screening participants		Waiting time; ability to produce a sputum sample; health status
40	Kulane, 2010, qualitative	Sweden, Somali community				Stigma; migration; migration policies; fear of deportation; misconceptions
41	Layton, 1995, review	USA, homeless			Incentivization for screening participants	
42	Li, 2017, case study	China, community			Heavy workload; inadequate providers; time and human resource constraints; provider education and competence	Acceptance; knowledge; awareness; health status; incentivization for screening participants; health education for TB patients
43	Lorent, 2015, mixed methods	Cambodia, urban poor		Health education for general public; community involvement; peer support networks	Challenging working conditions; collaboration between TB workers and CHWs; CHW compensation, familiarity, knowledge of community, remuneration; provider motivation; financial support	Acceptance; cost, fear; embarrassment; preference of different screening locations; privacy concerns; involvement of CHWs; non-acceptance of diagnosis

Biermann O, *et al. BMJ Open* 2019; 9:e031284. doi: 10.1136/bmjopen-2019-031284

44	Lorent, 2014, quantitative	Cambodia, urban poor		Flexible patient-centered treatment approach	Logistics; finances; heavy workload; CHW integration, network, recognition, remuneration; use of mobile phone, collaboration with providers and laboratory technicians; infrastructure	
45	Lönnroth, 2013, review	Worldwide, high-risk	WHO recommendation against indiscriminate mass screening (1974)	Confidentiality; access to free, good quality services; provider training; minimization of initial loss to follow-up; enforced notification; engagement of public/private providers; resources	Available, high-quality services; NTP performance; health system capacity; public health law; existing platforms for outreach and health promotion activities, health and social services	.;

46	Lönnroth, 2017, review	Low incidence, migrants		Target groups; screening algorithms; timing; values; preferences; competing health care needs; weak evidence base		
47	Lönnroth, 2015, descriptive	Low-incidence, high-risk				Trust
48	MacPherson, 2006, review	Low-incidence, migrants				Fear; language barrier; culture; social factors
49	Mallick*, 2017, descriptive	India, prisoners				
50	Mayo, 1996, case study	USA, homeless			ACF location; incentivization for TB patients; providers' frustration; involvement of clients	Stigma; substance use; poor social skills; health status; poor access to services and the necessities of life; unemployment; homelessness

51	Mor, 2008, quantitative	Israel, migrants		Unification of medical procedures	Operational cost; infrastructure	
52	Morishita, 2016, quantitative	Cambodia, contacts				Cost; insurance
53	Mulder, 2011, descriptive	the Netherlands, contacts			Limited health system capacity to keep track of immigrant index cases	
54	Mwansa-Kambafwile, 2013, quantitative	South Africa, contacts				Discomfort linked to home visits; confidentiality; stigma; discrimination
55	Ospina, 2012, quantitative	Spain, migrants		Involvement of CHWs	CHW involvement; language barrier	Birthplace; unknown district of residence; HIV infection; homelessness; sex; incarceration history; language and cultural barrier
56	Seedat, 2014, qualitative	UK, migrants		Community, proactive services; collaboration with	Lack of capacity, funding, advocacy, psycho-social support services, aware-	Stigma; culture; gender norms; faith; language barrier; misconceptions; fear of lack of confidentiality, cost,

				community organizations; combining diseases into health check-up; awareness raising	ness; link with community organizations; cultural insensitivity; time and distance to services	disease status; lack of awareness, knowledge, confidence
57	Sekandi, 2015, quantitative	Uganda, contacts	ACF currently used in research settings		Lack of well-organized public health systems; staff shortage; limited resources for personnel time, administration and transportation	
58	Shrestha-Kuwahara, 2003, qualitative	USA, contacts			Lack of clear definitions; challenge of communicating the need to obtain personal information; limited knowledge on socio-economic issues among staff; training of, frustration among providers	Trust; understanding; knowledge; feeling moral obligations to family/friends confusion; fear of alienation, abandonment, stigma, loss of employment or housing; negative perception of interview process; misconceptions; language and cultural barriers

59	Shrivastava, 2013, quantitative	India, slums	Periodic ACF remains an integral part of TB control in high-risk groups	Involvement of CHW		
60	Smit*, 2017, quantitative	Belgium, high-risk, contacts				
61	Southern, 1999, observational	UK, homeless		Immediate examination of CXR; fast-track referral system	Non-judgmental, flexible, supportive approach	Lack of interest; fear of the outcome; waiting times; health education of screening participants
62	Suthar, 2016, perspective	Worldwide, high-risk	2030 goals important for inspiring and persuading communities to collaborate and step up TB response			
63	Swigart, 2004, qualitative	USA, homeless				Health status; knowledge about TB; costs; fear; disinterest

64	Szkwarko, 2016, descriptive	Kenya, street-connected youth and young adults			Trust; difficulties obtaining morning sputum; underrepresentation of women	
65	Tan, 2001, review	USA, nonimmigrant visitors			Funding and logistical difficulties; technical issues; overburdening providers	
66	Tankimovich, 2013, review	USA, homeless, migrants		Coordination and collaboration of programmes	Education and incentivization for screening participants and TB patients; accessible, affordable facilities and technology; cultural knowledge; coordination between governments, academic and private institutions	Lack of knowledge; misconceptions; contact difficulties; co-morbidities; cultural values
67	Tardin, 2009, quantitative	Switzerland, migrants				Stigma; different communication styles and health beliefs; language barrier; acceptance of diagnosis; respect towards authority; religious value; tight community network

68	Tupasi, 2000, quantitative	Philippines, community			Services free and conveniently located	Symptoms; fear of stigma
69	Welshman, 2006, review	UK, migrants			Dissatisfaction with work; disjunction between guidelines and practice; cost; logistics; administration; language barrier; shortage of radiologists/ radiographers	Changes of address; language barrier; mistrust of authorities
70	Welshman, 2006, editorial	UK, migrants			Logistics; administration; language barrier; shortages of radiologists/radiographers	Changes of address; language barrier; mistrust of authorities
71	Wright, 1979, quantitative	South Africa, high-risk			Limited and inadequate facilities for the examination of sputum; cost	"Degree of sophistication of the people involved"
72	Yassin, 2013, mixed methods	Ethiopia, contacts		Capacity strengthening; advocacy; communication; social mobilization; transport; treat-	Providers' commitment; CHWs' job satisfaction; laboratory technicians' perceived benefit of ACF; burden on laboratory; incentivization of laboratory technicians; benefit from new equipment; quality of smears prepared by CHWs	

				ment; CHW training, supervision, support		
73	Zenner, 2017, review	Worldwide, migrants	WHO End TB strategy; WHO against indiscriminate mass screening (1974)		Type of programme; target group; voluntary vs mandatory ACF	

* The article contains important information on additional influencing factors beyond the ones displayed in this table. Due to space limitations, we did not include them here.
