# Supplementary material for: Factors influencing active tuberculosis case-finding policy development and implementation: a scoping review
Source: BMJ Open. 2019 Dec 11;9(12):e031284. doi: 10.1136/bmjopen-2019-031284 (PMC6924749; doi:10.1136/bmjopen-2019-031284)
Supplement: Author's manuscript [file bmjopen-2019-031284.draft_revisions.pdf]

BMJ Open

BMJ Open is committed to open peer review. As part of this commitment we make the peer review history of every article we publish publicly available.

When an article is published we post the peer reviewers' comments and the authors' responses online. We also post the versions of the paper that were used during peer review. These are the versions that the peer review comments apply to.

The versions of the paper that follow are the versions that were submitted during the peer review process. They are not the versions of record or the final published versions. They should not be cited or distributed as the published version of this manuscript.

BMJ Open is an open access journal and the full, final, typeset and author-corrected version of record of the manuscript is available on our site with no access controls, subscription charges or pay-per-view fees (http://bmjopen.bmj.com).

If you have any questions on BMJ Open's open peer review process please email info.bmjopen@bmj.com

BMJ Open

Factors influencing active tuberculosis case finding policy development and implementation: a scoping review

Journal:	*BMJ Open*
Manuscript ID	bmjopen-2019-031284
Article Type:	Research
Date Submitted by the Author:	26-Apr-2019

Complete List of Authors:	Biermann, Olivia; Karolinska Institutet, Public Health Sciences<br>Lönnroth, Knut; Karolinska Institutet, Public Health Sciences<br>Caws, Maxine; Liverpool School of Tropical Medicine, Department of Clinical Sciences; Birat Nepal Medical Trust<br>Viney, Kerri ; Karolinska Institutet, Public Health Sciences; Australian National University, Research School of Population Health, College of Health and Medicine

Keywords:	Tuberculosis < INFECTIOUS DISEASES, active case-finding, Health policy < HEALTH SERVICES ADMINISTRATION & MANAGEMENT, policy development, policy implementation, PREVENTIVE MEDICINE

SCHOLARONE™
Manuscripts

Factors influencing active tuberculosis case finding policy development and implementation: a scoping review

Olivia Biermann[1]* (olivia.biermann@ki.se)

Knut Lönnroth[1] (knut.lonnroth@ki.se)

Maxine Caws[2 3] (maxine.caws@lstmed.ac.uk)

Kerri Viney[1 4] (kerri.viney@anu.edu.au)

1 Department of Public Health Sciences, Karolinska Institutet, Tomtebodavägen 18a, 17177 Stockholm, Sweden

2 Department of Clinical Sciences, Liverpool School of Tropical Medicine, Pembroke Pl, Liverpool L3 5QA, United Kingdom

3 Birat Nepal Medical Trust, Lazimpat, Kathmandu, Nepal

4 Research School of Population Health, College of Health and Medicine, Australian National University, Canberra ACT 2600, Australia

*Corresponding author

For peer review only - http://bmjopen.bmj.com/site/about/guidelines.xhtml

Abstract (299 words)

Background

The World Health Organization (WHO) emphasizes the importance of active case-finding (ACF) for early detection of tuberculosis (TB) to achieve the Sustainable Development Goal target to End TB

Objective

To explore antecedents, components and influencing factors on ACF policy development and implementation.

Methods

We undertook a scoping review searching four electronic databases for the period 1968 to January 2018. We included any type of study written in English. We excluded studies focusing on latent TB infection, passive case-finding, childhood TB and studies about effectiveness, yield, accuracy and impact without descriptions of how this evidence has/could influence ACF policy or implementation. We conducted frequency and thematic analyses.

Results

Seventy-three articles fulfilled our eligibility criteria. Most (67%) were published after 2010. The studies were conducted in all WHO regions, but primarily in Africa (22%), Europe (23%) and the Western-Pacific region (12%). Forty-one percent of the studies were classified as quantitative, followed by reviews (22%) and qualitative studies (12%). Most articles focused on ACF for TB contacts (25%) or migrants (32%). Fourteen percent of the articles described community-based screening of high-risk populations. Fifty-nine percent of studies reported on influencing factors for ACF implementation; mostly at the levels of the health system (e.g. resources) and the community/individual (e.g. stigma). Only two articles highlighted influencing factors for ACF policy development (e.g. politics). Articles described WHO's recent emphasis on ACF as an important antecedent of the increasing interest in ACF,

For peer review only

For peer review only - http://bmjopen.bmj.com/site/about/guidelines.xhtml

especially in low- and middle-income countries. Key components of ACF implementation include health system capacity, mechanisms for integration, education and collaboration for ACF.

Conclusion

We identified some main themes regarding the antecedents, components and influencing factors for ACF policy development and implementation. However, evidence remains scarce especially concerning policy development. More original research is required to understand, inform and improve policy processes and implementation.

Key words

Tuberculosis, active case-finding, community, policy development, policy implementation

Article summary

Strengths and limitations of this study

- Overview of some main themes regarding the antecedents, components and influencing factors for ACF policy development and implementation.
- Wide scope of influencing factors (barriers and facilitators) were reviewed.
- Reporting of ACF policy development and implementation varied in completeness across the included articles.

Manuscript (Word count: 4777 words)

Background

Systematic screening for tuberculosis (TB) is defined by the World Health Organization

For peer review only - http://bmjopen.bmj.com/site/about/guidelines.xhtml

(WHO) as the "systematic identification of people with suspected active tuberculosis (TB), in a predetermined target group, using tests, examinations or other procedures that can be applied rapidly" [1]. Active case-finding (ACF) is synonymous with systematic screening for active TB, although it usually implies screening outside of health facilities.

Tuberculosis is a major global health challenge, particularly in low- and middle-income countries. The WHO End TB Strategy and the Sustainable Development Goals aim at finding the "missing cases" and ending the global TB epidemic by 2030. This will require intensified activity to increase TB case detection, specifically in "hard-to-reach" groups [2]. In 2017, there was a 4.1 million gap between estimated incident and notified TB cases globally, reflecting a combination of underreporting of detected TB cases and underdiagnosis [3]. Many people with TB are diagnosed only after long delays [4], causing suffering and economic hardship, and sustaining transmission [1].

Active case finding is mostly provider-initiated. It targets people in high-risk groups who may not seek health care actively [1]. Potential benefits for patients include reduced morbidity, mortality and economic consequences due to earlier diagnosis, while society can benefit from reduced transmission and a reduced burden of TB, which often affects the most economically productive members of a society. TB screening in high-risk groups has been implemented in many settings and can significantly improve TB case notification [5-7].

However, if not well targeted and implemented, ACF can be costly leading to diversion and waste of scarce resources, potentially compromising passive case-finding infrastructure and weakening health systems. It can also cause harm to individuals, e.g. by increasing the risk of a false positive diagnosis, or increased stigma and discrimination [1,2].

For peer review only - http://bmjopen.bmj.com/site/about/guidelines.xhtml

Questions remain about both *if* ACF in general is worthwhile, and *how* to best plan and implement outreach screening through ACF in a given context as a synergistic, not parallel system. The evidence base is weak concerning the benefits and cost-effectiveness of ACF and how it varies between risk groups [8]. There is weak evidence for choosing the best ACF approach, and few head-to-head trials of ACF approaches have been conducted. Many differences exist in stakeholder's values and preferences concerning the rationale, outcomes and possible secondary effects of ACF. Despite the relatively weak evidence, WHO has a guideline on systematic screening and the Global Fund [9] and TB REACH [10] provide increasing funding for ACF, while a growing number of countries are increasing investment in and have national policies for ACF.

The aim of this study was to explore antecedents, components and influencing factors for ACF policy development and implementation, to inform and improve future ACF policy processes. We did not aim to review the evidence on ACF per se; therefore, we explore yield, accuracy and impact only in the context of how they influence ACF policy development or implementation.

Methods

We conducted a scoping review, using an a-priori protocol based on the following research question: *Which are the antecedents, components and influencing factors (barriers and facilitators) in developing and implementing ACF policies?* The protocol was based on guidance from the Joanna Briggs Methods Manual for Scoping Reviews [11]. The inclusion criteria, the screening manual and the data-charting table were developed by one author (OB), reviewed by two authors (KV and KL) to ensure face validity, and pilot-tested. All team

members were consulted at various stages of the scoping review to provide input on the search, data extraction and charting, and the interpretation of the results. The charted data was summarized in tables, and then condensed resulting in Tables 1 and 2. The Preferred Reporting Items for Systematic reviews and Meta-Analyses Extension for Scoping Reviews (PRISMA-ScR) was used to guide reporting [12]. The completed checklist is available in *Additional file 1.* The protocol is available from the corresponding author upon request.

Data sources and search

We searched MEDLINE, Web of Science, The Cochrane Database of Systematic Reviews and the WHO Library from 1968 until January 2018. The search strategy was developed in collaboration with a medical librarian from Karolinska Institutet (Carl Gornitzki), and further refined through team discussion. The search strategy for MEDLINE is available in *Additional file 2*. The latter was standardized but adapted to fit the other database searches. The respective search strategies are available from the corresponding author upon request. The results of the literature search were imported into Rayyan.

Inclusion criteria

Eligible studies focused on active TB disease. The review included studies describing or analysing ACF policy development and implementation. Facilitators and barriers linked to access or treatment were included, if there was a clear link to ACF. Furthermore, the review included studies analysing the use of evidence in ACF policy development and implementation. Studies of any design, conducted in any setting or country, and those published in the English language were eligible for inclusion. We excluded studies focusing only on latent TB infection, passive case-finding and childhood TB, and studies about effectiveness, yield, accuracy and impact without descriptions of how this evidence has/could

For peer review only - http://bmjopen.bmj.com/site/about/guidelines.xhtml

influence ACF policy.

Screening, data abstraction and charting

One reviewer (OB) initially reviewed titles to remove duplicates, and those studies not focusing on TB. Two reviewers (OB and KV) independently reviewed titles and abstracts, and then full-text articles for inclusion. Conflicts were resolved through discussion, based on the inclusion/exclusion criteria.

The data-charting table was developed in Microsoft Excel. An abridged version of the table is available in *Additional file 3*. Data was extracted on study design, country, target population for ACF, benefits of ACF, risks of ACF, ACF antecedents, ACF policy development and implementation (use of evidence, country-level to individual-level facilitators and barriers, stakeholders involved), lessons learned, future perspectives and future research. Relevant data was charted by one author (OB), while a second author (KV) verified this step by charting data from a random sample of studies for comparison. Differences in data-charting were resolved by discussion.

Patient and public involvement

This study is related to a qualitative study of experts and a survey of National TB Programme managers combined with a policy document review on the topic of ACF policy development and implementation which are currently under way to harness tacit knowledge on the topic. The research questions and variables for these studies were informed by the results of this scoping review. The results of this study will be presented to researchers and policy-makers in the field via targeted issue briefs. We will also share the results with the public via a video and short messages on social media.

For peer review only - http://bmjopen.bmj.com/site/about/guidelines.xhtml

Results

Literature search

After screening 2943 titles and abstracts and 271 full-text articles, 73 unique articles fulfilled our eligibility criteria (*Figure 1*) [13-19,22-59,64-68,70-92]. The reasons for excluding full-text articles are provided in Fig. 1.

Characteristics of the included articles (*n*=73)

The breadth of studies identified reflect the complexity and diversity of ACF policies and their characteristics. There has been a gradual increase in the number of articles on ACF since the first identified (e.g. from 1979), and 67% (*n*=49/73) were published in the period 2010-2018 (*Table 1*). The studies were conducted in all WHO regions, i.e. the African region (22%, *n*=16/73), the Americas (North America) (14%, *n*=10/73), the Eastern Mediterranean region (1%, *n*=1/73), the European region (23%, *n*=17/73), South-East Asia (7%, *n*=5/73) and the Western Pacific region (12%, *n*=9/73). The most common types of articles were classified as quantitative papers (41%, *n*=30/73), reviews (22%, *n*=16/73) and qualitative studies (12%, *n*=9/73). The most frequent ACF target groups were contacts (25%, *n*=18/73) and migrants from high-incidence countries (32%, *n*=23/73). Fourteen percent (*n*=10/73) of the articles focused on screening of defined communities.

Table 1 Characteristics of the articles included in the scoping review (n=73)

(Please see end of text file.)

Fig 1. PRISMA flow diagram

(Please see separate Word file entitled "Biermann_fig 1".)

For peer review only

Antecedents

Active case-finding has been implemented for many decades primarily in high-income countries, starting with mass screening campaigns in the general population in the 1950s and 1960s, then moving towards specific risk populations in recent decades, such as migrants from high-incidence countries and prison populations [13]. In low- and middle-income countries, the interest in ACF has increased in recent years, mainly as a response to a sustained case detection gap documented in annual Global TB Reports produced by WHO [3] and the emergence of new WHO guidelines [14].

Generally, TB programmes have moved from traditionally vertical approaches and exclusively facility-based case finding among people seeking care with TB symptoms (so-called “passive case-finding”) towards being closer to the client through decentralized, community-based solutions and outreach activities, including ACF – similar to Human Immunodeficiency Virus (HIV) programmes [15,16].

In 1976, the WHO Expert Committee on TB recommended that countries abandon *indiscriminate* mobile mass radiography, as evidence showed the inefficiency of population-wide screening in settings that had seen TB rates drop dramatically since the second World War [17]. It should be noted that the Expert Committee emphasized that TB screening should still be done in selected risk groups [17,18].

The current WHO guidelines on systematic screening for active TB were released in 2013 [1]. These guidelines still discourage *indiscriminate* mass screening and strongly recommend ACF only in selected high-risk groups, while conditionally recommending screening in other high-risk groups [19]. The 2013 WHO guidelines – reflected in the latest 2015 WHO’s End TB Strategy [2] and aligned with the Sustainable Development Goals – put ACF back on the

For peer review only

agenda. The guidelines provide principles for how to cautiously experiment with and expand an approach that had been largely missing in previous global TB strategies, including the Directly Observed Treatment, Short Course (DOTS) Strategy from 1994 [20] and the Stop TB Strategy 2006-2015 [21]. Moreover, the guidelines emphasize the importance of basing ACF policy on local epidemiology and to perform careful evaluations to improve the ACF evidence base [18]. Today, ACF is considered a global health priority [22,23].

Components

The building blocks of ACF are multifaceted and well-described in the literature, and so are the components of the wider health system that ACF depends on. It is important for high-quality diagnosis, treatment, care, management, patient support and capacity for scale-up to be available [17]. Risk groups must be prioritized based on assessments of benefits and risks, feasibility, acceptability, the number needed to screen and the cost-effectiveness of ACF [17], while also considering the heterogeneity of the risk group [24].

The overall ACF approach must be flexible, patient-centred [14,25] and culturally sensitive [26]. In resource poor settings, ACF is often backed by multi-channel financing mechanisms, including from international donors [27]. Record-keeping, monitoring and evaluation are described as being essential [28,29]. Incentives are sometimes deemed necessary [23,28,30,31,32], e.g. for identifying cases [31], and in the form of cell phone airtime, food or money [23]. The articles also emphasized ACF-specific health education as paramount – for patients [19,33,34,35], their families [36], providers [25] and community health workers (CHW) [37,38]. The training of CHWs should be followed by supervision [37,38]. In addition, community involvement [34,38,39,40] and social mobilization [31] are major parts of ACF. One study described how the involvement of community leaders was a key component to improve trust among patients and contacts [35].

For peer review only - http://bmjopen.bmj.com/site/about/guidelines.xhtml

Lastly, the studies underlined how vital it is to collaborate with other disease programmes [40,41,42], agencies and sectors [27,29], and to integrate ACF within available systems [17,43] and services to avoid vertical and stand-alone implementation structures [26,38,44]. The latter includes collaboration between providers and community-based organizations [40], and between HIV and TB services [32].

Influencing factors for ACF policy development

Active case-finding policy development processes were not well described in the literature. Only two articles reported facilitators and/or barriers for ACF policy development: politics [45,46] and laws [46]. Welshman [45] describes how, in the mid-1950s, the Ministry of Health in the United Kingdom subverted pressure from stakeholders who were in favour of compulsory ACF at ports of entry – the Ministry "argued that the problem [of TB in migrants] was a minor one" so a policy was not developed. On the other hand, laws such as the 1958 Commonwealth Migration Act were described, which made screening compulsory, and thus influenced ACF policy development in the United Kingdom [46].

Influencing factors for ACF policy implementation

Policy implementation processes in the area of ACF are widely researched. In total 43, articles reported barriers and/or facilitators for ACF implementation (*Table 2*). Most articles mentioned factors at the level of the health system, as well as the individual and community level.

Table 2 Barriers and facilitators for active case-finding policy implementation (n=43)

(Please see end of text file.)

For peer review only - http://bmjopen.bmj.com/site/about/guidelines.xhtml

Health system level

It is fundamental to consider health system factors when putting ACF policy into practice and identifying which factors influence ACF policy implementation the most? We derived six major themes from the articles: 1) availability of financial resources, 2) existing systems and structures, 3) availability of diagnostic tests, 4) staff experience and motivation, 5) collaboration between different actors, and 6) implementation of a person-centred approach.

Availability of financial resources

Many articles (35%, *n*=15/43) mentioned financial resources as an influencing factor for ACF policy implementation. Finances were described as being scarce at different levels of the health system [36], for the provision of staff [34,38], drugs and equipment [47].

Existing systems and structures

Existing systems and structures were reported as influencing factors for ACF policy implementation by 26% (*n*=11/43) of the articles. Studies elaborated on the availability of health and social services [28,17] and laboratory networks [22].

Availability of diagnostic tests

Nineteen percent (*n*=8/43) of the articles elaborated on the availability of diagnostic tests and services as key influencing factor for ACF policy implementation – with many articles referring to the availability of molecular diagnostic tests such as Xpert MTB/RIF® [29,48,49,50]. Decentralization of health care services, national coverage of diagnostics and task sharing were perceived as facilitators of ACF policy implementation, but unavailable in many settings [41].

Staff experience and motivation

Thirty-three percent (*n* =14/43) of the studies described staff experience and motivation as

For peer review only - http://bmjopen.bmj.com/site/about/guidelines.xhtml

influencing factors for ACF policy implementation. One study elaborated on how, on the one hand, staff members would feel motivated by increased case detection and awareness-raising through ACF. On the other hand, they would be frustrated when being “shouted at, ignored, (and) harassed” by target communities, in this case people living in poor urban settlements [34]. The over-burdening of staff was highlighted by 16% (*n*=7/43) of the articles, e.g. leading to absenteeism [51].

Collaboration between different actors

A variety of studies (16%, *n*=7/43) described collaboration as an important influencing factor for ACF policy implementation. One example is the collaboration between community workers, health centre staff and laboratory technicians [14].

Implementation of a person-centred approach

The need for a person-centred approach to ACF policy implementation was referred to by 19% (*n*=8/43) of the articles, e.g. adopting approaches including being non-judgmental and supportive [52] and assuring confidentiality [23,53]. Another 12% (*n*=5/43) of the articles emphasized health education as a necessity.

Individual and community

Active case-finding policies should be *for* individuals and communities and their influence on successful implementation is elaborately described in the literature. Three overarching themes emerged from the studies: 1) stigma and discrimination, 2) individual characteristics and socio-cultural factors, and 3) knowledge and awareness.

Stigma and discrimination

Many studies (37%, *n*=16/43) reported stigma and discrimination linked to TB as influencing factors for ACF policy implementation. The consequences could be a contact’s avoidant behaviour, such as providing incorrect phone numbers and/or wrong directions to their homes

For peer review only

For peer review only - http://bmjopen.bmj.com/site/about/guidelines.xhtml

[28], or misconceptions about TB [25,40]. Closely linked was the issue of fear, which could be related to TB itself [24,34,52], the ACF process [54], or to deportation in some cases of migrant screening [33,42,55,56]. Studies also described trust as key for ACF policy implementation [37,51] – not only for early diagnosis, but for the sustained commitment to addressing social determinants of TB [57].

Individual characteristics and socio-cultural factors

Twenty-eight percent (*n*=12/43) of the articles mentioned the social characteristics of the persons screened as key influencing factors for ACF policy implementation, e.g. for ensuring equitable access to care [22,34,58] and promoting conducive health-seeking behaviour [24,37,40]. Moreover, the articles frequently reported socio-cultural factors and language (33%, *n*=14/43) as influencing factors for ACF policy implementation. Culture was commonly described as a barrier to ACF policy implementation [37,42,59], e.g. if cultural beliefs would negatively influence the receptiveness of community health workers' advice. Only one study mentioned culture as a facilitator for putting ACF into practice, i.e. if culture meant respect toward professional authority [59].

Knowledge and awareness

Lastly, 19% (*n*=8/43) of the articles reported factors related to knowledge and awareness as influencing factors for ACF policy implementation. One such factor could be the understanding of the purpose of ACF [25]. Also, knowledge and awareness are closely linked to persisting stigma and discrimination, e.g. Ayakaka et al [28] describe how health workers attributed TB-associated stigma to a lack of general knowledge in the community about TB.

Discussion

For peer review only - http://bmjopen.bmj.com/site/about/guidelines.xhtml

Characteristics of the included articles

We identified 73 articles addressing antecedents, components and/or influencing factors for ACF policy development and implementation. The included studies originated from all WHO regions. Africa and Asia are home to 90% of the 30 high TB burden countries (53% and 37% respectively) [60], which may explain why studies were more frequently conducted from these continents: 22% of the studies were conducted in Africa, 12% in the Western-Pacific region and 7% in South-East Asia. Studies conducted in the European region (23%) were predominantly about different forms of migrant screening. There was no study from Latin America among the 73 included articles, and there were no articles in the Spanish language among those excluded.

Most studies were published after 2010, which may be linked to the publication of the WHO guidelines on systematic screening in 2013 [1]. We classified 41% of the articles as quantitative, e.g. cross-sectional and cohort studies, effectiveness and cost-effectiveness studies, randomized controlled trials and programme evaluations. The next most common study types were reviews (22%) and qualitative studies (12%). The first qualitative studies on our topic were published in 2003 [25,55]. We only identified two mixed-methods studies, one published in 2013 [38] and one in 2015 [34]. The mixed-methods design combined with quantitative ACF evaluation might become more commonly used to explore the complexity of ACF policy development and implementation in the future, as it has the potential to both increase contextual understanding and reduce biases.

The evidence base is not only growing, but diverse. This implies that while many lessons can be learned across different settings, some might not be generalizable. Amongst the broad range of possible target groups for ACF, most of the articles focused either on TB contacts or migrants. The focus on the latter was mainly in low-incidence countries and may be a result of long-standing national health and migration policies supporting these types of screening. Yet,

For peer review only - http://bmjopen.bmj.com/site/about/guidelines.xhtml

other studies covered vulnerable groups, such as homeless people, people living with HIV or people living in congregate settings, as well as the urban poor. Some articles included analysis of more than one high-risk group. There were no studies on indiscriminate mass screening. However, there were studies targeting broad communities, e.g. communities with high HIV prevalence.

Antecedents

Despite the growing number of studies, there is still a relative lack of evidence about the health benefits for individuals, the epidemiological impact and cost-effectiveness of ACF. Nonetheless, the interest in ACF has clearly increased in recent years. The persistent case detection gap, the 2013 WHO guidelines on systematic screening [1], and ACF being mentioned as a core component of the End TB Strategy [2] seem to have motivated countries to include ACF in national TB strategies. Universal Health Coverage and the Sustainable Development Goals have only marginally been mentioned as important for stepping up the TB response [41]. However, aiming to “leave no one behind”, they might be yet another part of the explanation for the greater interest in ACF.

Not having enough evidence to drive ACF policy development and implementation means that decisions to embark on ACF rely largely on stakeholders’ tacit knowledge, experience, values and preferences. Demonstrated screening yield in the same or other settings could also motivate further ACF implementation, although the number of cases detected may not be a relevant public health impact measure in itself. The above-mentioned WHO guidelines use the term “indirect evidence” [1]. Strong “direct evidence” exists that early diagnosis and correct TB treatment reduce morbidity, mortality and transmission. This can constitute, for example, “indirect evidence” for ACF, based on the logic that if ACF leads to early detection and treatment then it should also lead to better health outcomes and less transmission, despite the

For peer review only - http://bmjopen.bmj.com/site/about/guidelines.xhtml

398 lack of trial data demonstrating a direct health impact of ACF. Similar reasoning can be used to describe potential harms associated with ACF, e.g. based on modelling of the risk of false positive TB diagnoses in different epidemiological contexts. “Indirect evidence”, tacit knowledge, experience, values and preferences are likely to be important antecedents for national policy.

404 **Components**

405 The components of ACF policy, and of the system which ACF is part of, are complex. The articles reported on the prerequisites of the given system, its resources, capacity and tools, but also elaborated on the need for integration, education and engagement for ACF implementation to succeed. All components seem indispensable for successful ACF policy development and implementation. Yet, in real-life settings, rarely all components are in place. Which ACF components are more dispensable than others? The 2013 WHO guidelines on systematic screening list six key principles for screening for active TB, the first principle being: “Before screening is initiated, high-quality TB diagnosis, treatment, care, management and support for patients should be in place, and there should be the capacity to scale these up further to match the anticipated rise in case detection that may occur as a result of screening“ [1]. Yet, even this very first principle might not be completely adhered to, often due to a lack of resources, before a country embarks on ACF policy development and implementation.

418 **Influencing factors**

420 *Scarce research on ACF policy development*

421 Our findings indicate a paucity of research focusing specifically on ACF policy development.

For peer review only - http://bmjopen.bmj.com/site/about/guidelines.xhtml

The only two factors described – politics and laws – were mentioned by the same author in two different articles and concerned migrant TB screening in a low-incidence country. Even though not documented as such in the literature, data on the epidemiological situation of a country and local evidence regarding impact are assumed to be key factors influencing ACF policy development. These types of data and evidence are needed to identify high-risk groups [61] and devise appropriate screening algorithms. An assessment of the epidemiological situation might be based on data from regularly collected datasets or prevalence surveys, and WHO has developed a tool to help countries estimate screening yield and costs based on such data to identify appropriate risk groups and approaches [62].

Interestingly, the evolution of the funding landscape for ACF, e.g. through TB REACH, has not been explicitly mentioned as an influencing factor for ACF policy development or implementation, though it has been a likely game-changer. TB REACH is a case-finding initiative funded largely by Global Affairs Canada and coordinated by the Stop TB Partnership [63]. The initiative has not only funded ACF activities but raised awareness about the importance of ACF. It has contributed to the local evidence base, while the uptake of the evidence generated by TB REACH may be considered but not mentioned in the studies included in this review. Four articles mentioned TB REACH as funder of their research, with the disclaimer that the initiative had no role in study design, data collection and analysis [14,38,44,64]. Other publications acknowledged its role in implementing ACF when describing the study methods, e.g. that TB REACH funded door-to-door screening [34] or the implementation of different ACF models [65] and that the initiative upgraded an ACF strategy by introducing new diagnostics (e.g. Xpert MTB/RIF®) [50]. It is important to reflect on and document the influence that TB REACH, other international funding mechanisms and technical agencies have on ACF policy development and implementation.

For peer review only - http://bmjopen.bmj.com/site/about/guidelines.xhtml

ACF policy and social determinants of health

Many barriers and facilitators for implementing ACF policies lie at the level of communities and individuals. These factors include social determinants such as the educational level [28,66], employment status [29,67] and access to care [68] of the target population. These factors are inextricably linked and downwardly additive, with the power to limit an individual's or a community's participation in ACF. ACF policies cannot be put in practice successfully if implementation barriers prevail. However, rather than talking about "missing cases" and communities that are "hard to reach", it may be useful to consider if health services are missing or hard to reach instead.

Individuals or communities may be ACF target groups precisely due to the described vulnerabilities. As such, ACF could be a way to by-pass access barriers, e.g. by using mobile units. Yet, even when designing services to be more accessible, target populations may still not come forward, take the next step of referral to complete the diagnostic pathway or adhere to treatment due to socioeconomic barriers.

Active case-finding polices thus must be designed and implemented with careful consideration of social determinants in a given context to avoid possible harm. Nevertheless, ACF tends to be designed as a short cut to reaching the poor, since addressing underlying determinants "upstream" and health systems strengthening are considered more complex and time-consuming. Whitehead et al [69] described how interventions designed to help vulnerable populations may be "implemented in such a way as to stigmatize the very people the programme was designed to help and, in so doing, push them to avoid the help on offer." In addition, a comprehensive approach in developing and implementing ACF policies is required that includes health education as an important component to ACF policies, and that ensures collaboration between key stakeholders, not only between patients and providers, but

For peer review only - http://bmjopen.bmj.com/site/about/guidelines.xhtml

between providers and local health authorities, between agencies, and across sectors and disease programmes.

Finally, although this review focused on the factors that influence ACF policy and implementation, it is important to also reflect on how ACF policy influences its context in return, e.g. not only factors at the level of the community and the individual, but at the level of the health system and beyond. How may the ACF policy increase or decrease stigma in a community? How may ACF policy influence the human and financial resources available in a health system? Only then may we see the real impact of ACF and be able to make equitable decisions in policy development and implementation processes.

Future research

This scoping review demonstrated that while we know much about facilitators and barriers for ACF policy implementation, we know less about how to strengthen those facilitators and how to overcome those barriers. A major knowledge gap remains when it comes to understanding which contextual factors influence ACF policy development.

Limitations

Our scoping review has some limitations. To increase the feasibility, we limited the search to MEDLINE, Web of Science, The Cochrane Database of Systematic Reviews and the WHO Library. We did not do a critical appraisal of the individual sources of evidence or within sources of evidence, nor did we describe sources of funding for the included sources of evidence. We did not search grey literature, nor the references of included studies. We did not contact authors for more information. We also limited the inclusion to studies written in English, which may have resulted in the exclusion of eligible studies in other languages, e.g. Spanish. Furthermore, the results from this scoping review are only up to date as of 31

For peer review only - http://bmjopen.bmj.com/site/about/guidelines.xhtml

January 2018. The data were extracted by one reviewer only. However, the data are likely valid, as a pilot-test was conducted prior to embarking on data extraction with members of the study team. A second reviewer verified a random selection of the data.

Another limitation was that the included articles often did not distinguish between the implementation of an ACF policy versus the implementation of an ACF programme or intervention. This is likely due to inconsistent use of the terms in the literature. As such, our results are likely applicable to both. Furthermore, the reporting of ACF policy development and implementation varied in their completeness across the included articles, and as such, our data are limited by the details described in the literature, e.g. most papers described steps to implementation, but did not provide details on non-response or unsuccessful practices. There is a risk of publication bias.

Conclusions

We identified some main themes regarding the antecedents, components and influencing factors for ACF policy development and implementation. However, evidence remains scarce especially concerning policy development. More original research on ACF is required to understand, inform and improve policy processes and implementation.

List of abbreviations

ACF – Active Case-Finding

ART – Antiretroviral Therapy

CHW – Community Health Worker

DOTS – Directly Observed Treatment, Short Course

HIV – Human Immunodeficiency Virus

LTBI – Latent Tuberculosis Infection

For peer review only - http://bmjopen.bmj.com/site/about/guidelines.xhtml

PCF – Passive Case-Finding

PRISMA - Preferred Reporting Items for Systematic reviews and Meta-Analyses

PRISMA-ScR – Preferred Reporting Items for Systematic reviews and Meta-Analyses extension for Scoping Reviews

TB – Tuberculosis

TiAb – Title and abstract

WHO – World Health Organization

Ethics approval and consent to participate

N/A

Consent for publication

N/A

Availability of data and material

The materials supporting the conclusions of this article are included within the article and its additional files.

Additional files

For peer review only - http://bmjopen.bmj.com/site/about/guidelines.xhtml

Additional file 1: PRISMA Extension for Scoping Reviews (PRISMA-ScR) checklist.

Additional file 2: Literature search strategy used for Medline; additional search strategies available from the authors.

Additional file 3: Abridged data charting table.

Competing interests

We have no competing interests to declare.

Funding

This work was supported by the EU-Horizon 2020-funded IMPACT-TB project (grant 733174).

Author contributions

OB, KL, MC and KV conceived the study. OB developed the search strategy together with a medical librarian from Karolinska Institutet. OB and KV screened titles and abstracts, and then full-text articles. OB extracted, charted, analysed and interpreted the data. KV verified the data extraction by charting data from a random sample of studies for comparison. OB wrote the first draft of the manuscript. KL, MC and KV were consulted at various stages of the scoping review to provide input on the search strategy, data extraction, charting, analysis and the interpretation of the results, and provided comments on the manuscript. All authors read and approved the final manuscript.

For peer review only - http://bmjopen.bmj.com/site/about/guidelines.xhtml

Acknowledgements

OB is funded by the EU-Horizon 2020-funded IMPACT-TB project (grant 733174). The authors thank Carl Gornitzki, medical librarian at Karolinska Institutet, for supporting the development of the search strategy, and Gabriella Ekman, writing instructor at Karolinska Institutet's library for her valuable feedback in writing this manuscript.

References

1. World Health Organization. Systematic screening for active TB: principles and recommendations. Switzerland, Geneva: World Health Organization; 2013.
2. World Health Organization. Implementing the End TB Strategy: the essentials. Switzerland, Geneva: World Health Organization; 2015.
3. World Health Organization. Global TB Report 2017. Switzerland, Geneva: World Health Organization; 2017.
4. Storla DG, Yimer S, Bjune GA. A systematic review of delay in the diagnosis and treatment of TB. BMC Public Health. 2008;8:15. doi: 10.1186/1471-2458-8-15.
5. Blok L, Sahu S, Creswell J, Alba S, Stevens R, Bakker MI. Comparative meta-analysis of TB contact investigation interventions in eleven high burden countries. PLoS One. 2015;10:3:e0119822. doi: 10.1371/journal.pone.0119822.
6. Creswell J, Codlin AJ, Andre E, Micek MA, Bedru A, Carter EJ, et al. Results from early programmatic implementation of Xpert MTB/RIF testing in nine countries. BMC Infect Dis. 2014;14:2. doi: 10.1186/1471-2334-14-2.

For peer review only

For peer review only - http://bmjopen.bmj.com/site/about/guidelines.xhtml

7. Creswell J, Sahu S, Blok L, Bakker MI, Stevens R, Ditiu L. A multi-site evaluation of innovative approaches to increase TB case notification: summary results. PLoS One. 2014;9:4:9:e94465. doi: 10.1371/journal.pone.0094465.

8. Kranzer K, Afnan-Holmes H, Tomlin K, Golub JE, Shapiro AE, Schaap A, et al. The benefits to communities and individuals of screening for active TB disease: a systematic review. Int J Tuberc Lung Dis. 2013;17:432–46. doi: 10.5588/ijtld.12.0743.

9. Global Fund, World Health Organization and Stop TB Partnership Strategic Initiative. https://www.who.int/tb/joint-initiative/en/ (2019). Accessed 8 Apr 2019.

10. TB REACH. http://www.stoptb.org/global/awards/tbreach/ (2019). Accessed 8 Apr 2019.

11. Joanna Briggs Institute. Methodology for JBI scoping reviews. Adelaide: Joanna Briggs Institute; 2015.

12. Tricco A, Lillie E, Zarin W, O'Brien KK, Colquhoun H, Levac D, et al. PRISMA Extension for Scoping Reviews (PRISMA-ScR): Checklist and Explanation. Ann Intern Med. 2018;169:467-473. doi: 10.7326/M18-0850.

13. Eang MT, Satha P, Yadav RP, Morishita F, Nishikiori N, van-Maaren P, Lambregts-van Weezenbeek C. Early detection of tuberculosis through community-based active case finding in Cambodia. BMC Public Health. 2012;12:469. doi: 10.1186/1471-2458-12-469.

14. Lorent N, Choun K, Thai S, Kim T, Huy S, Pe R, et al. Community-Based Active Tuberculosis Case Finding in Poor Urban Settlements of Phnom Penh, Cambodia: A Feasible and Effective Strategy. PLoS One. 2014;9:3:e92754. doi: 10.1371/journal.pone.0092754.

15. Godfrey-Fausset P, Ayles H. Can we control tuberculosis in high HIV prevalence settings? Tuberculosis. 2003;83:68-76. doi: 10.1016/S1472-9792(02)00083-5.

For peer review only - http://bmjopen.bmj.com/site/about/guidelines.xhtml

16. Corbett EL, MacPherson. Tuberculosis screening in high human immunodeficiency virus prevalence settings: turning promise into reality. Int J Tuberc Lung Dis. 2013;17:9:1125-1138. doi: 10.5588/ijtld.13.0117.

17. Lönnroth K, Corbett E, Golub J, Godfrey-Faussett P, Uplekar M, Weil D, Raviglione M. Systematic screening for active tuberculosis: rationale, definitions and key considerations. Int J Tuberc Lung Dis. 2013;17:3:289-298. doi: 10.5588/ijtld.12.0797 STATE.

18. Zenner D, Hafezi H, Potter J, Capone S, Matteelli A. Effectiveness and cost-effectiveness of screening migrants for active tuberculosis and latent tuberculosis infection. Int J Tuberc Lung Dis. 2017;21:9:965-976. doi: 10.5588/ijtld.16.0935.

19. Fox GJ, Loan LP, Nhung NV, Loi NT, Sy DN, Britton WJ, Marks GB. Barriers to adherence with tuberculosis contact investigation in six provinces of Vietnam: a nested case-control study. BMC Infect Dis. 2015;15:103. doi: 10.1186/s12879-015-0816-0.

20. World Health Organization. What is DOTS? A Guide to Understanding the WHO-recommended TB Control Strategy Known as DOTS. Switzerland, Geneva: World Health Organization; 1999.

21. World Health Organization. Building on and enhancing DOTS to meet the TB-related Millennium Development Goals. Switzerland, Geneva: World Health Organization; 2006.

22. Golub JE, Mohan CI, Comstock GW, Chaisson RE. Active case finding of tuberculosis: historical perspective and future prospects. Int J Tuberc Lung Dis. 2005;9:11:1183-1203. [PMID: 16333924]

23. Kerrigan D, West N, Tudor C, Hanrahan C, Lebina L, Msandiwa, et al. Improving active case finding for tuberculosis in South Africa: informing innovative

For peer review only

For peer review only - http://bmjopen.bmj.com/site/about/guidelines.xhtml

implementation approaches in the context of the Kharitode trial through formative research. Health Res Policy Sys. 2017;15:42. doi: 10.1186/s12961-017-0206-8.

24. Swigat V, Kolb R. Homeless persons' decisions to accept or reject public health disease-detection services. Public Health Nurs. 2004;21:2:162-170. doi: 10.1111/j.0737-1209.2004.021210.x.

25. Shrestha-Kuwahara R, Wilce M, DeLuca N, Taylor Z. Factors associated with identifying tuberculosis contacts. Int J Tuberc Lung Dis. 2003;7:12:S510-516

26. de Vries SG, Cremers AL, Heuvelings CC, Greve PF, Visser BJ, Bélard S, et al. Barriers and facilitators to the update of tuberculosis diagnostic and treatment services by hard-to-reach populations in countries of low and medium tuberculosis incidence: a systematic review of qualitative literature. Lancet Infect Dis. 2017;17:e128-43. doi: 10.1016/S1473-3099(16)30531-X.

27. Li J, Liu X-Q, J S-W, Li X, Yu F, Wang Y, et al. Improving tuberculosis case detection in underdeveloped multi-ethnic regions with high disease burden: a case study of integrated control program in China. Infect Dis Poverty. 2017;6:151. doi: 10.1186/s40249-017-0365-4

28. Ayakaka I, Ackerman S, Ggita JM, Kajubi P Dowdy D, Haberer EF, et al. Identifying barriers to and facilitators of tuberculosis contact investigation in Kampala, Uganda: a behavioral approach. Implementation Sci. 2017;12:33. doi: 10.1186/s13012-017-0561-4.

29. Baxter S, Goyder E, Chambers D, Johnson M, Preston L, Booth A. Interventions to improve contact tracing for tuberculosis in specific groups and in wider populations: an evidence synthesis. Health Services and Delivery Research. 2017;5:1. doi: 10.3310/hsdr05010.

For peer review only - http://bmjopen.bmj.com/site/about/guidelines.xhtml

30. Van den Bosch CA, Roberts JA. Tuberculosis screening of new entrants; how can it be made more effective? Journal Public Health Med. 2000;22:2:220-223.

31. Hinderaker SG, Rusen ID, Chiang C-Y, Yan L, Heldal E, Enarson DA. The FIDELIS initiative: innovative strategies for increased case finding. Int J Tuberc Lung Dis. 2011;15:1:71-76.

32. Kranzer K, Lawn SD, Meyer-Rath G, Vassall A, Raditlhalo E, Govindasamy D, et al. Feasibility, Yield, and Cost of Active Tuberculosis Case Finding Linked to a Mobile HIV Service in Cape Town, South Africa: A Cross-sectional Study. PLoS Med. 2012;9:8:e1001281. doi: 10.1371/journal.pmed.1001281.

33. Kulane A, Ahlberg BM, Berggren I. "It is more than the issue of taking tablets": The interplay between migration policies and TB control in Sweden. Health Policy. 2010;97:26-31. doi: 10.1016/j.healthpol.2010.02.014.

34. Lorent N, Choun K, Malhotra S, Koeut P, Thai S, Khun KE, et al. Challenges from Tuberculosis Diagnosis to Care in Community-Based Active Case Finding among the Urban Poor in Cambodia: A Mixed-Methods Study. PLoS One. 2015;10:7:e0130179. doi: 10.1371/journal.pone.0130179.

35. Tupasi TE, Radhakrishna S, Co VM, Villa MLA, Quelapio MID, Mangubat NV, et al. Bacillary disease and health seeking behavior among Filipinos with symptoms of tuberculosis: implications for control. Int J Tuberc Lung Dis. 2000;4:12:1126-1132.

36. Karki B, Kittel G, Bolokon I, Duke T. Active community-based case finding for tuberculosis with limited resources: estimating prevalence in a remote area of Papua New Guinea. Asia Pac J Public Health. 2017;28:1:17-27. doi: 10.1177/1010539516683497.

37. Getnet F, Hashi A, Mohamud S, Mowlid H, Klinkenberg E. Low contribution of health extension workers in identification of persons with presumptive pulmonary

For peer review only - http://bmjopen.bmj.com/site/about/guidelines.xhtml

684 tuberculosis in Ethiopian Somali Region pastoralists. BMC Health Serv Res.
685 2017;17:193. doi: 10.1186/s12913-017-2133-3.

686 38. Yassin MA, Datiko DG, Tulloch O, Markos P, Aschalew M, Shargie EB, et al.
687 Innovative community-based approached doubled tuberculosis case notification and
688 improve treatment outcome in Southern Ethiopia. PLoS One. 2013;8:5:e63174. doi:
689 doi:10.1371/journal.pone.0063174.

690 39. Cook VJ, Shah L, Gardy J. Modern contact investigation methods for enhancing
691 tuberculosis control in Aboriginal communities. Int J Circumpolar Health.
692 2012;71:18643. doi: 10.3402/ijch.v71i0.18643.

693 40. Seedat F, Hargreaves S, Friedland JS. Engaging New Migrants in Infectious Disease
694 Screening: A Qualitative Semi-Structured Interview Study of UK Migrant Community
695 Health-Care Leads. PLoS One. 2014;9:10:e108261. doi:
696 10.1371/journal.pone.0108261.

697 41. Suthar AB, Zachariah R, Harries AD. Ending tuberculosis by 2030: can we do it? Int J
698 Tuberc Lung Dis. 2016;20:9:1148-1154. doi: 10.5588/ijtld.16.0142.

699 42. Tankimovich M. Barriers to and interventions for improved tuberculosis detection and
700 treatment among homeless and immigrant populations: a literature review. J
701 Community Health Nurs. 2013;30:83-95. doi: 10.1080/07370016.2013.778723.

702 43. Sekandi JN, Dobbin K, Oloya J, Okwera A, Whalen CC, Corso PS. Cost-Effectiveness
703 Analysis of Community Active Case Finding and Household Contact Investigation for
704 Tuberculosis Case Detection in Urban Africa. PLoS One. 2015;10:2:e0117009. Doi:
705 10.1371/journal.pone.0117009.

706 44. Datiko DG, Yassin MA, Theobald SJ, Blok L, Suvanand S, Creswell J, Cuevas LE.
707 Health extension workers improve tuberculosis case finding and treatment outcome in

For peer review only

For peer review only - http://bmjopen.bmj.com/site/about/guidelines.xhtml

Ethiopia: a large-scale implementation study. BMJ Glob Health. 2017;2:e000390. doi: 10.1136/ bmjgh-2017-000390.

45. Welshman J. Compulsion, localism, and pragmatism: the micro-politics of tuberculosis screening in the United Kingdom, 1950-1965. Soc Hist Med. 2006;19:2:295-312. doi: 10.1093/shm/hkl002.

46. Welshman J, Bashford A. Tuberculosis, migration, and medical examination: lessons from history. J Epidemiol Community Health. 2006;60:282-284. doi: 10.1136/jech.2005.038604.

47. Mor Z, Lerman Y, Leventhal A. Pre-immigration screening process and pulmonary tuberculosis among Ethiopian migrants in Israel. Eur Respir J. 2008;32:413-418. doi: 10.1183/09031936.00145907.

48. Abubakar I, Stagg H, Cohen T, Mangtani P, Rodrigues LC, Pimpin L, Watson JM, Squire SB, Zumla A. Controversies and unresolved issues in tuberculosis prevention and control: a low-burden-country perspective. J Infect Dis. 2012;205:S293-300. doi: 10.1093/infdis/jir886.

49. Dierberg KL, Dorjee K, Salvo F, Cronin WA, Boddy J, Cirillo D, Sadutshang T, et al. Improved detection of tuberculosis and multidrug-resistant tuberculosis among Tibetan refugees, India. Emerg Infect Dis. 2016;22:3. doi: 10.3201/eid2203.140732.

50. Morishita F, Yadav R-P, Eang MT, Saint S, Nishikiori N. Mitigating Financial Burden of Tuberculosis through Active Case Finding Targeting Household and Neighbourhood Contacts in Cambodia. PLoS One. 2016;11:9:e0162796. doi: 10.1371/ journal.pone.0162796.

51. Harper I, Fryatt R, White A. Tuberculosis case finding in remote mountainous areas – are microscopy camps of any value? Experience from Nepal. Tuber Lung Dis. 1996;77:384-388.

For peer review only - http://bmjopen.bmj.com/site/about/guidelines.xhtml

52. Southern A, Premaratne N, English M, Balazs J, O'Sullivan D. Tuberculosis among homeless people in London: an effective model of screening and treatment. Int J Tuber Lung Dis. 1999;3:11:1001-1008.

53. O'Hara NN, Roy L, O'Hara LM, Spiegel JM, Lynd LD, FitzGerald JM, et al. Healthcare Worker Preferences for Active Tuberculosis Case Finding Programs in South Africa: A Best-Worst Scaling Choice Experiment. PLoS One. 2015;10:7: e0133304. doi: 10.1371/journal.pone.0133304.

54. Brewin P, Jones A, Kelly M, McDonald M, Beasley E, Sturdy P, et al. Is screening for tuberculosis acceptable to immigrants? A qualitative study. J Public Health. 2006;28:3:253-260. doi: 10.1093/pubmed/fdl031.

55. Chemtob D, Leventhal A, Weiler-Ravell D. Screening and management of tuberculosis in immigrants: the challenge beyond professional competence. Int J Tuberc Lung Dis. 2003;7:10:959-966.

56. MacPherson DW, Gushulak BD. Balancing prevention and screening among international migrants with tuberculosis: Population mobility as the major epidemiological influence in low-incidence nations. Public Health. 2006;120:712-723. doi: 10.1016/j.puhe.2006.05.002.

57. Lönnroth K, Migliori GB, Abubakar I, D'Ambrosio L, de Vries G, Diel R, Douglas P, et al. Towards tuberculosis elimination: an action framework for low-incidence countries. Eur Respir J. 2015;45:928-952. doi: 10.1183/09031936.00214014.

58. Mayo K, White S, Oates SK, Franklin F. Community collaboration: prevention and control of tuberculosis in a homeless shelter. Public Health Nurs. 1997;13:2:120-127.

59. Tardin A, Dominicé Dao M, Ninet B, Janssens J-P. Tuberculosis cluster in an immigrant community: case identification issues and a transcultural perspective. Trop Med Int Health. 2009;14:9:995-1002. doi: 10.1111/j.1365-3156.2009.02325.x.

For peer review only

60. World Health Organization. Use of high burden country lists for TB by WHO in the post-2015 era. Switzerland, Geneva: World Health Organization; 2015.

61. World Health Organization. Towards TB elimination: an action framework for low-incidence countries. WHO/ HTM/TB/2014.13. Switzerland, Geneva: World Health Organization; 2014.

62. Miller CR, Mitchell EMH, Nishikiori N, Zwerling A, Lönnroth K. A web-based tool for prioritizing risk groups and selecting algorithms for screening for active tuberculosis. (Submitted)

63. Stop TB Partnership. http://www.stoptb.org/ (2019). Accessed 25 Mar 2019.

64. Khan K, Hirji MM, Miniota J, Hu W, Wang J, Gardam M, Rawal S, et al. Domestic impact of tuberculosis screening among new immigrants to Ontario, Canada. CMAJ. 2015;187:16:e473-E481.2015. doi: 10.1503/cmaj.150317.

65. Adejumo AO, Azuogu B, Okorie O, Lawal OM, Onazi OJ, Gidado M, Daniel M, Okeibunor JC, Klinkenberg E, Mitchell EMH. Community referral for presumptive TB in Nigeria: a comparison of four models of active case finding. BMC Public Health. 2016;16:177. doi: 10.1186/s12889-016-2769-7.

66. Ayles H, Muyoyeta M, Du Toit E, Schaap A, Floyd S, Simwinga M, Shanaube K, et al. Effect of household and community interventions on the burden of tuberculosis in southern Africa: the ZAMSTAR community-randomised trial. Lancet. 2013;382:1183-94. doi: 10.1016/ S0140-6736(13)61131-9.

67. Azman AS, Golub JE, Dowdy DW. How much is tuberculosis screening worth? Estimating the value of active case finding for tuberculosis in South Africa, China, and India. BMC Med. 2014;12:216. doi: 10.1186/s12916-014-0216-0.

68. Bell TR, Molinari NAM, Blumensaadt S, Selent MU, Arbisi M, Shah N, Christiansen D, et al. Impact of port of entry referrals on initiation of follow-up evaluations for

For peer review only

immigrants with suspected tuberculosis: Illinois. J Immigr Minor Health. 2013;15:673-679. doi: 10.1007/s10903-013-9779-7.

69. Whitehead M, Dahlgren G. Concepts and principles for tackling social inequities in health: Levelling up Part 1. World Health Organization Collaborating Centre for Policy Research on Social Determinants of Health, University of Liverpool. Copenhagen: World Health Organization Regional Office for Europe; 2006.

70. Wright CW. An opinion survey of tuberculosis case-finding procedures. S Afr Med J. 1979;56:6:224-8.

71. Layton MC, Cantwell MF, Dorsinville GJ, Valway SE, Onorato IM, Frieden TR. Tuberculosis screening among homeless persons with AIDS living in single-room-occupancy hotels. Am J Public Health. 1995;85:11:1555-1559.

72. Erkens C, Slump E, Kamphorst M, Keizer S, van Gerven PJHJ, Bwire R, Berkel M, et al. Coverage and yield of entry and follow-up screening for tuberculosis among new immigrants. Eur Respir J. 2008;32:153-161. doi: 10.1183/09031936.00137907.

73. Hogan H, Coker R, Gordon A, Meltzer M, Pickles H. Screening of new entrants for tuberculosis: responses to port notifications. J Public Health. 2005;27:2:192-195. doi: 10.1093/pubmed/fdi019.

74. Klinkenberg E, Manissero D, Semenza JC, Verver S. Migrant tuberculosis screening in the EU/EEA: yield, coverage and limitations. Eur Respir J. 2009;34:1180-1189. doi: 10.1183/09031936.00038009.

75. Tan L, Altman RD, Nielsen NH. Screening nonimmigrant visitors to the United States for Tuberculosis. Arch Intern Med. 2001;161:334-340. doi: 10.1080/07370016.2013.778723.

76. Akkerman OW, de Lange WCM, Schölvinck EH, Wolters B, Aartsma Y, van der Werf TS, van Hest R. Implementing tuberculosis entry screening for asylum seekers:

For peer review only - http://bmjopen.bmj.com/site/about/guidelines.xhtml

the Groningen experience. Eur Respir J. 2016;48:261-264. doi: 10.1183/13993003.00112-2016.

77. Alvarez GG, Gushulak B, Rumman KB, Altpeter E, Chemtob D, Douglas P, et al. A comparative examination of tuberculosis immigrant medical screening programs from selected countries with high immigration and low tuberculosis incidence rates. BMC Infect Dis. 2011;11:3. doi: 10.1186/1471-2334-11-3.

78. Bloss E, Newbill K, Peto H, Rice MJ, Ainsowrth G, Travnicek R, Holcombe M, et al. Challenges and opportunities in a tuberculosis outbreak investigation in Southern Mississippi, 2005-2007. South Med J. 2011;104:11. doi: 10.1097/SMJ.0b0.3e318232679e.

79. Corbett EL, Bandason T, Duong T, Dauya E, Makamure B, Churchyard GJ, Williams BG, et al. Comparison of two active case-finding strategies for community-based diagnosis of symptomatic smear-positive tuberculosis and control of infectious tuberculosis in Harare, Zimbabwe (DETECTB): a cluster-randomised trial. Lancet. 2010;376:1244-53. doi: 10.1016/S0140- 6736(10)61425-0.

80. Mhimbira FA, Cuevas LE, Dacombe R,Mkopi A, Sinclair D. Interventions to increase tuberculosis case detection at primary healthcare or community-level services. Cochrane Database Syst Rev. 2017;11:CD011432. doi: 10.1002/14651858.CD011432.pub2.

81. Fox GJ, Nhung NV, Sy DN, Hoa NLP, Anh LTN, Anh NT, Hoa NB, et al. Household-contact investigation for detection of tuberculosis in Vietnam. N Engl J Med. 2018;378:221-9. doi: 10.1056/NEJMoa1700209.

82. Fox GJ, Barry SE, Britton WJ, Marks GB. Contact investigation for tuberculosis: a systematic review and meta-analysis. Eur Respir J. 2013;41:140-156. doi: 10.1183/09031936.00070812.

For peer review only - http://bmjopen.bmj.com/site/about/guidelines.xhtml

83. Honarvar B, Odoomi N, Rezaei A, Haghighi HB, Karimi M, Hosseini A, Mazarei S, et al. Pulmonary tuberculosis in migratory nomadic populations: the missing link in Iran's National Tuberculosis Programme. Int J Tuberc Lung Dis. 2014;18:3:272-276. doi: 10.5588/ijtld.13.0650.

84. Kranzer K, Houben RMG, Glynn JR, Bekker L-G, Wood R, Lawn SD. Yield of HIV-associated tuberculosis during intensified case finding in resource-limited settings: a systematic review and meta-analysis. Lancet Infect Dis. 2010;10:93-102. doi: 10.1016/S1473-3099(09)70326-3.

85. Lönnroth K, Mor Z, Erkens C, Bruchfeld J, Nathavitharana RR, van der Werf MJ, Lange C. Tuberculosis in migrants in low-incidence countries: epidemiology and intervention entry points. Int J Tuberc Lung Dis. 2017;21:6:624-636. doi: 10.5588/ijtld.16.0845.

86. Mallick G, Shewade HD, Agrawal TK, Kumar AMV, Chadha SS. Enhanced tuberculosis case finding through advocacy and sensitisation meetings in prisons of Central India. Public Health Action. 2017;7:1:67-70. doi: 10.5588/pha.16.0109.

87. Mulder C, Erkens CGM, Kouw PM, Huisman EM, Meijer-Veldman W, Borgdorff MW, van Leth F. Missed opportunities in tuberculosis control in The Netherlands due to prioritization of contact investigations. Eur J Public Health. 2011;22:2:177-182. doi: 10.1093/eurpub/ckr017.

88. Mwansa-Kambafwile J, McCarthy K, Gharbaharan V, Venter FWD, Maitshotlo B, Black A. Tuberculosis Case Finding: Evaluation of a Paper Slip Method to Trace Contacts. PLoS One. 2013;89:e75757. doi: 10.1371/journal.pone.0075757.

89. Ospina JE, Orcau A, Millet J-P, Sánchez F, Casals M, Caylà JA. Community health workers improve contact tracing among immigrants with tuberculosis in Barcelonaa. BMC Public Health. 2012;12:158. doi: 10.1186/1471-2458-12-158.

For peer review only - http://bmjopen.bmj.com/site/about/guidelines.xhtml

858 90. Smit GSA, Apers L, Arrazola de Onate W, Beutels P, Dorny P, Forier A-M, et al. Cost-effectiveness of screening for active cases of tuberculosis in Flanders, Belgium. Bull World Health Organ. 2017;95:1:27-35. doi: 10.2471/BLT.16.169383.

861 91. Szkwarko D, Mercer T, Kimani S, Braitstein P, Buziba N, Carter EJ. Implementing intensified tuberculosis case-finding among street-connected youth and young adults in Kenya. Public Health Action. 2016;6:2. doi: 10.1371/journal.pone.0182185.

864 92. Shrivastava SR, Shrivastava PS. Tuberculosis: Active Case Finding Survey in an Urban area of India, in 2012s. J Res Health Sci. 2013;13:1:19-23.

867 **Tables**

868 *Table 1 Characteristics of the articles included in the scoping review (n=73)*

**Study characteristics**	**References**	**Number (%)**
**Year of publication**		
1979	70	1 (1%)
1995-1999	51,52,58,71	4 (5%)
2000-2009	15,22,24,25,30,35,45,46,47,55,56,59,72,73,74,75	16 (22%)
2010-2018	13,14,16,17,18,19,23,26,27,28,29,31,32,33,34,36,37, 38,39,41,43,44,48,49,50,53,54,57,64,65,66,67,68,76, 77,78,79,80,81,82,83,84,85,86,87,88,89,90,91	49 (67%)
**Region**		
Africa	18,23,28,32,37,38,43,44,53,65,66,67,70,79,88,91	16 (22%)
Americas (North America)	24,25,39,42,58,64,68,71,75,78	10 (14%)
Eastern Mediterranean	83	1 (1%)
Europe	30,33,40,45,46,47,52,54,55,59,72,73,74,76,87,89,90	17 (23%)

South-East Asia	49,51,67,86,92	5 (7%)
Western Pacific	13,14,34,35,36,50,67,81,82	9 (12%)
Global perspective	17,22,29,41,80,82,84	7 (10%)
Other*	15,31,48,77	4 (5%)
**Study design**		
Quantitative	13,14,19,31,32,35,36,37,43,47,48,49,50,51,53,59,64, 66,67,68,70,71,72,73,79,81,83,89,90,92	30 (41%)
Review	15,16,17,18,26,29,42,45,48,56,74,75,80,82,84,85	16 (22%)
Qualitative	23,24,28,33,40,54,55,65,77	9 (12%)
Descriptive	30,39,77,87,91	5 (7%)
Case study	27,58,78	3 (4%)
Mixed methods	34,38	2 (3%)
Other**	41,46,52,76	4 (5%)
**Target group**		
Contacts	13,19,22,23,25,28,29,38,39,43,50,66,80,81,82,87,88,90	18 (25%)
Migrants	18,30,33,40,42,45,46,47,48,54,55,56,59,64,68,72,73, 74,77,79,83,85,89	23 (32%)
Community	15,27,31,32,35,36,44,51,65,67	10 (14%)
High risk groups	17,26,41,57,70	5 (7%)
Homeless	24,52,58,71	4 (5%)
Asylum seekers or refugees	49,76	2 (3%)
People living with HIV	16,84	2 (3%)
Urban poor	14,34	2 (3%)
Other***	37,75,78,86,91,92	6 (8%)

869 **High HIV prevalence countries [15], high TB incidence countries [31], low-burden countries [48,77]*

870 ***Letter to editor [76], observational study [52], perspective [41], editorial [46]*

For peer review only - http://bmjopen.bmj.com/site/about/guidelines.xhtml

****Population affected by TB outbreak [78], pastoralists [37], health care workers [53], prisoners [86], people living in slums [92], street connected youth and young adults [91], non-immigrant visitors [75]*

Table 2 Barriers and facilitators for active case-finding policy implementation (n=43)

**Reported factors**	**References**	**Number (%)**
Health system level		
Limited financial resources	14,22,23,27,29,34,36,39,40,45,47,51,55, 70,84	15 (35%)
Existing systems and structures	17,22,28,29,33,40,43,52,55,74,84	11 (26%)
Availability of diagnostic tests and services	14,17,29,37,42,48,49,70	8 (19%)
Staff experience, expertise, motivation	3,15,21,25,27,28,29,34,36,37,40,45,54,58	14 (33%)
Over-burdening staff	14,22,27,34,37,51,75	7 (16%)
Using person-centred approaches	23,24,28,29,40,42,52,53	8 (19%)
Collaboration and integration	14,16,28,34,40,42,76	7 (16%)
Health education	36,37,38,40,42	5 (12%)
Health information and research system level		
Data, systems, supervision	17,25,29,30,31,38,47	7 (16%)
Individual and community level		
Stigma and discrimination	19,23,24,25,26,28,29,30,33,35,40,49,58, 59,83,88	16 (37%)
Individual characteristics	19,24,34,35,40,42,51,53,58,70,83,89	12 (28%)

For peer review only

Socio-cultural factors	25,26,29,30,37,40,42,45,46,55,56,59,89,83	14 (33%)
Fear	24,25,33,34,40,42,52,54,55,56	10 (23%)
Mistrust	25,28,37,40,45,46,51,52,57,78	10 (23%)
Knowledge and awareness	19,24,25,27,28,40,52,58	8 (19%)

For peer review only

Fig 1. PRISMA flow diagram

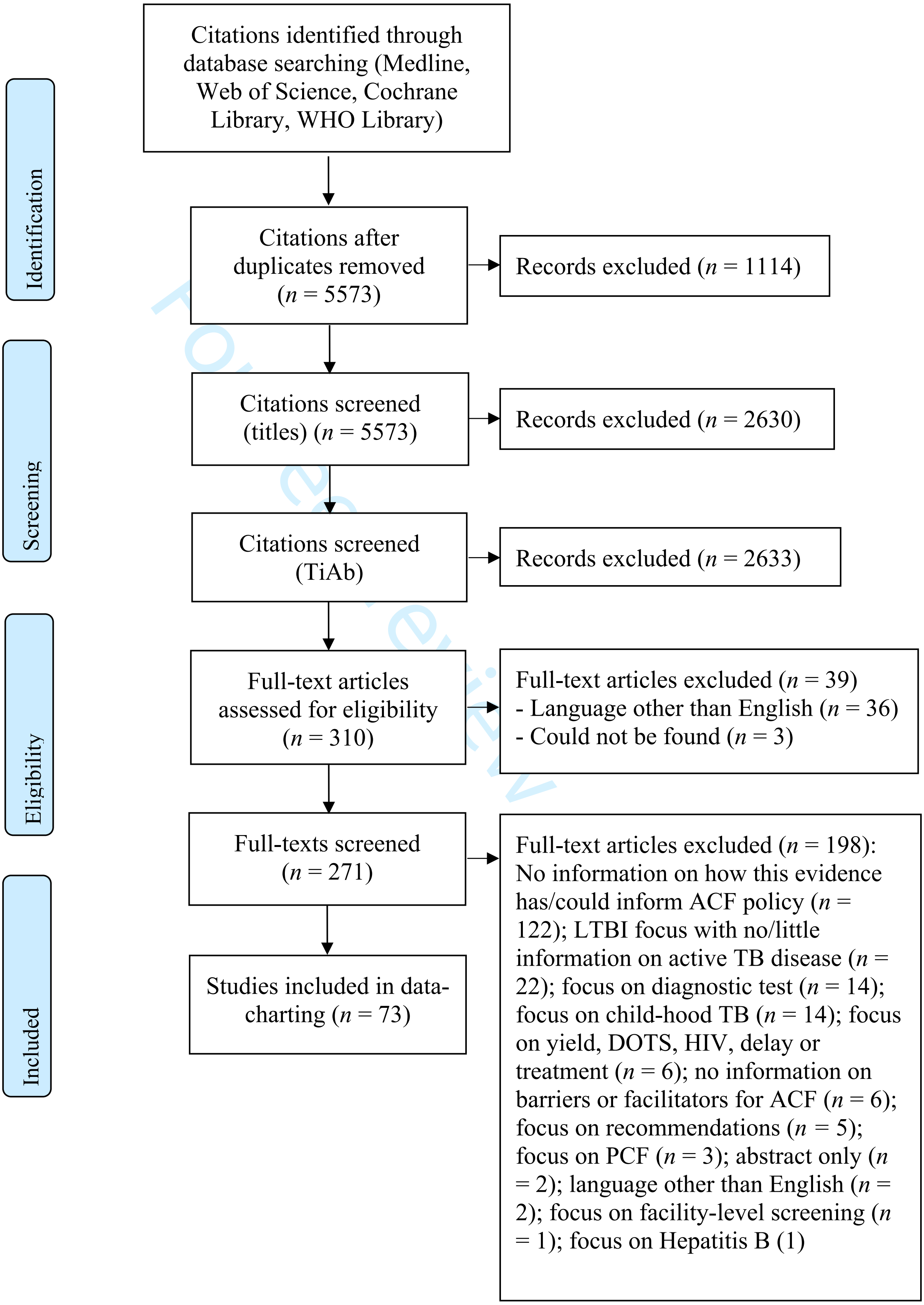

Abbreviations mentioned in Fig. 1: TiAb = Title and abstract; LTBI = Latent Tuberculosis Infection; DOTS = Directly Observed Treatment, Short Course; HIV = Human Immunodeficiency Virus; PCF = Passive Case-Finding

For peer review only

1 Additional file 1: Preferred Reporting Items for Systematic reviews and Meta-Analyses
2 extension for Scoping Reviews (PRISMA-ScR) Checklist

3

**Section**	**Item**	**PRISMA-ScR Checklist Item**	**Check** (Yes/No)
**Title**	1	Identify the report as a scoping review	Yes
**Abstract**<br>Structured summary	2	Provide a structured summary that includes (as applicable) background, objectives, eligibility criteria, sources of evidence, charting methods, results, and conclusions that relate to the review questions and objectives	Yes
**Introduction**<br>Rationale	3	Describe the rationale for the review in the context of what is already known. Explain why the review questions/objectives lend themselves to a scoping review approach	Yes
Objectives	4	Provide an explicit statement of the questions and objectives being addressed with reference to their key elements (e.g., population or participants, concepts, and context) or other relevant key elements used to conceptualize the review questions and/or objectives.	Yes

For peer review only - http://bmjopen.bmj.com/site/about/guidelines.xhtml

**Methods**<br>Protocol and registration	5	Indicate whether a review protocol exists; state if and where it can be accessed (e.g., a Web address); and if available, provide registration information, including the registration number.	Yes
Eligibility criteria	6	Specify characteristics of the sources of evidence used as eligibility criteria (e.g., years considered, language, and publication status), and provide a rationale.	Yes
Information sources*	7	Describe all information sources in the search (e.g., databases with dates of coverage and contact with authors to identify additional sources), as well as the date the most recent search was executed.	Yes
Search	8	Present the full electronic search strategy for at least 1 database, including any limits used, such that it could be repeated.	Yes
Selection of sources of evidence†	9	State the process for selecting sources of evidence (i.e., screening and eligibility) included in the scoping review	Yes
Data charting process‡	10	Describe the methods of charting data from the included sources of evidence (e.g., calibrated forms or forms that have been tested by the team before their use,	Yes

For peer review only - http://bmjopen.bmj.com/site/about/guidelines.xhtml

For peer review only

		and whether data charting was done independently or in duplicate) and any processes for obtaining and confirming data from investigators.	
Data items	11	List and define all variables for which data were sought and any assumptions and simplifications made.	Yes
Critical appraisal of individual sources of evidence§	12	If done, provide a rationale for conducting a critical appraisal of included sources of evidence; describe the methods used and how this information was used in any data synthesis (if appropriate).	No
Summary measures	13	Not applicable for scoping reviews	N/A
Synthesis of results	14	Describe the methods of handling and summarizing the data that were charted.	Yes
Risk of bias across studies	15	Not applicable for scoping reviews	N/A
Additional analyses	16	Not applicable for scoping reviews	N/A
**Results** Selection of sources of evidence	17	Give numbers of sources of evidence screened, assessed for eligibility, and included in the review, with reasons for exclusions at each stage, ideally using a flow diagram.	Yes

For peer review only - http://bmjopen.bmj.com/site/about/guidelines.xhtml

Characteristics of sources of evidence	18	For each source of evidence, present characteristics for which data were charted and provide the citations.	Yes
Critical appraisal within sources of evidence	19	If done, present data on critical appraisal of included sources of evidence (see item 12).	No
Results of individual sources of evidence	20	For each included source of evidence, present the relevant data that were charted that relate to the review questions and objectives.	No
Synthesis of results	21	Summarize and/or present the charting results as they relate to the review questions and objectives.	Yes
Risk of bias across studies	22	Not applicable for scoping reviews.	N/A
Additional analyses	23	Not applicable for scoping reviews.	N/A
**Discussion** Summary of evidence	24	Summarize the main results (including an overview of concepts, themes, and types of evidence available), link to the review questions and objectives, and consider the relevance to key groups.	Yes
Limitations	25	Discuss the limitations of the scoping review process.	Yes
Conclusions	26	Provide a general interpretation of the results with respect to the review	Yes

For peer review only - http://bmjopen.bmj.com/site/about/guidelines.xhtml

		questions and objectives, as well as potential implications and/or next steps.	
**Funding**	27	Describe sources of funding for the included sources of evidence	No
		Describe sources of funding for the scoping review. Describe the role of the funders of the scoping review.	Yes

JBI = Joanna Briggs Institute; PRISMA-ScR = Preferred Reporting Items for Systematic reviews and Meta-Analyses extension for Scoping Reviews.

* Where sources of evidence (see second footnote) are compiled from, such as bibliographic databases, social media platforms, and Web sites.

† A more inclusive/heterogeneous term used to account for the different types of evidence or data sources (e.g., quantitative and/or qualitative research, expert opinion, and policy documents) that may be eligible in a scoping review as opposed to only studies. This is not to be confused with information sources (see first footnote).

‡ The frameworks by Arksey and O'Malley (6) and Levac and colleagues (7) and the JBI guidance (4, 5) refer to the process of data extraction in a scoping review as data charting.

§ The process of systematically examining research evidence to assess its validity, results, and relevance before using it to inform a decision. This term is used for items 12 and 19 instead of “risk of bias” (which is more applicable to systematic reviews of interventions) to include and acknowledge the various sources of evidence that may be used in a scoping review (e.g., quantitative and/or qualitative research, expert opinion, and policy documents)

For peer review only

For peer review only - http://bmjopen.bmj.com/site/about/guidelines.xhtml

Adapted from: Tricco A, Lillie E, Zarin W, O'Brien KK, Colquhoun H, Levac D, et al. PRISMA Extension for Scoping Reviews (PRISMA-ScR): Checklist and Explanation. Ann Intern Med. 2018;169:467-473. doi: 10.7326/M18-0850.

For peer review only

For peer review only - http://bmjopen.bmj.com/site/about/guidelines.xhtml

Additional file 2: Literature search strategy for MEDLINE

We implemented the search strategy on 24 January 2018. The search yielded 4640 results.

1 exp Tuberculosis/ or exp Mycobacterium tuberculosis/

2 (tuberculosis or tb or pulmonary consumption or consumption, pulmonary or pulmonary phthisis or tuberculoses).ti,ab.

3 1 or 2

4 exp Mass screening/ or exp Mass chest x-ray/ or exp Contact tracing/ or exp Health surveys/ or exp Cross-sectional studies/ or exp Epidemiologic studies/ or exp Systematic review/

5 (Mass chest x ray$ or screen$ or mass screening$ or population screening$ or survey$ or screening survey$ or household survey or health survey$ or cross-sectional or detect$ or active case or case detect$ or case finding or tuberculosis case finding or active case finding or contact tracing or contact examination or contact screening or contact investigation or employment testing or intensified case finding or prevalence stud$ or prevalen* or inciden* or algorithm or undiagnosed or checking or pre-entry or passive or TB suspect$ or notification or notified).ti,ab.

6 4 or 5

7 (view$ or barrier$ or block$ or obstacle$ or hinder$ or constrain$ or facilitat$ or enabl$ or benefit$ or opportun$ or attitude$ or opinion$ or belief$ or perceiv$ or perception$ or aware$ or personal view$ or motivat$ or reason$ or incentiv$ or cost$ or cost-effective$ or cost analys$ or cost comparison or cost-minimization or cost measure$ or affordab$ or resource$ or resource allocation or resource$ or efficiency).ti,ab.

For peer review only - http://bmjopen.bmj.com/site/about/guidelines.xhtml

8 exp Attitude/ or exp Motivation/ or exp Cost/ or exp Cost analysis/ or exp Resource allocation/

9 7 or 8

10 exp Policy Making/ or exp Health Plan Implementation/ or exp Health Priorities/

11 (policy mak$ or policy develop$ or policy analys$ or health polic$ or policy implement$ or health plan implement$ or priorit$ or health priorit$).ti,ab.

12 10 or 11

13 9 or 12

14 (((((((((((((((((hard$ adj2 reach) or hard$) adj2 locate) or hard$) adj2 find) or hard$) adj2 treat) or difficult) adj2 locate) or Difficult) adj2 engage) or social$ exclu$ or social inequalit$ or difficult$) adj2 reach) or difficult$) adj2 find) or difficult$) adj2 treat).ti,ab.

15 ((geograph$ or transport$ or physical) and barrier$).ti,ab.

16 ((low$ or poor$ or negative) and (quality adj2 life)).ti,ab.

17 ((vulnerable or disadvantaged or at risk or high risk or low socioeconomic status or neglect$ or marginal$ or forgotten or non-associative or unengaged or hidden or excluded or transient or inaccessible or underserved or stigma$ or inequitable) and (people or population$ or communit$ or neighbourhood$1 or neighborhood$1 or group$ or area$1 or demograph$ or patient$ or social$)).ti,ab.

18 vulnerable populations/

19 17 or 18

For peer review only - http://bmjopen.bmj.com/site/about/guidelines.xhtml

20 poverty area/

21 (refuser$1 or non-user$1 or discriminat$ or shame or prejud$ or racism or racial discriminat$).ti,ab.

22 social support/ or *social conditions/ or stigma/ or Social Isolation/ or *quality of life/ or Prejudice/ or Socioeconomic Factors/

23 (immobile or (disabled and (house bound or home bound)) or ((house or home) and bound)).ti,ab.

24 Homebound Persons/

25 23 or 24

26 ((hous$ and (quality or damp$ or standard$ or afford$ or condition$ or dilapidat$)) or ((emergency or temporary or inadequate or poor$ or overcrowd$ or over-crowd$ or over-subscribed) and (hous$ or accommodation or shelter$ or hostel$ or dwelling$))).ti,ab.

27 housing/st

28 26 or 27

29 (rough sleep$ or runaway$1 or ((homeless$ or street or Destitut$) and (population or person$1 or people or group$ or individual$1 or shelter$ or hostel$ or accommodation$1))).ti,ab.

30 exp homeless persons/

31 29 or 30

32 ((drug$ or substance) and (illegal or misus$ or abuse or intravenous or IV or problem use$ or illicit use$ or addict$ or dependen$ or dependant or delinquency)).ti,ab.

For peer review only

For peer review only - http://bmjopen.bmj.com/site/about/guidelines.xhtml

33 *Substance-Related Disorders/ or Drug users/

34 32 or 33

35 ((alcohol$ and (misus$ or abuse or problem$ use$ or problem drink$ or illicit use$ or addict$ or dependen$ or dependant or delinquency)) or alcoholic$1).ti,ab.

36 *Alcohol- Related Disorders/ or Alcoholics/

37 35 or 36

38 (prostitution or sex work$ or transactional sex$ or prostitute$1).ti,ab.

39 Prostitution/

40 38 or 39

41 (poverty or deprivation or financial hardship$).ti,ab.

42 ((low-income or low income or low pay or low paid or poor or deprived or debt$ or arrear$) and (people or person$1 or population$1 or communit$ or group$ or social group$ or neighbourhood$1 or neighborhood$1 or famil$)).ti,ab.

43 poverty/

44 (low$ and social class$).ti,ab.

45 (traveller$1 or gypsies or gypsy or Romany or roma).ti,ab.

46 gypsies/

47 45 or 46

48 (mental$ and (health or ill or illness)).ti,ab.

For peer review only

49 *mental health/ or Mentally Ill Persons/

50 48 or 49

51 ((((health care worker$1 or health care) adj2 service provi$) or health-care) adj2 provi$).ti,ab.

52 (outreach adj2 worker$1).ti,ab.

53 Community health aides/

54 52 or 53

55 (support adj2 worker$1).ti,ab.

56 (case adj2 worker$1).ti,ab.

57 (social adj2 worker$1).ti,ab.

58 social care professional$1.ti,ab.

59 ((social care adj2 service provi$) or (social-care adj2 provi$)).ti,ab.

60 (((language$ or communicat$) and (barrier$ or understand$ or strateg$ or proficien$)) or translat$ or interpret$ or (cultur$ and competen$)).ti,ab.

61 Communication Barriers/ or *Language/

62 60 or 61

63 (((immigrant$ or migrant$ or asylum or refugee$ or undocumented or foreign born or born) adj overseas) or (displaced and (people or person$1))).ti,ab.

For peer review only

64 ((((Emigration.mp. and Immigration/) or refugees/ or Transients.mp.) and migrants/) or Emigrants.mp.) and immigrants/ [mp=title, abstract, original title, name of substance word, subject heading word, keyword heading word, protocol supplementary concept word, rare disease supplementary concept word, unique identifier, synonyms]

65 63 or 64

66 exp Diabetes Mellitus/

67 diabet$.tw,ot.

68 (IDDM or NIDDM or MODY or T1DM or T2DM or T1D or T2D).tw,ot.

69 (non insulin$ depend$ or noninsulin$ depend$ or non insulin?depend$ or noninsulin?depend$).tw,ot.

70 (insulin$ depend$ or insulin?depend$).tw,ot.

71 or/66-70

72 14 or 15 or 16 or 19 or 20 or 21 or 22 or 25 or 28 or 31 or 34 or 37 or 40 or 41 or 42 or 43 or 44 or 47 or 50 or 51 or 54 or 55 or 56 or 57 or 58 or 59 or 62 or 65 or 71

73 3 and 6 and 13 and 72

74 limit 73 to yr="1968 -Current"

75 animals/ not humans/

76 74 not 75

For peer review only

For peer review only - http://bmjopen.bmj.com/site/about/guidelines.xhtml

Additional file 3: Abridged version of the data charting table

The abridged data charting table contains information on author, year of publication and study design (column 1), the country where the study was implemented and the target population (column 2), ACF antecedents (column 3), ACF components (column 4), factors influencing ACF policy implementation at the level of the health system (column 5) and the individual and community level (column 6).

	1	2	3	4	5	6
**ID**	**Author, year, design**	**Country, target population**	**ACF antecedents**	**ACF components**	**Factors influencing ACF policy implementation:** health system context	**Factors influencing ACF policy implementation:** individual and community context
1	Abubakar, 2012, review	Low-burden, migrants			Screening site characteristics; diagnostic test availability; molecular assay costs	
2	Adejumo, 2016, qualitative	Nigeria, community			CHW age, work experience, knowledge	
3	Akkerman, 2016, letter to editor	the Netherlands, asylum-seekers			High-volume CXR readers; cooperation between public health and clinical workers	

For peer review only - http://bmjopen.bmj.com/site/about/guidelines.xhtml

4	Alvarez*, 2011, descriptive	Low-burden, migrants	despite lack of evidence, ACF is major global health priority			
5	Ayakaka, 2017, qualitative	Uganda, contacts		Education; persuasion; enablement; incentivization	CHW travel costs, pay, skills; personalized, enabling services; staff shortages; fear of TB; patient communication; language barrier; difficulty locating households; lack of ACF guidelines	Fear of stigma; discrimination; among contacts: limited knowledge, mistrust, travel costs, avoidant behavior; privacy provided by home evaluation
6	Ayles, 2013, quantitative	South Africa, Zambia, contact			Linkage and retention in care	Empowerment to seek care early
7	Azman*, 2014, quantitative	China, India, South Africa, community				
8	Baxter, 2017, review	Worldwide, contacts			Using molecular epidemiological testing; ensuring adequate systems; local expertise and capability; communication; coordination; prompt	Stigma; language barrier; high mobility

For peer review only - http://bmjopen.bmj.com/site/about/guidelines.xhtml

					action; addressing fear; error; lack of resources	
9	Bell*, 2013, quantitative	USA, migrants				
10	Bloss, 2011, case study	USA, outbreak			Institutional knowledge about TB control; limited human resources	Community trust and rapport
11	Van den Bosch, 2000, descriptive	UK, migrants				Change of address; false address; language barrier; mistrust of authorities; fear of TB and stigma
12	Brewin, 2006, qualitative	UK, migrants			Integration of health and social services; waiting time	Feelings of reassurance, responsibility, anxiety linked to ACF; lack of public health information
13	Chemtob, 2003, qualitative	Israel, migrants			Adequate health facilities; funds for transportation; attitudes; dialogue with immigrants and providers in planning and implementation	Socio-cultural barriers; anti-immigrant sentiments; fear of deportation; no standard term for TB; empowerment to assume greater responsibility for health promotion

For peer review only - http://bmjopen.bmj.com/site/about/guidelines.xhtml

14	Cook, 2012, descriptive	Canada, aboriginal community			Foundation of routine ACF including interview skills, dedicated resources to undertake ACF, follow-up	Community buy-in
15	Corbett, 2013, review	Africa, PLHIV	Radiological screening widely implemented before 1970s	Target group: populations with high prevalence	Sensitivity of diagnostic tests; communication and interaction with the community; transportation	
16	Corbett, 2010, quantitative	Zimbabwe, congregate setting	ACF implemented in HIC since 1920s; ACF discouraged due to costs and weak treatment programmes			
17	Datiko, 2017, quantitative	Ethiopia, community		Linkage to laboratory services and treatment through a transport network		
18	de Vries, 2017, review	Worldwide, high-risk		Awareness; cultural sensitivity; access	Structural barriers; cultural and language barriers	Low perceived susceptibility; stigma and its social consequences

For peer review only - http://bmjopen.bmj.com/site/about/guidelines.xhtml

For peer review only

				to high-quality translation services; cross-cultural training of providers		
19	Dierberg, 2016, quantitative	India, refugees			Use of Xpert MTB/RIF; available time and human resources; lack of a laboratory technician; trained providers	High mobility; awareness; stigma
20	Eang, 2012, quantitative	Cambodia, contacts	Growing interest in ACF in LMIC			
21	Erkens*, 2008, quantitative	the Netherlands, migrants				
22	Mhimbira, 2017, review	Worldwide, contacts		Health promotion activities		
23	Fox, 2018, quantitative	Vietnam, contacts	ACF in high-prevalence settings remains limited			

For peer review only - http://bmjopen.bmj.com/site/about/guidelines.xhtml

24	Fox, 2013, review	Worldwide, contacts, congregate setting	Growing interest ACF in LMICs			
25	Fox, 2015, quantitative	Vietnam, contacts	WHO recommendations		Proximity of services	Discrimination; lack of knowledge; misconceptions; attitudes and (traditional) beliefs; difficulty remembering appointments
26	Getnet, 2017, quantitative	Ethiopia, pastoralists			Proximity of services; diagnostic algorithm and tools used; quality of diagnostic services; provider expertise; burden on CHWs; health education	Health seeking behavior; lack of confidence in CHWs; personal or cultural beliefs; gender norms
27	Godfrey-Faussett, 2003, review	Worldwide, high HIV prevalence setting	Movement towards decentralized diagnostic centers		Fear; stigma	
28	Golub, 2005, review	Worldwide, contacts	Mass radiography as ACF strategy in HICs before 1960s;		Workload and cost; method of case detection; training of laboratory technicians, clinicians and field staff;	

For peer review only - http://bmjopen.bmj.com/site/about/guidelines.xhtml

			exploration of new ACF strategies; recognized global urgency		appropriate bacteriological examination; laboratory network	
29	O'Hara, 2015, quantitative	South Africa, health care workers			Cost and confidentiality of services; waiting times	Sex, occupation, time since a previous TB test
30	Harper, 1996, quantitative	Nepal, remote community			Cost; lack of staff and funding; absenteeism; use of 'alternative' services; accessibility of services	Low confidence amongst users and general under-usage of services
31	Hinderaker, 2011, quantitative	Low-income, low-income community	Testing of different ACF approaches since 1960s	Social mobilization; education; communication; private sector engagement; health systems	Regular supervision; availability of district laboratory supervisor	

For peer review only

				strengthening; incentives		
32	Hogan, 2005, quantitative	UK, migrants			Service capacity; lack of national guidance; inconsistency in implementing ACF	
33	Honarvar, 2014, quantitative	Iran, migrants				Stigma; high mobility; poverty; low literacy; tribal behaviors; traditional beliefs; gender norms; language barrier; self-discrimination
34	Karki, 2017, quantitative	Papua New Guinea, community	Role of ACF uncertain since 1970s		Poor health services; lack of outreach services; limited funds at district level; cost; availability of staff and the payment of allowances; staff skills and experience; health education	
35	Kerrigan, 2017, qualitative	South Africa, contacts	Despite lack of evidence, ACF is major global health priority		Provider training, safety; incentives; lack of resources, logistics; involvement of culturally respected, valued	Stigma; preference of different screening locations; travel time and costs; gossip

					stakeholder/organization venues; community-based mobilization and events	
36	Khan*, 2015, quantitative	Canada, migrants				
37	Klinkenberg, 2009, review	EU, migrants			Access to healthcare for migrants; availability of a good follow-up system; integration with healthcare	
38	Kranzer, 2010, review	Worldwide, PLHIV	Mass radiography in Czechoslovakia 1961-72 showing decrease in prevalence	Target population; screening strategy; laboratory capacity; operational feasibility; costs	Feasibility and costs; laboratory capacity	
39	Kranzer, 2012, review	South Africa, deprived community				Waiting time; ability to produce a sputum sample; health status

For peer review only - http://bmjopen.bmj.com/site/about/guidelines.xhtml

For peer review only

40	Kulane, 2010, qualitative	Sweden, Somali community				Stigma; migration; migration policies; fear of deportation; misconceptions
41	Layton, 1995, review	USA, homeless			Incentives	
42	Li, 2017, case study	China, community			Heavy workload; inadequate providers; time and human resource constraints; provider education and competence	Acceptance; knowledge; awareness; health status
43	Lorent, 2015, mixed methods	Cambodia, urban poor		Health education; community involvement; peer support networks	Challenging working conditions; collaboration between TB workers and CHWs; CHW compensation, familiarity, knowledge of community, remuneration; provider motivation; financial support	Acceptance; cost, fear; embarrassment; preference of different screening locations; privacy concerns; involvement of CHWs; non-acceptance of diagnosis
44	Lorent, 2014, quantitative	Cambodia, urban poor		Flexible patient-centered treatment approach	Logistics; finances; heavy workload; CHW integration, network, recognition, remuneration; use of mobile phone,	

For peer review only - http://bmjopen.bmj.com/site/about/guidelines.xhtml

					collaboration with providers and laboratory technicians; infrastructure	
45	Lönnroth, 2013, review	Worldwide, high-risk	WHO recommendation against indiscriminate mass screening (1974)	Confidentiality; access to free, good quality services; provider training; minimization of initial loss to follow-up; enforced notification; engagement of public/private providers; resources	Available, high-quality services; NTP performance; health system capacity; public health law; existing platforms for outreach and health promotion activities, health and social services	.;
46	Lönnroth, 2017, review	Low incidence, migrants		Target groups; screening algorithms; timing; values; preferences;		

For peer review only - http://bmjopen.bmj.com/site/about/guidelines.xhtml

				competing health care needs; weak evidence base		
47	Lönnroth, 2015, descriptive	Low-incidence, high-risk				Trust
48	MacPherson, 2006, review	Low-incidence, migrants				Fear; language barrier; culture; social factors
49	Mallick*, 2017, descriptive	India, prisoners				
50	Mayo, 1996, case study	USA, homeless			ACF location; incentives; providers' frustration; involvement of clients	Stigma; substance use; poor social skills; health status; poor access to services and the necessities of life; unemployment; homelessness
51	Mor, 2008, quantitative	Israel, migrants		Unification of medical procedures	Operational cost; infrastructure	

For peer review only - http://bmjopen.bmj.com/site/about/guidelines.xhtml

52	Morishita, 2016, quantitative	Cambodia, contacts				Cost; insurance
53	Mulder, 2011, descriptive	the Netherlands, contacts			Limited health system capacity to keep track of immigrant index cases	
54	Mwansa-Kambafwile, 2013, quantitative	South Africa, contacts				Discomfort linked to home visits; confidentiality; stigma; discrimination
55	Ospina, 2012, quantitative	Spain, migrants		Involvement of CHWs	CHW involvement; language barrier	Birthplace; unknown district of residence; HIV infection; homelessness; sex; incarceration history; language and cultural barrier
56	Seedat, 2014, qualitative	UK, migrants		Community, proactive services; collaboration with community organizations;	Lack of capacity, funding, advocacy, psycho-social support services, awareness; link with community organizations; cultural insensitivity; time and distance to services	Stigma; culture; gender norms; faith; language barrier; misconceptions; fear of lack of confidentiality, cost, disease status; lack of awareness, knowledge, confidence

For peer review only - http://bmjopen.bmj.com/site/about/guidelines.xhtml

				combining diseases into health check-up; awareness raising		
57	Sekandi, 2015, quantitative	Uganda, contacts	ACF currently used in research settings		Lack of well-organized public health systems; staff shortage; limited resources for personnel time, administration and transportation	
58	Shrestha-Kuwahara, 2003, qualitative	USA, contacts			Lack of clear definitions; challenge of communicating the need to obtain personal information; limited knowledge on socio-economic issues among staff; training of, frustration among providers	Trust; understanding; knowledge; feeling moral obligations to family/friends confusion; fear of alienation, abandonment, stigma, loss of employment or housing; negative perception of interview process; misconceptions; language and cultural barriers

For peer review only - http://bmjopen.bmj.com/site/about/guidelines.xhtml

59	Shrivastava, 2013, quantitative	India, slums	Periodic ACF remains an integral part of TB control in high-risk groups	Involvement of CHW		
60	Smit*, 2017, quantitative	Belgium, high-risk, contacts				
61	Southern, 1999, observational	UK, homeless		Immediate examination of CXR; fast-track referral system	Incentives; non-judgmental, flexible, supportive approach	Lack of interest; fear of the outcome; waiting times
62	Suthar, 2016, perspective	Worldwide, high-risk	2030 goals important for inspiring and persuading communities to collaborate and step up TB response			
63	Swigart, 2004, qualitative	USA, homeless				Health status; knowledge about TB; costs; fear; disinterest

For peer review only - http://bmjopen.bmj.com/site/about/guidelines.xhtml

64	Szkwarko, 2016, descriptive	Kenya, street-connected youth and young adults			Trust; difficulties obtaining morning sputum; underrepresentation of women	
65	Tan, 2001, review	USA, nonimmigrant visitors			Funding and logistical difficulties; technical issues; overburdening providers	
66	Tankimovich, 2013, review	USA, homeless, migrants		Coordination and collaboration of programmes	Incentives; accessible, affordable facilities and technology; cultural knowledge; coordination between governments, academic and private institutions	Lack of knowledge; misconceptions; contact difficulties; co-morbidities; cultural values
67	Tardin, 2009, quantitative	Switzerland, migrants				Stigma; different communication styles and health beliefs; language barrier; acceptance of diagnosis; respect towards authority; religious value; tight community network

For peer review only - http://bmjopen.bmj.com/site/about/guidelines.xhtml

68	Tupasi, 2000, quantitative	Philippines, community			Services free and conveniently located	Symptoms; fear of stigma
69	Welshman, 2006, review	UK, migrants			Dissatisfaction with work; disjunction between guidelines and practice; cost; logistics; administration; language barrier; shortage of radiologists/ radiographers	Changes of address; language barrier; mistrust of authorities
70	Welshman, 2006, editorial	UK, migrants			Logistics; administration; language barrier; shortages of radiologists/radiographers	Changes of address; language barrier; mistrust of authorities
71	Wright, 1979, quantitative	South Africa, high-risk			Limited and inadequate facilities for the examination of sputum; cost	"Degree of sophistication of the people involved"
72	Yassin, 2013, mixed methods	Ethiopia, contacts		Capacity strengthening; advocacy; communication; social mobilization; transport;	Providers' commitment; CHWs' job satisfaction; laboratory technicians' perceived benefit of ACF; burden on laboratory; incentives; benefit from new equipment; quality of smears prepared by CHWs	

For peer review only - http://bmjopen.bmj.com/site/about/guidelines.xhtml

				treatment; CHW training, supervision, support		
73	Zenner, 2017, review	Worldwide, migrants	WHO End TB strategy; WHO against indiscriminate mass screening (1974)		Type of programme; target group; voluntary vs mandatory ACF	

* The article contains important information on additional influencing factors beyond the ones displayed in this table. Due to space limitations, we did not include them here.

For peer review only

BMJ Open

Factors influencing active tuberculosis case finding policy development and implementation: a scoping review

Journal:	*BMJ Open*
Manuscript ID	bmjopen-2019-031284.R1
Article Type:	Original research
Date Submitted by the Author:	14-Aug-2019

Complete List of Authors:	Biermann, Olivia; Karolinska Institutet, Public Health Sciences<br>Lönnroth, Knut; Karolinska Institutet, Public Health Sciences<br>Caws, Maxine; Liverpool School of Tropical Medicine, Department of Clinical Sciences; Birat Nepal Medical Trust<br>Viney, Kerri ; Karolinska Institutet, Public Health Sciences; Australian National University, Research School of Population Health, College of Health and Medicine

**Primary Subject Heading**:	Public health
Secondary Subject Heading:	Global health, Health policy
Keywords:	Tuberculosis < INFECTIOUS DISEASES, active case-finding, Health policy < HEALTH SERVICES ADMINISTRATION & MANAGEMENT, policy development, policy implementation, PREVENTIVE MEDICINE

SCHOLARONE™ Manuscripts

Factors influencing active tuberculosis case finding policy development and implementation: a scoping review

Olivia Biermann[1]* (olivia.biermann@ki.se)

Knut Lönnroth[1] (knut.lonnroth@ki.se)

Maxine Caws[2 3] (maxine.caws@lstmed.ac.uk)

Kerri Viney[1 4] (kerri.viney@anu.edu.au)

1 Department of Public Health Sciences, Karolinska Institutet, Tomtebodavägen 18a, 17177 Stockholm, Sweden

2 Department of Clinical Sciences, Liverpool School of Tropical Medicine, Pembroke Pl, Liverpool L3 5QA, United Kingdom

3 Birat Nepal Medical Trust, Lazimpat, Kathmandu, Nepal

4 Research School of Population Health, College of Health and Medicine, Australian National University, Canberra ACT 2600, Australia

*Corresponding author

For peer review only

Abstract (299 words)

Background

The World Health Organization (WHO) emphasizes the importance of active case-finding (ACF) for early detection of tuberculosis (TB) to achieve the Sustainable Development Goal target of ending TB.

Objective

To explore antecedents, components and influencing factors on ACF policy development and implementation.

Methods

We undertook a scoping review searching MEDLINE, Web of Science, the Cochrane Database of Systematic Reviews and the WHO Library for the period January 1968 to January 2018. We included any type of study written in English. We excluded studies focusing on latent TB infection, passive case-finding, childhood TB and studies about effectiveness, yield, accuracy and impact without descriptions of how this evidence has/could influence ACF policy or implementation. We conducted frequency and thematic analyses.

Results

Seventy-three articles fulfilled our eligibility criteria. Most (67%) were published after 2010. The studies were conducted in all WHO regions, but primarily in Africa (22%), Europe (23%) and the Western-Pacific region (12%). Forty-one percent of the studies were classified as quantitative, followed by reviews (22%) and qualitative studies (12%). Most articles focused on ACF for TB contacts (25%) or migrants (32%). Fourteen percent of the articles described community-based screening of high-risk populations. Fifty-nine percent of studies reported influencing factors for ACF implementation; mostly linked to the health system (e.g. resources) and the community/individual (e.g. social determinants of health). Only two articles highlighted factors influencing ACF policy development (e.g. politics). Six articles

For peer review only - http://bmjopen.bmj.com/site/about/guidelines.xhtml

described WHO's ACF-related recommendations as important antecedent for ACF. Key components of successful ACF implementation include health system capacity, mechanisms for integration, education and collaboration for ACF.

Conclusion

We identified some main themes regarding the antecedents, components and influencing factors for ACF policy development and implementation. However, evidence remains scarce especially concerning policy development. More original research is required to understand, inform and improve policy processes and implementation.

Key words

Tuberculosis, active case-finding, community, policy development, policy implementation

Article summary

Strengths and limitations of this study

- Overview of some main themes regarding the antecedents, components and influencing factors for ACF policy development and implementation.
- Wide scope of influencing factors (barriers and facilitators) were reviewed.
- Reporting of ACF policy development and implementation varied in completeness across the included articles.

Manuscript (Word count: 5567 words)

For peer review only - http://bmjopen.bmj.com/site/about/guidelines.xhtml

Background

Systematic screening for tuberculosis (TB) is defined by the World Health Organization (WHO) as the "systematic identification of people with suspected active tuberculosis (TB), in a predetermined target group, using tests, examinations or other procedures that can be applied rapidly" [1]. Active case-finding (ACF) is synonymous with systematic screening for active TB, although it usually implies screening outside of health facilities.

Tuberculosis is a major global health challenge, particularly in low- and middle-income countries. The WHO End TB Strategy and the Sustainable Development Goals aim at finding the "missing cases" and ending the global TB epidemic by 2030. This will require intensified activity to increase TB case detection, specifically in "hard-to-reach" groups [2]. In 2017, there was a 4.1 million gap between estimated incident and notified TB cases globally, reflecting a combination of an underdiagnosis of cases and underreporting of cases who have been detected [3]. Many people with TB are diagnosed only after long delays [4], causing suffering and economic hardship for TB patients and TB-affected households, and sustained transmission [1].

Active case finding is mostly provider-initiated. It targets people in high-risk groups who may not seek health care actively [1]. Potential benefits for patients include reduced morbidity, mortality and economic consequences due to earlier diagnosis, while society can benefit from reduced transmission and a reduced burden of TB, which often affects the most economically productive members of a society. TB screening in high-risk groups has been implemented in many settings within the TB program or in the context of research and can significantly improve TB case notification [5-7].

For peer review only - http://bmjopen.bmj.com/site/about/guidelines.xhtml

However, if not well targeted and implemented, ACF can be costly leading to diversion and waste of scarce resources, potentially compromising passive case-finding infrastructure and weakening health systems. It can also cause harm to individuals, e.g. by increasing the risk of a false positive diagnosis and providing TB treatment to individuals without TB, or increased stigma and discrimination arising from uncovering TB, which need to be weighed against the benefit of identifying cases in the community that would otherwise go untreated [1,2].

Questions remain about both *if* ACF in general is worthwhile, and *how* to best plan and implement outreach screening through ACF in a given context as a synergistic way, i.e. integrated into the health care system, and not delivered separately in a parallel system. The evidence base is weak concerning the benefits and cost-effectiveness of ACF and how it varies between risk groups [8]. The strength of evidence to support alternative ACF approaches is low, with few head-to-head trial results being available to inform practice. Many differences exist in stakeholder's values and preferences concerning the rationale, outcomes and possible secondary effects of ACF. Despite the relatively weak evidence, WHO has a guideline on systematic screening and the Global Fund [9] and TB REACH [10] provide increasing funding for ACF, while a growing number of countries are increasing investment in and have national policies for ACF. For example, Vietnam's National Strategic Plan 2015-2020 includes ACF in remote and congregate settings, and in high-risk groups including contacts, prisoners, miners, the elderly, homeless people, young adults, migrants, factory workers, communities, people living with Human Immunodeficiency Virus (HIV), multidrug-resistant patients, young males, smokers and intravenous drug users [11.

The aim of this study was to explore antecedents, components and influencing factors for

For peer review only - http://bmjopen.bmj.com/site/about/guidelines.xhtml

ACF policy development and implementation, to inform and improve future ACF policy processes. We did not aim to review the evidence on ACF per se; therefore, we explore yield, accuracy and impact only in the context of how they influence ACF policy development or implementation.

Methods

We conducted a scoping review, using an a-priori protocol based on the following research question: *Which are the antecedents, components and influencing factors (barriers and facilitators) in developing and implementing ACF policies?* The scoping review methodology was deemed appropriate given the breadth of the question. The protocol was based on guidance from the Joanna Briggs Methods Manual for Scoping Reviews [12]. The inclusion criteria, the screening manual and the data-charting table were developed by one author (OB), reviewed by two authors (KV and KL) to ensure face validity, and pilot-tested. All team members were consulted at various stages of the scoping review to provide input on the search, data extraction and charting, and the interpretation of the results. The charted data was summarized in tables, and then condensed resulting in Tables 1 and 2. The Preferred Reporting Items for Systematic reviews and Meta-Analyses Extension for Scoping Reviews (PRISMA-ScR) was used to guide reporting [13]. The completed checklist is available in *Additional file 1.* The protocol is available from the corresponding author upon request.

Data sources and search

We searched MEDLINE, Web of Science, The Cochrane Database of Systematic Reviews and the WHO Library from January 1968 (year of the first publication on ACF in MEDLINE) until January 2018. The search strategy was developed in collaboration with a medical

For peer review only - http://bmjopen.bmj.com/site/about/guidelines.xhtml

librarian from Karolinska Institutet (Carl Gornitzki), and further refined through team discussion. The search strategy for MEDLINE is available in *Additional file 2*. The latter was standardized but adapted to fit the other database searches. The respective search strategies are available from the corresponding author upon request. The results of the literature search were imported into Rayyan.

Inclusion criteria

Eligible studies focused on the detection of active TB disease. The review included studies describing or analysing ACF policy development and implementation. Facilitators and barriers linked to access or treatment were included, if there was a clear link to ACF. Furthermore, the review included studies analysing the use of evidence in ACF policy development and implementation. Studies of any design, conducted in any setting or country, and those published in the English language were eligible for inclusion. We excluded studies focusing only on latent TB infection, passive case-finding and childhood TB, and studies about effectiveness, yield, accuracy and impact without descriptions of how this evidence has/could influence ACF policy. The inclusion of different ACF approaches implies that while many lessons can be learned across different settings, some might not be generalizable.

Screening, data abstraction and charting

One reviewer (OB) initially reviewed titles to remove duplicates, and those studies not focusing on TB. Two reviewers (OB and KV) independently reviewed titles and abstracts, and then full-text articles for inclusion. Conflicts were resolved through discussion, based on the inclusion/exclusion criteria.

The data-charting table was developed in Microsoft Excel. An abridged version of the table is available in *Additional file 3*. Data was extracted on study design, country, target population

For peer review only

for ACF, benefits of ACF, risks of ACF, ACF antecedents, ACF policy development and implementation (use of evidence, country-level to individual-level facilitators and barriers, stakeholders involved), lessons learned, future perspectives and future research. Relevant data was charted by one author (OB), while a second author (KV) verified this step by charting data from a random sample of studies for comparison. Differences in data-charting were resolved by discussion.

Patient and public involvement

This study is related to a qualitative study of experts and a survey of National TB Programme managers combined with a policy document review on the topic of ACF policy development and implementation which are currently under way to harness tacit knowledge on the topic. The research questions and variables for these studies were informed by the results of this scoping review. The results of this study will be presented to researchers and policy-makers in the field via targeted issue briefs. We will also share the results with the public via a video and short messages on social media.

Results

Literature search

After screening 2943 titles and abstracts and 271 full-text articles, 73 unique articles fulfilled our eligibility criteria (*Figure 1*) [14-20,23-60,65-69,71-93]. The reasons for excluding full-text articles are provided in Fig. 1. What all included studies have in common is the goal of finding 'missing' TB cases through ACF, while the approaches vary greatly; from contact investigation to screening people in homeless shelters (Table 1). An example of a study included in this review is a qualitative study by Ayakaka et al., which explored influencing

For peer review only - http://bmjopen.bmj.com/site/about/guidelines.xhtml

factors for TB contact investigation in Kampala, Uganda [29]. The stakeholders who were interviewed described key barriers for ACF as being limited knowledge about TB among contacts, stigma, mistrust of health centre staff among index patients and contacts, and high travel costs for lay health workers and contacts. At the same time, key facilitators for ACF comprised personalized and enabling services provided by lay health workers. To overcome barriers and strengthen facilitators, the researchers identified education, incentivization and restructuring of the service environment as relevant interventions [29].

Characteristics of the included articles (*n*=73)

The breadth of studies identified reflect the complexity and diversity of ACF policies and their characteristics. There has been a gradual increase in the number of articles on ACF since the first identified (e.g. from 1979), and 67% (*n*=49/73) were published in the period 2010-2018 (*Table 1*). The studies were conducted in all WHO regions, i.e. the African region (22%, *n*=16/73), the Americas (North America) (14%, *n*=10/73), the Eastern Mediterranean region (1%, *n*=1/73), the European region (23%, *n*=17/73), South-East Asia (7%, *n*=5/73) and the Western Pacific region (12%, *n*=9/73). The most common types of articles were classified as quantitative papers (41%, *n*=30/73), reviews (22%, *n*=16/73) and qualitative studies (12%, *n*=9/73). The most frequent ACF target groups were contacts (25%, *n*=18/73) and migrants from high-incidence countries (32%, *n*=23/73), while 14% percent (*n*=10/73) of the articles focused on screening of defined communities. The remaining studies focused on different target groups which are specified in *Table 1.*

Table 1 Characteristics of the articles included in the scoping review (n=73)

(Please see end of text file.)

Fig 1. PRISMA flow diagram

For peer review only

(Please see separate Word file entitled “Biermann_fig 1".)

Antecedents

Active case-finding has been implemented for many decades primarily in high-income countries, starting with mass screening campaigns in the general population in the 1950s and 1960s, then moving towards specific risk populations in recent decades, such as migrants from high-incidence countries and prison populations [14]. In low- and middle-income countries, the interest in ACF has increased in recent years, mainly as a response to a sustained case detection gap documented in annual Global TB Reports produced by WHO [3] and the emergence of new WHO guidelines [15].

Generally, TB programmes have moved from traditionally vertical approaches and exclusively facility-based case finding among people seeking care with TB symptoms (so-called “passive case-finding”) towards being closer to the client through decentralized, community-based solutions and outreach activities, including ACF – similar to community-based testing services that have increased access to HIV testing and care [16,17].

In 1976, the WHO Expert Committee on TB recommended that countries abandon *indiscriminate* mobile mass radiography, as evidence showed the inefficiency of population-wide screening in settings that had seen TB rates drop dramatically since the second World War [18]. It should be noted that the Expert Committee emphasized that TB screening should still be done in selected risk groups [18,19].

The current WHO guidelines on systematic screening for active TB were released in 2013 [1]. These guidelines still discourage *indiscriminate* mass screening and strongly recommend ACF only in selected high-risk groups, while conditionally recommending screening in other high-risk groups. Conditional recommendations are those for which the benefits are expected to outweigh the risks, while the trade-offs, such as cost-effectiveness, feasibility or

For peer review only - http://bmjopen.bmj.com/site/about/guidelines.xhtml

affordability may be uncertain. For example, WHO has made a conditional recommendation (very low-quality evidence) for screening certain clinical risk groups in settings where the TB prevalence is over 100 per 100,000 population (while not recommending such screening in countries with a prevalence of less than 100 per 100,000 population) [1].The 2013 WHO guidelines – reflected in the latest 2015 WHO's End TB Strategy [2] and aligned with the Sustainable Development Goals – put ACF back on the agenda. The guidelines provide principles for how to test and expand an approach that had been largely missing in previous global TB strategies, including the Directly Observed Treatment, Short Course (DOTS) Strategy from 1994 [21] and the Stop TB Strategy 2006-2015 (although contact investigation and screening people living with HIV was included in the latter) [22]. Moreover, the guidelines emphasize the importance of basing ACF policy on local epidemiology and to perform careful evaluations of ACF implementation, yield and impact to improve the ACF evidence base [19]. Today, ACF is considered a global health priority [23,24].

Components

According to WHO [1], the main components of ACF include 1) having high-quality TB diagnosis, treatment, care, management and support for patients, as well as the capacity to scale these up (if needed), before screening is initiated; 2) prioritizing risk groups; 3) choosing a suitable screening algorithm; 4) following established ethical principles; 5) optimizing synergies with the delivery of other health and social services; and 6) monitoring and re-assessing ACF approaches.

The components of ACF, as well as those of the wider health system that ACF depends on, are well-described in the literature. It is important for high-quality diagnosis, treatment, care, management, patient support and capacity for scale-up to be available [18]. Risk groups must be prioritized based on assessments of benefits and risks, feasibility, acceptability, the number

For peer review only

needed to screen to detect a case of TB and the cost-effectiveness of ACF [18], while also considering the heterogeneity of the risk group [25].

The overall ACF approach must be flexible, patient-centred [15,26] and culturally sensitive [27]. In resource poor settings, ACF is often backed by multi-channel financing mechanisms, including from international donors [28]. Record-keeping, monitoring and evaluation are described as being essential [29,30]. Incentives are sometimes deemed necessary, e.g. for health workers [5,32], laboratory technicians [73], screening participants [11,36,40,42,43,67] and TB patients [32,51,67]. The articles also emphasized ACF-specific health education as paramount – for patients [20,34,35,36], their families [37], providers [26] and community health workers (CHW) [38,39]. The training of CHWs should be followed by supervision [38,39]. In addition, community involvement [35,39,40,41] and social mobilization [32] are major parts of ACF. One study described how the involvement of community leaders was a key component to improve trust among patients and contacts [36].

Lastly, the studies underlined how vital it is to collaborate with other disease programmes [41,42,43], agencies and sectors [28,30], and to integrate ACF within existing systems [18,44]. The latter includes collaboration between providers and community-based organizations [41], and between HIV and TB services [33].

Influencing factors

In the following section, we summarize influencing factors for ACF policy development and implementation. We chose to speak of “influencing factors” rather than facilitators and barriers, as one and the same factor might be a facilitator in one context and a barrier in another, e.g. health workers’ satisfaction might be a powerful facilitator for ACF [39], while the lack thereof could be a strong barrier as well [46].

For peer review only - http://bmjopen.bmj.com/site/about/guidelines.xhtml

Influencing factors for ACF policy development

Active case-finding policy development processes were not well described in the literature. Policy development comprises the investment of governments and donors in ACF. Only two articles reported facilitators and/or barriers for ACF policy development: politics [46,47] and laws [47]. Welshman [46] describes how, in the mid-1950s, the Ministry of Health in the United Kingdom subverted pressure from stakeholders who were in favour of compulsory ACF at ports of entry – the Ministry "argued that the problem [of TB in migrants] was a minor one" so a policy was not developed. On the other hand, laws such as the 1958 Commonwealth Migration Act were described, which made screening compulsory, and thus influenced ACF policy development in the United Kingdom [47].

Influencing factors for ACF policy implementation

Policy implementation processes in the area of ACF are widely researched. In total 43, articles reported barriers and/or facilitators for ACF implementation (*Table 2*). Most articles mentioned factors at the level of the health system, as well as the individual and community level.

Table 2 Barriers and facilitators for active case-finding policy implementation (n=43)

(Please see end of text file.)

Health system level

It is fundamental to consider health system factors when putting ACF policy into practice and identifying which factors influence ACF policy implementation the most. We derived six major themes from the articles: 1) availability of financial resources, 2) existing systems and

For peer review only - http://bmjopen.bmj.com/site/about/guidelines.xhtml

structures, 3) availability of diagnostic tests, 4) staff experience and motivation, 5) collaboration between different actors, and 6) implementation of a person-centred approach.

Availability of financial resources

Many articles (35%, *n*=15/43) mentioned financial resources as an influencing factor for ACF policy implementation. Finances were described as being scarce at different levels of the health system [37], for the provision of staff [35,39], drugs and equipment [48].

Existing systems and structures

Existing systems and structures were reported as influencing factors for ACF policy implementation by 26% (*n*=11/43) of the articles. Studies elaborated on the availability of health and social services [29,18] and laboratory networks [23] that could be used for ACF implementation.

Availability of diagnostic tests of sufficient sensitivity and specificity

Nineteen percent (*n*=8/43) of the articles elaborated on the availability of diagnostic tests and services as key influencing factor for ACF policy implementation – with many articles referring to the availability of molecular diagnostic tests such as Xpert MTB/RIF® [30,49,50,51]. Decentralization of health care services, national coverage of diagnostics and task sharing were perceived as facilitators of ACF policy implementation, but unavailable in many settings [42].

Staff experience and motivation

Thirty-three percent (*n* =14/43) of the studies described staff experience and motivation as influencing factors for ACF policy implementation. One study elaborated on how, on the one hand, staff members would feel motivated by increased case detection and awareness-raising through ACF. On the other hand, they would be frustrated when being “shouted at, ignored, (and) harassed” by target communities, in this case people living in poor urban settlements

For peer review only

[35]. The over-burdening of staff was highlighted by 16% (*n*=7/43) of the articles, e.g. leading to absenteeism [52], and thus inhibiting ACF implementation.

Collaboration between different actors

A variety of studies (16%, *n*=7/43) described collaboration as an important influencing factor for ACF policy implementation. One example is the collaboration between community workers, health centre staff and laboratory technicians in delivering diagnostic services as part of ACF [15]. Moreover, Akkerman et al. described the positive impact on diagnostic accuracy and the proportion of follow-up medical interventions when there is close collaboration with a limited number of experienced, high-volume chest radiograph readers when screening asylum seekers and other high-risk groups [77].

Implementation of a person-centred approach

The need for a person-centred approach to ACF policy implementation was referred to by 19% (*n*=8/43) of the articles, e.g. adopting approaches including health care providers being non-judgmental and supportive of patients and their families during diagnosis and follow-up, [53] and assuring confidentiality [24,54]. Another 12% (*n*=5/43) of the articles emphasized health education for the general public, screening participants, as well as TB patients and their families as a necessity.

Individual and community

Active case-finding policies should be *for* individuals and communities and their influence on successful implementation is elaborately described in the literature. Three overarching themes emerged from the studies: 1) stigma and discrimination, 2) individual characteristics and socio-cultural factors, and 3) knowledge and awareness.

Stigma and discrimination

Many studies (37%, *n*=16/43) reported stigma and discrimination linked to TB as influencing

For peer review only - http://bmjopen.bmj.com/site/about/guidelines.xhtml

factors for ACF policy implementation. The consequences could be the avoidant behaviour of a contact of an index TB case, such as providing incorrect phone numbers and/or wrong directions to their homes [29], or misconceptions about TB [26,41]. Issues of fear, either of TB disease [25,35,53], the process of ACF [55], or the wider implications of being identified as a TB case, e.g. through migrant screening programmes, where being diagnosed with TB case has in some instances resulted in deportation [34,43,56,57]. Studies also reported the lack of trust of patients and their families as key barrier for ACF policy implementation [38,52] – not only for early diagnosis, but for the sustained commitment to addressing social determinants of TB [58].

Individual characteristics and socio-cultural factors

Twenty-eight percent (*n*=12/43) of the articles mentioned the social characteristics of the persons screened as key influencing factors for ACF policy implementation, e.g. for ensuring equitable access to care [23,35,59] and promoting conducive health-seeking behaviour [25,38,41]. Moreover, the articles frequently reported socio-cultural factors and language (33%, *n*=14/43) as influencing factors for ACF policy implementation. Culture was commonly described as a barrier to ACF policy implementation [38,43,60], e.g. if cultural beliefs would negatively influence the receptiveness to community health workers' advice. Only one study mentioned culture as a facilitator for putting ACF into practice, i.e. if culture meant respect toward professional authority [60].

Knowledge and awareness

Lastly, 19% (*n*=8/43) of the articles reported factors related to knowledge and awareness as influencing factors for ACF policy implementation. One such factor could be the understanding of the purpose of ACF [26]. Also, knowledge and awareness are closely linked

For peer review only - http://bmjopen.bmj.com/site/about/guidelines.xhtml

to persisting stigma and discrimination, e.g. Ayakaka et al. [29] describe how health workers attributed TB-associated stigma to a lack of general knowledge in the community about TB.

Discussion

Characteristics of the included articles

We identified 73 articles addressing antecedents, components and/or influencing factors for ACF policy development and implementation. The included studies originated from all WHO regions. Africa and Asia are home to 90% of the 30 high TB burden countries (53% and 37% respectively) [61], which may explain why studies were more frequently conducted from these continents: 22% of the studies were conducted in Africa, 12% in the Western-Pacific region and 7% in South-East Asia. Studies conducted in the European region (23%) were predominantly about different forms of migrant screening. There was no study from Latin America among the 73 included articles, and there were no articles in the Spanish language among those excluded.

Most studies were published after 2010, which may be a result of programmes implementing WHO guidelines on systematic screening [1] as well as the growing number of prevalence surveys in different settings [35]. We classified 41% of the articles as quantitative, e.g. cross-sectional and cohort studies, effectiveness and cost-effectiveness studies, randomized controlled trials and programme evaluations. The next most common study types were reviews (22%) and qualitative studies (12%). The first qualitative studies on our topic were published in 2003 [26,56]. We only identified two mixed-methods studies, one published in 2013 [39] and one in 2015 [35]. The mixed-methods design combined with quantitative ACF evaluation might become more commonly used to explore the complexity of ACF policy development and implementation in the future, as it has the potential to both increase contextual understanding and reduce biases.

For peer review only

Amongst the broad range of possible target groups for ACF, most of the articles focused either on TB contacts or migrants. The focus on the latter was mainly in low-incidence countries and may be a result of long-standing national health and migration policies supporting these types of screening. Yet, other studies covered vulnerable groups, such as homeless people, people living with HIV or people living in congregate settings, as well as the urban poor. Some articles included analysis of more than one high-risk group. There were no studies on indiscriminate mass screening. However, there were studies targeting broad communities, e.g. communities with high HIV prevalence.

Antecedents

Despite the growing number of studies (e.g. operational studies), there is still a relative lack of evidence about the health benefits for individuals, the epidemiological impact and cost-effectiveness of different ACF approaches. Nonetheless, the interest in ACF has clearly increased in recent years. The persistent case detection gap, the 2013 WHO guidelines on systematic screening [1], and ACF being mentioned as a core component of the End TB Strategy [2] seem to have motivated countries to include ACF in national TB strategies. Universal Health Coverage and the Sustainable Development Goals have only marginally been mentioned as important for stepping up the TB response [42]. However, the Sustainable Development Goals – which aim to "leave no one behind" – might have contributed to a greater interest in ACF to leave no undiagnosed TB patients behind.

Not having enough evidence to drive ACF policy development and implementation means that decisions to embark on ACF rely largely on stakeholders' tacit knowledge, experience, values and preferences. Demonstrated screening yield in the same or other settings could also motivate further ACF implementation, although the number of cases detected may not be a relevant public health impact measure in itself. The above-mentioned WHO guidelines use the

For peer review only - http://bmjopen.bmj.com/site/about/guidelines.xhtml

term "indirect evidence" [1]. Strong "direct evidence" exists that early diagnosis and correct TB treatment reduce morbidity, mortality and transmission. This can constitute, for example, "indirect evidence" for ACF, based on the logic that if ACF leads to early detection and treatment then it should also lead to better health outcomes and less transmission, despite the lack of trial data demonstrating a direct health impact of ACF. Similar reasoning can be used to describe potential harms associated with ACF, e.g. based on modelling of the risk of false positive TB diagnoses in different epidemiological contexts. "Indirect evidence", tacit knowledge, experience, values and preferences are likely to be important antecedents for national policy.

Components

The components of ACF policy, and of the system which ACF is part of, are complex. The articles reported on the prerequisites of the given system, its resources [46], capacity [39,73] and tools [62]. However, they also elaborated on the need for integrating ACF into health systems [38,45], as well as educating and engaging health care providers [5,23], communities [12,32,35,44], screening participants [67] and TB patients and their families [27,43,67] for ACF implementation to succeed. All components seem indispensable for successful ACF policy development and implementation. Yet, in real-life settings, rarely all components are in place.

Which ACF components are essential and which are non-essential? The 2013 WHO guidelines on systematic screening list six key principles for screening for active TB, the first principle being: "Before screening is initiated, high-quality TB diagnosis, treatment, care, management and support for patients should be in place, and there should be the capacity to scale these up further to match the anticipated rise in case detection that may occur as a result of screening" [1]. Yet, countries might not even completely adhere to this very first principle,

For peer review only - http://bmjopen.bmj.com/site/about/guidelines.xhtml

e.g. due to a lack of resources, before a country embarks on ACF policy development and implementation.

Influencing factors

Scarce research on ACF policy development

Our findings indicate a paucity of research focusing specifically on ACF policy development. The only two factors described – politics and laws – were mentioned by the same author in two different articles and concerned migrant TB screening in a low-incidence country [46,47]. Even though not documented as such in the literature, data on the epidemiological situation of a country and local evidence regarding impact are assumed to be key factors influencing ACF policy development [1]. These types of data and evidence are needed to identify high-risk groups [62] and devise appropriate screening algorithms. An assessment of the epidemiological situation might be based on data from regularly collected datasets or prevalence surveys, and WHO has developed a tool to help countries estimate screening yield and costs based on such data to identify appropriate risk groups and approaches [63]. Interestingly, the evolution of the funding landscape for ACF, e.g. through TB REACH, has not been explicitly mentioned as an influencing factor for ACF policy development or implementation, though it has been a likely game-changer. TB REACH is a case-finding initiative funded largely by Global Affairs Canada and coordinated by the Stop TB Partnership [64]. The initiative has not only funded ACF activities but raised awareness about the importance of ACF. It has contributed to the local evidence base, while the uptake of the evidence generated by TB REACH may be considered but not mentioned in the studies included in this review. Four articles mentioned TB REACH as funder of their research, with the disclaimer that the initiative had no role in study design, data collection and analysis

For peer review only - http://bmjopen.bmj.com/site/about/guidelines.xhtml

[15,39,45,65]. Other publications acknowledged its role in implementing ACF when describing the study methods, e.g. that TB REACH funded door-to-door screening [35] or the implementation of different ACF models [66] and that the initiative upgraded an ACF strategy by introducing new diagnostics which still have limited availability in many settings (e.g. Xpert MTB/RIF®) [51]. It is important to reflect on and document the influence that TB REACH, other international funding mechanisms and technical agencies have on ACF policy development and implementation.

ACF policy and social determinants of health

Many barriers and facilitators for implementing ACF policies lie at the level of communities and individuals. These factors include social determinants such as the educational level [29,67], employment status [30,68] and access to care [69] of the target population. These factors can limit an individual's or a community's participation in ACF. ACF policies cannot be put in practice successfully if implementation barriers prevail. However, rather than talking about "missing cases" and communities that are "hard to reach", it may be useful to consider if health services are missing or hard to reach instead.

Individuals or communities may be ACF target groups precisely because health services are missing or hard to reach. As such, ACF could be a way to by-pass access barriers, e.g. by using mobile units. Yet, even when designing services to be more accessible, target populations may still not come forward, take the next step of referral to complete the diagnostic pathway or adhere to treatment due to socioeconomic barriers.

We argue that active case-finding polices thus must be designed and implemented with careful consideration of social determinants in a given context to avoid possible harm.

Whitehead et al. [70] described how interventions designed to help vulnerable populations

For peer review only - http://bmjopen.bmj.com/site/about/guidelines.xhtml

may be "implemented in such a way as to stigmatize the very people the programme was designed to help and, in so doing, push them to avoid the help on offer."

A comprehensive view on ACF policies

A comprehensive approach in developing and implementing ACF policies is required that includes health education as an important component to ACF policies, and that ensures collaboration between key stakeholders, not only between patients and providers, but between providers and local health authorities, between agencies, and across sectors and disease programmes.

Although this review focused on the factors that influence ACF policy and implementation, it is important to also reflect on how ACF policy influences its context in return, e.g. not only factors at the level of the community and the individual, but at the level of the health system and beyond; How may the ACF policy increase or decrease stigma in a community? How may ACF policy influence the human and financial resources available in a health system? Only then may we see the real impact of ACF and be able to make equitable decisions in policy development and implementation processes.

Future research

This scoping review demonstrated that while we know much about facilitators and barriers for ACF policy implementation, we know less about how to strengthen those facilitators and how to overcome those barriers. A major knowledge gap remains when it comes to understanding which contextual factors influence ACF policy development.

Limitations

For peer review only - http://bmjopen.bmj.com/site/about/guidelines.xhtml

Our scoping review has some limitations. To increase the feasibility, we limited the search to MEDLINE, Web of Science, The Cochrane Database of Systematic Reviews and the WHO Library. We did not do a critical appraisal of the individual sources of evidence or within sources of evidence, nor did we describe sources of funding for the included sources of evidence. Such appraisals and descriptions would have allowed us to not only discover the scarcity of research, but to characterize the quality of and gaps in the evidence base and enable the development of robust recommendations. We leave these questions for future systematic reviews to address.

We acknowledge that we may have been able to provide a more in-depth overview of antecedents, components and influencing factors for ACF policy development and implementation by including additional databases, searching grey literature and references of included studies, and contacting authors for more information, which could be included in a future systematic review. However, we believe that our review still adds value to the current body of knowledge on ACF, by providing collated and comprehensive insights into the peer-reviewed scientific literature. We also limited the inclusion to studies written in English, which may have resulted in the exclusion of eligible studies in other languages, e.g. Spanish. Furthermore, the results from this scoping review are only up to date as of 31 January 2018. The data were extracted by one reviewer only. However, the data are likely valid, as a pilot-test was conducted prior to embarking on data extraction with members of the study team. A second reviewer verified a random selection of the data.

Another limitation was that the included articles often did not distinguish between the implementation of an ACF policy versus the implementation of an ACF programme or intervention. This is likely due to inconsistent use of the terms in the literature. As such, our results are likely applicable to both. Furthermore, the reporting of ACF policy development and implementation varied in their completeness across the included articles, and as such, our

For peer review only - http://bmjopen.bmj.com/site/about/guidelines.xhtml

For peer review only

data are limited by the details described in the literature, e.g. most papers described steps to implementation, but did not provide details on non-response or unsuccessful practices. There is a risk of publication bias.

Conclusions

We identified some main themes regarding the antecedents, components and influencing factors for ACF policy development and implementation. However, evidence remains scarce especially concerning policy development. More original research on ACF is required to understand, inform and improve policy processes and implementation.

List of abbreviations

ACF – Active Case-Finding

ART – Antiretroviral Therapy

CHW – Community Health Worker

DOTS – Directly Observed Treatment, Short Course

HIV – Human Immunodeficiency Virus

LTBI – Latent Tuberculosis Infection

PCF – Passive Case-Finding

PRISMA - Preferred Reporting Items for Systematic reviews and Meta-Analyses

PRISMA-ScR – Preferred Reporting Items for Systematic reviews and Meta-Analyses extension for Scoping Reviews

TB – Tuberculosis

TiAb – Title and abstract

WHO – World Health Organization

For peer review only - http://bmjopen.bmj.com/site/about/guidelines.xhtml

Ethics approval and consent to participate

N/A

Consent for publication

N/A

Availability of data and material

The materials supporting the conclusions of this article are included within the article and its additional files.

Additional files

Additional file 1: PRISMA Extension for Scoping Reviews (PRISMA-ScR) checklist.

Additional file 2: Literature search strategy used for Medline; additional search strategies available from the authors.

Additional file 3: Abridged data charting table.

Competing interests

We have no competing interests to declare.

Funding

This work was supported by the EU-Horizon 2020-funded IMPACT-TB project (grant 733174).

Author contributions

OB, KL, MC and KV conceived the study. OB developed the search strategy together with a medical librarian from Karolinska Institutet. OB and KV screened titles and abstracts, and then full-text articles. OB extracted, charted, analysed and interpreted the data. KV verified the data extraction by charting data from a random sample of studies for comparison. OB wrote the first draft of the manuscript. KL, MC and KV were consulted at various stages of the scoping review to provide input on the search strategy, data extraction, charting, analysis and the interpretation of the results, and provided comments on the manuscript. All authors read and approved the final manuscript.

Acknowledgements

OB is funded by the EU-Horizon 2020-funded IMPACT-TB project (grant 733174). The authors thank Carl Gornitzki, medical librarian at Karolinska Institutet, for supporting the development of the search strategy, and Gabriella Ekman, writing instructor at Karolinska Institutet's library for her valuable feedback in writing this manuscript.

References

For peer review only - http://bmjopen.bmj.com/site/about/guidelines.xhtml

639 1. World Health Organization. Systematic screening for active TB: principles and
640 recommendations. Switzerland, Geneva: World Health Organization; 2013.
641 2. World Health Organization. Implementing the End TB Strategy: the essentials.
642 Switzerland, Geneva: World Health Organization; 2015.
643 3. World Health Organization. Global TB Report 2017. Switzerland, Geneva: World
644 Health Organization; 2017.
645 4. Storla DG, Yimer S, Bjune GA. A systematic review of delay in the diagnosis and
646 treatment of TB. BMC Public Health. 2008;8:15. doi: 10.1186/1471-2458-8-15.
647 5. Blok L, Sahu S, Creswell J, Alba S, Stevens R, Bakker MI. Comparative meta-
648 analysis of TB contact investigation interventions in eleven high burden countries.
649 PLoS One. 2015;10:3:e0119822. doi: 10.1371/journal.pone.0119822.
650 6. Creswell J, Codlin AJ, Andre E, Micek MA, Bedru A, Carter EJ, et al. Results from
651 early programmatic implementation of Xpert MTB/RIF testing in nine countries. BMC
652 Infect Dis. 2014;14:2. doi: 10.1186/1471-2334-14-2.
653 7. Creswell J, Sahu S, Blok L, Bakker MI, Stevens R, Ditiu L. A multi-site evaluation of
654 innovative approaches to increase TB case notification: summary results. PLoS One.
655 2014;9:4:9:e94465. doi: 10.1371/journal.pone.0094465.
656 8. Kranzer K, Afnan-Holmes H, Tomlin K, Golub JE, Shapiro AE, Schaap A, et al. The
657 benefits to communities and individuals of screening for active TB disease: a
658 systematic review. Int J Tuberc Lung Dis. 2013;17:432–46. doi: 10.5588/ijtld.12.0743.
659 9. Global Fund, World Health Organization and Stop TB Partnership Strategic Initiative.
660 https://www.who.int/tb/joint-initiative/en/ (2019). Accessed 8 Apr 2019.
661 10. TB REACH. http://www.stoptb.org/global/awards/tbreach/ (2019). Accessed 8 Apr
662 2019.

For peer review only - http://bmjopen.bmj.com/site/about/guidelines.xhtml

11. National TB Control Program. National Strategic Plan for TB control for the period 2015-2020. Vietnam, Hanoi: Ministry of Health; 2014.

12. Joanna Briggs Institute. Methodology for JBI scoping reviews. Adelaide: Joanna Briggs Institute; 2015.

13. Tricco A, Lillie E, Zarin W, O'Brien KK, Colquhoun H, Levac D, et al. PRISMA Extension for Scoping Reviews (PRISMA-ScR): Checklist and Explanation. Ann Intern Med. 2018;169:467-473. doi: 10.7326/M18-0850.

14. Eang MT, Satha P, Yadav RP, Morishita F, Nishikiori N, van-Maaren P, Lambregts-van Weezenbeek C. Early detection of tuberculosis through community-based active case finding in Cambodia. BMC Public Health. 2012;12:469. doi: 10.1186/1471-2458-12-469.

15. Lorent N, Choun K, Thai S, Kim T, Huy S, Pe R, et al. Community-Based Active Tuberculosis Case Finding in Poor Urban Settlements of Phnom Penh, Cambodia: A Feasible and Effective Strategy. PLoS One. 2014;9:3:e92754. doi: 10.1371/journal.pone.0092754.

16. Godfrey-Fausset P, Ayles H. Can we control tuberculosis in high HIV prevalence settings? Tuberculosis. 2003;83:68-76. doi: 10.1016/S1472-9792(02)00083-5.

17. Corbett EL, MacPherson. Tuberculosis screening in high human immunodeficiency virus prevalence settings: turning promise into reality. Int J Tuberc Lung Dis. 2013;17:9:1125-1138. doi: 10.5588/ijtld.13.0117.

18. Lönnroth K, Corbett E, Golub J, Godfrey-Faussett P, Uplekar M, Weil D, Raviglione M. Systematic screening for active tuberculosis: rationale, definitions and key considerations. Int J Tuberc Lung Dis. 2013;17:3:289-298. doi: 10.5588/ijtld.12.0797 STATE.

For peer review only

19. Zenner D, Hafezi H, Potter J, Capone S, Matteelli A. Effectiveness and cost-effectiveness of screening migrants for active tuberculosis and latent tuberculosis infection. Int J Tuberc Lung Dis. 2017;21:9:965-976. doi: 10.5588/ijtld.16.0935.

20. Fox GJ, Loan LP, Nhung NV, Loi NT, Sy DN, Britton WJ, Marks GB. Barriers to adherence with tuberculosis contact investigation in six provinces of Vietnam: a nested case-control study. BMC Infect Dis. 2015;15:103. doi: 10.1186/s12879-015-0816-0.

21. World Health Organization. What is DOTS? A Guide to Understanding the WHO-recommended TB Control Strategy Known as DOTS. Switzerland, Geneva: World Health Organization; 1999.

22. World Health Organization. Building on and enhancing DOTS to meet the TB-related Millennium Development Goals. Switzerland, Geneva: World Health Organization; 2006.

23. Golub JE, Mohan CI, Comstock GW, Chaisson RE. Active case finding of tuberculosis: historical perspective and future prospects. Int J Tuberc Lung Dis. 2005;9:11:1183-1203. [PMID: 16333924]

24. Kerrigan D, West N, Tudor C, Hanrahan C, Lebina L, Msandiwa, et al. Improving active case finding for tuberculosis in South Africa: informing innovative implementation approaches in the context of the Kharitode trial through formative research. Health Res Policy Sys. 2017;15:42. doi: 10.1186/s12961-017-0206-8.

25. Swigat V, Kolb R. Homeless persons' decisions to accept or reject public health disease-detection services. Public Health Nurs. 2004;21:2:162-170. doi: 10.1111/j.0737-1209.2004.021210.x.

26. Shrestha-Kuwahara R, Wilce M, DeLuca N, Taylor Z. Factors associated with identifying tuberculosis contacts. Int J Tuberc Lung Dis. 2003;7:12:S510-516

For peer review only - http://bmjopen.bmj.com/site/about/guidelines.xhtml

27. de Vries SG, Cremers AL, Heuvelings CC, Greve PF, Visser BJ, Bélard S, et al. Barriers and facilitators to the update of tuberculosis diagnostic and treatment services by hard-to-reach populations in countries of low and medium tuberculosis incidence: a systematic review of qualitative literature. Lancet Infect Dis. 2017;17:e128-43. doi: 10.1016/S1473-3099(16)30531-X.

28. Li J, Liu X-Q, J S-W, Li X, Yu F, Wang Y, et al. Improving tuberculosis case detection in underdeveloped multi-ethnic regions with high disease burden: a case study of integrated control program in China. Infect Dis Poverty. 2017;6:151. doi: 10.1186/s40249-017-0365-4

29. Ayakaka I, Ackerman S, Ggita JM, Kajubi P Dowdy D, Haberer EF, et al. Identifying barriers to and facilitators of tuberculosis contact investigation in Kampala, Uganda: a behavioral approach. Implementation Sci. 2017;12:33. doi: 10.1186/s13012-017-0561-4.

30. Baxter S, Goyder E, Chambers D, Johnson M, Preston L, Booth A. Interventions to improve contact tracing for tuberculosis in specific groups and in wider populations: an evidence synthesis. Health Services and Delivery Research. 2017;5:1. doi: 10.3310/hsdr05010.

31. Van den Bosch CA, Roberts JA. Tuberculosis screening of new entrants; how can it be made more effective? Journal Public Health Med. 2000;22:2:220-223.

32. Hinderaker SG, Rusen ID, Chiang C-Y, Yan L, Heldal E, Enarson DA. The FIDELIS initiative: innovative strategies for increased case finding. Int J Tuberc Lung Dis. 2011;15:1:71-76.

33. Kranzer K, Lawn SD, Meyer-Rath G, Vassall A, Raditlhalo E, Govindasamy D, et al. Feasibility, Yield, and Cost of Active Tuberculosis Case Finding Linked to a Mobile

For peer review only - http://bmjopen.bmj.com/site/about/guidelines.xhtml

736 HIV Service in Cape Town, South Africa: A Cross-sectional Study. PLoS Med.
737 2012;9:8:e1001281. doi: 10.1371/journal.pmed.1001281.
738 34. Kulane A, Ahlberg BM, Berggren I. "It is more than the issue of taking tablets": The
739 interplay between migration policies and TB control in Sweden. Health Policy.
740 2010;97:26-31. doi: 10.1016/j.healthpol.2010.02.014.
741 35. Lorent N, Choun K, Malhotra S, Koeut P, Thai S, Khun KE, et al. Challenges from
742 Tuberculosis Diagnosis to Care in Community-Based Active Case Finding among the
743 Urban Poor in Cambodia: A Mixed-Methods Study. PLoS One. 2015;10:7:e0130179.
744 doi: 10.1371/journal.pone.0130179.
745 36. Tupasi TE, Radhakrishna S, Co VM, Villa MLA, Quelapio MID, Mangubat NV, et al.
746 Bacillary disease and health seeking behavior among Filipinos with symptoms of
747 tuberculosis: implications for control. Int J Tuberc Lung Dis. 2000;4:12:1126-1132.
748 37. Karki B, Kittel G, Bolokon I, Duke T. Active community-based case finding for
749 tuberculosis with limited resources: estimating prevalence in a remote area of Papua
750 New Guinea. Asia Pac J Public Health. 2017;28:1:17-27. doi:
751 10.1177/1010539516683497.
752 38. Getnet F, Hashi A, Mohamud S, Mowlid H, Klinkenberg E. Low contribution of
753 health extension workers in identification of persons with presumptive pulmonary
754 tuberculosis in Ethiopian Somali Region pastoralists. BMC Health Serv Res.
755 2017;17:193. doi: 10.1186/s12913-017-2133-3.
756 39. Yassin MA, Datiko DG, Tulloch O, Markos P, Aschalew M, Shargie EB, et al.
757 Innovative community-based approached doubled tuberculosis case notification and
758 improve treatment outcome in Southern Ethiopia. PLoS One. 2013;8:5:e63174. doi:
759 doi:10.1371/journal.pone.0063174.

For peer review only - http://bmjopen.bmj.com/site/about/guidelines.xhtml

760 40. Cook VJ, Shah L, Gardy J. Modern contact investigation methods for enhancing
761 tuberculosis control in Aboriginal communities. Int J Circumpolar Health.
762 2012;71:18643. doi: 10.3402/ijch.v71i0.18643.
763 41. Seedat F, Hargreaves S, Friedland JS. Engaging New Migrants in Infectious Disease
764 Screening: A Qualitative Semi-Structured Interview Study of UK Migrant Community
765 Health-Care Leads. PLoS One. 2014;9:10:e108261. doi:
766 10.1371/journal.pone.0108261.
767 42. Suthar AB, Zachariah R, Harries AD. Ending tuberculosis by 2030: can we do it? Int J
768 Tuberc Lung Dis. 2016;20:9:1148-1154. doi: 10.5588/ijtld.16.0142.
769 43. Tankimovich M. Barriers to and interventions for improved tuberculosis detection and
770 treatment among homeless and immigrant populations: a literature review. J
771 Community Health Nurs. 2013;30:83-95. doi: 10.1080/07370016.2013.778723.
772 44. Sekandi JN, Dobbin K, Oloya J, Okwera A, Whalen CC, Corso PS. Cost-Effectiveness
773 Analysis of Community Active Case Finding and Household Contact Investigation for
774 Tuberculosis Case Detection in Urban Africa. PLoS One. 2015;10:2:e0117009. Doi:
775 10.1371/journal.pone.0117009.
776 45. Datiko DG, Yassin MA, Theobald SJ, Blok L, Suvanand S, Creswell J, Cuevas LE.
777 Health extension workers improve tuberculosis case finding and treatment outcome in
778 Ethiopia: a large-scale implementation study. BMJ Glob Health. 2017;2:e000390. doi:
779 10.1136/ bmjgh-2017-000390.
780 46. Welshman J. Compulsion, localism, and pragmatism: the micro-politics of
781 tuberculosis screening in the United Kingdom, 1950-1965. Soc Hist Med.
782 2006;19:2:295-312. doi: 10.1093/shm/hkl002.

For peer review only - http://bmjopen.bmj.com/site/about/guidelines.xhtml

47. Welshman J, Bashford A. Tuberculosis, migration, and medical examination: lessons from history. J Epidemiol Community Health. 2006;60:282-284. doi: 10.1136/jech.2005.038604.

48. Mor Z, Lerman Y, Leventhal A. Pre-immigration screening process and pulmonary tuberculosis among Ethiopian migrants in Israel. Eur Respir J. 2008;32:413-418. doi: 10.1183/09031936.00145907.

49. Abubakar I, Stagg H, Cohen T, Mangtani P, Rodrigues LC, Pimpin L, Watson JM, Squire SB, Zumla A. Controversies and unresolved issues in tuberculosis prevention and control: a low-burden-country perspective. J Infect Dis. 2012;205:S293-300. doi: 10.1093/infdis/jir886.

50. Dierberg KL, Dorjee K, Salvo F, Cronin WA, Boddy J, Cirillo D, Sadutshang T, et al. Improved detection of tuberculosis and multidrug-resistant tuberculosis among Tibetan refugees, India. Emerg Infect Dis. 2016;22:3. doi: 10.3201/eid2203.140732.

51. Morishita F, Yadav R-P, Eang MT, Saint S, Nishikiori N. Mitigating Financial Burden of Tuberculosis through Active Case Finding Targeting Household and Neighbourhood Contacts in Cambodia. PLoS One. 2016;11:9:e0162796. doi: 10.1371/journal.pone.0162796.

52. Harper I, Fryatt R, White A. Tuberculosis case finding in remote mountainous areas – are microscopy camps of any value? Experience from Nepal. Tuber Lung Dis. 1996;77:384-388.

53. Southern A, Premaratne N, English M, Balazs J, O'Sullivan D. Tuberculosis among homeless people in London: an effective model of screening and treatment. Int J Tuber Lung Dis. 1999;3:11:1001-1008.

54. O'Hara NN, Roy L, O'Hara LM, Spiegel JM, Lynd LD, FitzGerald JM, et al. Healthcare Worker Preferences for Active Tuberculosis Case Finding Programs in

For peer review only

For peer review only - http://bmjopen.bmj.com/site/about/guidelines.xhtml

South Africa: A Best-Worst Scaling Choice Experiment. PLoS One. 2015;10:7: e0133304. doi: 10.1371/journal.pone.0133304.

55. Brewin P, Jones A, Kelly M, McDonald M, Beasley E, Sturdy P, et al. Is screening for tuberculosis acceptable to immigrants? A qualitative study. J Public Health. 2006;28:3:253-260. doi: 10.1093/pubmed/fdl031.

56. Chemtob D, Leventhal A, Weiler-Ravell D. Screening and management of tuberculosis in immigrants: the challenge beyond professional competence. Int J Tuberc Lung Dis. 2003;7:10:959-966.

57. MacPherson DW, Gushulak BD. Balancing prevention and screening among international migrants with tuberculosis: Population mobility as the major epidemiological influence in low-incidence nations. Public Health. 2006;120:712-723. doi: 10.1016/j.puhe.2006.05.002.

58. Lönnroth K, Migliori GB, Abubakar I, D'Ambrosio L, de Vries G, Diel R, Douglas P, et al. Towards tuberculosis elimination: an action framework for low-incidence countries. Eur Respir J. 2015;45:928-952. doi: 10.1183/09031936.00214014.

59. Mayo K, White S, Oates SK, Franklin F. Community collaboration: prevention and control of tuberculosis in a homeless shelter. Public Health Nurs. 1997;13:2:120-127.

60. Tardin A, Dominicé Dao M, Ninet B, Janssens J-P. Tuberculosis cluster in an immigrant community: case identification issues and a transcultural perspective. Trop Med Int Health. 2009;14:9:995-1002. doi: 10.1111/j.1365-3156.2009.02325.x.

61. World Health Organization. Use of high burden country lists for TB by WHO in the post-2015 era. Switzerland, Geneva: World Health Organization; 2015.

62. World Health Organization. Towards TB elimination: an action framework for low-incidence countries. WHO/ HTM/TB/2014.13. Switzerland, Geneva: World Health Organization; 2014.

For peer review only

For peer review only - http://bmjopen.bmj.com/site/about/guidelines.xhtml

63. Miller CR, Mitchell EMH, Nishikiori N, Zwerling A, Lönnroth K. A web-based tool for prioritizing risk groups and selecting algorithms for screening for active tuberculosis. (Submitted)

64. Stop TB Partnership. http://www.stoptb.org/ (2019). Accessed 25 Mar 2019.

65. Khan K, Hirji MM, Miniota J, Hu W, Wang J, Gardam M, Rawal S, et al. Domestic impact of tuberculosis screening among new immigrants to Ontario, Canada. CMAJ. 2015;187:16:e473-E481.2015. doi: 10.1503/cmaj.150317.

66. Adejumo AO, Azuogu B, Okorie O, Lawal OM, Onazi OJ, Gidado M, Daniel M, Okeibunor JC, Klinkenberg E, Mitchell EMH. Community referral for presumptive TB in Nigeria: a comparison of four models of active case finding. BMC Public Health. 2016;16:177. doi: 10.1186/s12889-016-2769-7.

67. Ayles H, Muyoyeta M, Du Toit E, Schaap A, Floyd S, Simwinga M, Shanaube K, et al. Effect of household and community interventions on the burden of tuberculosis in southern Africa: the ZAMSTAR community-randomised trial. Lancet. 2013;382:1183-94. doi: 10.1016/ S0140-6736(13)61131-9.

68. Azman AS, Golub JE, Dowdy DW. How much is tuberculosis screening worth? Estimating the value of active case finding for tuberculosis in South Africa, China, and India. BMC Med. 2014;12:216. doi: 10.1186/s12916-014-0216-0.

69. Bell TR, Molinari NAM, Blumensaadt S, Selent MU, Arbisi M, Shah N, Christiansen D, et al. Impact of port of entry referrals on initiation of follow-up evaluations for immigrants with suspected tuberculosis: Illinois. J Immigr Minor Health. 2013;15:673-679. doi: 10.1007/s10903-013-9779-7.

70. Whitehead M, Dahlgren G. Concepts and principles for tackling social inequities in health: Levelling up Part 1. World Health Organization Collaborating Centre for

For peer review only - http://bmjopen.bmj.com/site/about/guidelines.xhtml

Policy Research on Social Determinants of Health, University of Liverpool. Copenhagen: World Health Organization Regional Office for Europe; 2006.

71. Wright CW. An opinion survey of tuberculosis case-finding procedures. S Afr Med J. 1979;56:6:224-8.

72. Layton MC, Cantwell MF, Dorsinville GJ, Valway SE, Onorato IM, Frieden TR. Tuberculosis screening among homeless persons with AIDS living in single-room-occupancy hotels. Am J Public Health. 1995;85:11:1555-1559.

73. Erkens C, Slump E, Kamphorst M, Keizer S, van Gerven PJHJ, Bwire R, Berkel M, et al. Coverage and yield of entry and follow-up screening for tuberculosis among new immigrants. Eur Respir J. 2008;32:153-161. doi: 10.1183/09031936.00137907.

74. Hogan H, Coker R, Gordon A, Meltzer M, Pickles H. Screening of new entrants for tuberculosis: responses to port notifications. J Public Health. 2005;27:2:192-195. doi: 10.1093/pubmed/fdi019.

75. Klinkenberg E, Manissero D, Semenza JC, Verver S. Migrant tuberculosis screening in the EU/EEA: yield, coverage and limitations. Eur Respir J. 2009;34:1180-1189. doi: 10.1183/09031936.00038009.

76. Tan L, Altman RD, Nielsen NH. Screening nonimmigrant visitors to the United States for Tuberculosis. Arch Intern Med. 2001;161:334-340. doi: 10.1080/07370016.2013.778723.

77. Akkerman OW, de Lange WCM, Schölvinck EH, Wolters B, Aartsma Y, van der Werf TS, van Hest R. Implementing tuberculosis entry screening for asylum seekers: the Groningen experience. Eur Respir J. 2016;48:261-264. doi: 10.1183/13993003.00112-2016.

78. Alvarez GG, Gushulak B, Rumman KB, Altpeter E, Chemtob D, Douglas P, et al. A comparative examination of tuberculosis immigrant medical screening programs from

For peer review only

selected countries with high immigration and low tuberculosis incidence rates. BMC Infect Dis. 2011;11:3. doi: 10.1186/1471-2334-11-3.

79. Bloss E, Newbill K, Peto H, Rice MJ, Ainsowrth G, Travnicek R, Holcombe M, et al. Challenges and opportunities in a tuberculosis outbreak investigation in Southern Mississippi, 2005-2007. South Med J. 2011;104:11. doi: 10.1097/SMJ.0b0.3e318232679e.

80. Corbett EL, Bandason T, Duong T, Dauya E, Makamure B, Churchyard GJ, Williams BG, et al. Comparison of two active case-finding strategies for community-based diagnosis of symptomatic smear-positive tuberculosis and control of infectious tuberculosis in Harare, Zimbabwe (DETECTB): a cluster-randomised trial. Lancet. 2010;376:1244-53. doi: 10.1016/S0140- 6736(10)61425-0.

81. Mhimbira FA, Cuevas LE, Dacombe R,Mkopi A, Sinclair D. Interventions to increase tuberculosis case detection at primary healthcare or community-level services. Cochrane Database Syst Rev. 2017;11:CD011432. doi: 10.1002/14651858.CD011432.pub2.

82. Fox GJ, Nhung NV, Sy DN, Hoa NLP, Anh LTN, Anh NT, Hoa NB, et al. Household-contact investigation for detection of tuberculosis in Vietnam. N Engl J Med. 2018;378:221-9. doi: 10.1056/NEJMoa1700209.

83. Fox GJ, Barry SE, Britton WJ, Marks GB. Contact investigation for tuberculosis: a systematic review and meta-analysis. Eur Respir J. 2013;41:140-156. doi: 10.1183/09031936.00070812.

84. Honarvar B, Odoomi N, Rezaei A, Haghighi HB, Karimi M, Hosseini A, Mazarei S, et al. Pulmonary tuberculosis in migratory nomadic populations: the missing link in Iran's National Tuberculosis Programme. Int J Tuberc Lung Dis. 2014;18:3:272-276. doi: 10.5588/ijtld.13.0650.

For peer review only

907 85. Kranzer K, Houben RMG, Glynn JR, Bekker L-G, Wood R, Lawn SD. Yield of HIV-associated tuberculosis during intensified case finding in resource-limited settings: a systematic review and meta-analysis. Lancet Infect Dis. 2010;10:93-102. doi: 10.1016/S1473-3099(09)70326-3.

911 86. Lönnroth K, Mor Z, Erkens C, Bruchfeld J, Nathavitharana RR, van der Werf MJ, Lange C. Tuberculosis in migrants in low-incidence countries: epidemiology and intervention entry points. Int J Tuberc Lung Dis. 2017;21:6:624-636. doi: 10.5588/ijtld.16.0845.

915 87. Mallick G, Shewade HD, Agrawal TK, Kumar AMV, Chadha SS. Enhanced tuberculosis case finding through advocacy and sensitisation meetings in prisons of Central India. Public Health Action. 2017;7:1:67-70. doi: 10.5588/pha.16.0109.

918 88. Mulder C, Erkens CGM, Kouw PM, Huisman EM, Meijer-Veldman W, Borgdorff MW, van Leth F. Missed opportunities in tuberculosis control in The Netherlands due to prioritization of contact investigations. Eur J Public Health. 2011;22:2:177-182. doi: 10.1093/eurpub/ckr017.

922 89. Mwansa-Kambafwile J, McCarthy K, Gharbaharan V, Venter FWD, Maitshotlo B, Black A. Tuberculosis Case Finding: Evaluation of a Paper Slip Method to Trace Contacts. PLoS One. 2013;89:e75757. doi: 10.1371/journal.pone.0075757.

925 90. Ospina JE, Orcau A, Millet J-P, Sánchez F, Casals M, Caylà JA. Community health workers improve contact tracing among immigrants with tuberculosis in Barcelonaa. BMC Public Health. 2012;12:158. doi: 10.1186/1471-2458-12-158.

928 91. Smit GSA, Apers L, Arrazola de Onate W, Beutels P, Dorny P, Forier A-M, et al. Cost-effectiveness of screening for active cases of tuberculosis in Flanders, Belgium. Bull World Health Organ. 2017;95:1:27-35. doi: 10.2471/BLT.16.169383.

For peer review only - http://bmjopen.bmj.com/site/about/guidelines.xhtml

92. Szkwarko D, Mercer T, Kimani S, Braitstein P, Buziba N, Carter EJ. Implementing intensified tuberculosis case-finding among street-connected youth and young adults in Kenya. Public Health Action. 2016;6:2. doi: 10.1371/journal.pone.0182185.

93. Shrivastava SR, Shrivastava PS. Tuberculosis: Active Case Finding Survey in an Urban area of India, in 2012s. J Res Health Sci. 2013;13:1:19-23.

Tables

Table 1 Characteristics of the articles included in the scoping review (n=73)

**Study characteristics**	**References**	**Number (%)**
**Year of publication**		
1979	71	1 (1%)
1995-1999	52,53,59,72	4 (5%)
2000-2009	16,23,25,26,31,36,46,47,48,56,57,60,73,74,75,76	16 (22%)
2010-2018	14,15,17,18,19,20,24,27,28,29,30,32,33,34,35,37,38, 39,40,42,44,45,49,50,51,54,55,58,65,66,67,68,69,77, 78,79,80,81,82,83,84,85,86,87,88,89,90,91,92	49 (67%)
**Region**		
Africa	19,24,29,33,38,39,44,45,54,66,67,68,71,80,89,92	16 (22%)
Americas (North America)	25,26,40,43,59,65,69,72,76,79	10 (14%)
Eastern Mediterranean	84	1 (1%)
Europe	31,34,41,46,47,48,53,55,56,60,73,74,75,77,88,90,91	17 (23%)
South-East Asia	50,52,68,87,93	5 (7%)
Western Pacific	14,15,35,36,37,51,68,82,83	9 (12%)
Global perspective	18,23,30,42,81,83,85	7 (10%)

For peer review only

Other*	16,32,49,78	4 (5%)
**Study design**		
Quantitative	14,15,20,32,33,36,37,38,44,48,49,50,51,52,54,60,65, 67,68,69,71,72,73,74,80,82,84,90,91,93	30 (41%)
Review	16,17,18,19,27,30,43,46,49,57,75,76,81,83,85,86	16 (22%)
Qualitative	24,25,29,34,41,55,56,66,78	9 (12%)
Descriptive	31,40,78,88,92	5 (7%)
Case study	28,59,79	3 (4%)
Mixed methods	35,39	2 (3%)
Other**	42,47,53,77	4 (5%)
**Target group**		
Contacts	14,20,23,24,26,29,30,39,40,44,51,67,81,82,83,88,89,91	18 (25%)
Migrants	19,31,34,41,43,46,47,48,49,55,56,57,60,65,69,73,74, 75,78,80,84,86,90	23 (32%)
Community	16,28,32,33,36,37,45,52,66,68	10 (14%)
High risk groups	18,27,42,58,71	5 (7%)
Homeless	25,53,59,72	4 (5%)
Asylum seekers or refugees	50,77	2 (3%)
People living with HIV	17,85	2 (3%)
Urban poor	15,35	2 (3%)
Other***	38,76,79,87,92,93	6 (8%)

**High HIV prevalence countries [16], high TB incidence countries [32], low-burden countries [49,78]*

***Letter to editor [77], observational study [53], perspective [42], editorial [47]*

****Population affected by TB outbreak [79], pastoralists [38], health care workers [54], prisoners [87], people living in slums [93], street connected youth and young adults [92], non-immigrant visitors [76]*

For peer review only - http://bmjopen.bmj.com/site/about/guidelines.xhtml

Table 2 Barriers and facilitators for active case-finding policy implementation (n=43)

Reported factors	References	Number (%)
Health system level		
Limited financial resources	15,23,24,28,30,35,37,40,41,46,48,52,56,71,85	15 (35%)
Existing systems and structures	18,23,29,30,34,41,44,53,56,75,85	11 (26%)
Availability of diagnostic tests and services	15,18,30,38,43,49,50,71	8 (19%)
Staff experience, expertise, motivation	3,16,22,26,28,29,30,35,37,38,41,46,55,59	14 (33%)
Over-burdening staff	15,23,28,35,38,52,76	7 (16%)
Using person-centred approaches	24,25,29,30,41,43,53,54	8 (19%)
Collaboration and integration	15,17,29,35,41,43,77	7 (16%)
Health education	37,38,39,41,43	5 (12%)
Health information and research system level		
Data, systems, supervision	18,26,30,31,32,39,48	7 (16%)
Individual and community level		
Stigma and discrimination	20,24,25,26,27,29,30,31,34,36,41,50,59,60,84,89	16 (37%)
Individual characteristics	20,25,35,36,41,43,52,54,59,71,84,90	12 (28%)
Socio-cultural factors	26,27,30,31,38,41,43,46,47,56,57,60,90,84	14 (33%)
Fear	25,26,34,35,41,43,53,55,56,57	10 (23%)
Mistrust	26,29,38,41,46,47,52,53,58,79	10 (23%)

For peer review only

For peer review only - http://bmjopen.bmj.com/site/about/guidelines.xhtml

Knowledge and awareness	20,25,26,28,29,41,53,59	8 (19%)

For peer review only

Fig 1. PRISMA flow diagram

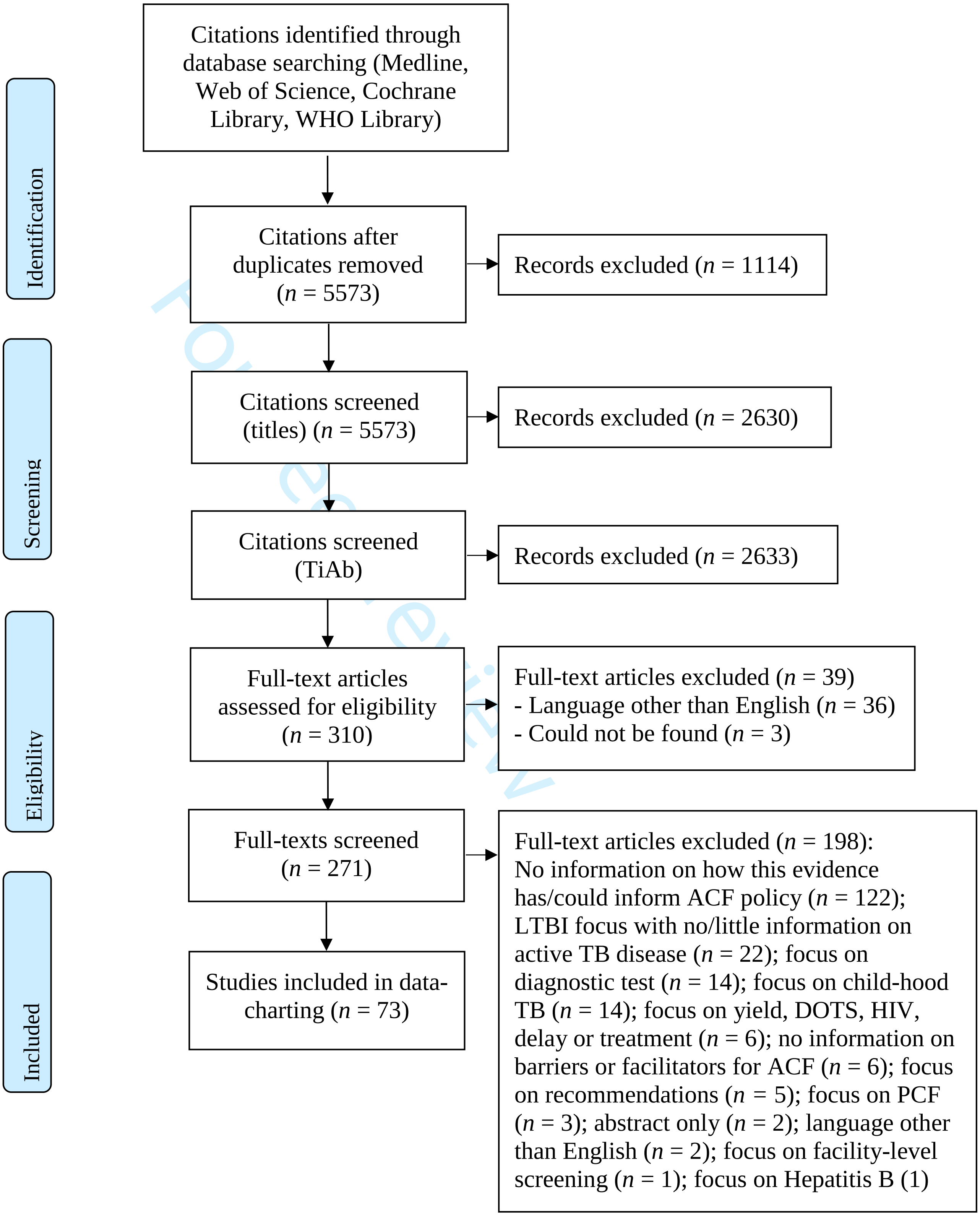

Abbreviations mentioned in Fig. 1: TiAb = Title and abstract; LTBI = Latent Tuberculosis Infection; DOTS = Directly Observed Treatment, Short Course; HIV = Human Immunodeficiency Virus; PCF = Passive Case-Finding

Preferred Reporting Items for Systematic reviews and Meta-Analyses extension for Scoping Reviews (PRISMA-ScR) Checklist

SECTION	ITEM	PRISMA-ScR CHECKLIST ITEM	REPORTED ON PAGE #
**TITLE**			
Title	1	Identify the report as a scoping review.	1
**ABSTRACT**			
Structured summary	2	Provide a structured summary that includes (as applicable): background, objectives, eligibility criteria, sources of evidence, charting methods, results, and conclusions that relate to the review questions and objectives.	2-3
**INTRODUCTION**			
Rationale	3	Describe the rationale for the review in the context of what is already known. Explain why the review questions/objectives lend themselves to a scoping review approach.	5-6
Objectives	4	Provide an explicit statement of the questions and objectives being addressed with reference to their key elements (e.g., population or participants, concepts, and context) or other relevant key elements used to conceptualize the review questions and/or objectives.	5-6
**METHODS**			
Protocol and registration	5	Indicate whether a review protocol exists; state if and where it can be accessed (e.g., a Web address); and if available, provide registration information, including the registration number.	No
Eligibility criteria	6	Specify characteristics of the sources of evidence used as eligibility criteria (e.g., years considered, language, and publication status), and provide a rationale.	7
Information sources*	7	Describe all information sources in the search (e.g., databases with dates of coverage and contact with authors to identify additional sources), as well as the date the most recent search was executed.	6
Search	8	Present the full electronic search strategy for at least 1 database, including any limits used, such that it could be repeated.	Additional file 2
Selection of sources of evidence†	9	State the process for selecting sources of evidence (i.e., screening and eligibility) included in the scoping review.	7-8
Data charting process‡	10	Describe the methods of charting data from the included sources of evidence (e.g., calibrated forms or forms that have been tested by the team before their use, and whether data charting was done independently or in duplicate) and any processes for obtaining and confirming data from investigators.	6
Data items	11	List and define all variables for which data were sought and any assumptions and simplifications made.	7-8
Critical appraisal of individual sources of evidence§	12	If done, provide a rationale for conducting a critical appraisal of included sources of evidence; describe the methods used and how this information was used in any data synthesis (if appropriate).	No

For peer review only - http://bmjopen.bmj.com/site/about/guidelines.xhtml

SECTION	ITEM	PRISMA-ScR CHECKLIST ITEM	REPORTED ON PAGE #
Synthesis of results	13	Describe the methods of handling and summarizing the data that were charted.	7-8
**RESULTS**			
Selection of sources of evidence	14	Give numbers of sources of evidence screened, assessed for eligibility, and included in the review, with reasons for exclusions at each stage, ideally using a flow diagram.	8
Characteristics of sources of evidence	15	For each source of evidence, present characteristics for which data were charted and provide the citations.	9
Critical appraisal within sources of evidence	16	If done, present data on critical appraisal of included sources of evidence (see item 12).	No
Results of individual sources of evidence	17	For each included source of evidence, present the relevant data that were charted that relate to the review questions and objectives.	Additional file 3
Synthesis of results	18	Summarize and/or present the charting results as they relate to the review questions and objectives.	10-16
**DISCUSSION**			
Summary of evidence	19	Summarize the main results (including an overview of concepts, themes, and types of evidence available), link to the review questions and objectives, and consider the relevance to key groups.	16-21
Limitations	20	Discuss the limitations of the scoping review process.	22-23
Conclusions	21	Provide a general interpretation of the results with respect to the review questions and objectives, as well as potential implications and/or next steps.	23-24
**FUNDING**			
Funding	22	a) Describe sources of funding for the included sources of evidence, as well as sources of funding for the scoping review. b) Describe the role of the funders of the scoping review.	a) No; b) 25

JBI = Joanna Briggs Institute; PRISMA-ScR = Preferred Reporting Items for Systematic reviews and Meta-Analyses extension for Scoping Reviews.

* Where *sources of evidence* (see second footnote) are compiled from, such as bibliographic databases, social media platforms, and Web sites.

† A more inclusive/heterogeneous term used to account for the different types of evidence or data sources (e.g., quantitative and/or qualitative research, expert opinion, and policy documents) that may be eligible in a scoping review as opposed to only studies. This is not to be confused with *information sources* (see first footnote).

‡ The frameworks by Arksey and O'Malley (6) and Levac and colleagues (7) and the JBI guidance (4, 5) refer to the process of data extraction in a scoping review as data charting.

§ The process of systematically examining research evidence to assess its validity, results, and relevance before using it to inform a decision. This term is used for items 12 and 19 instead of "risk of bias" (which is more applicable to systematic reviews of interventions) to include and acknowledge the various sources of evidence that may be used in a scoping review (e.g., quantitative and/or qualitative research, expert opinion, and policy document).

From: Tricco AC, Lillie E, Zarin W, O'Brien KK, Colquhoun H, Levac D, et al. PRISMA Extension for Scoping Reviews (PRISMA-ScR): Checklist and Explanation. Ann Intern Med. ;169:467–473. doi: 10.7326/M18-0850

For peer review only - http://bmjopen.bmj.com/site/about/guidelines.xhtml

Additional file 2: Literature search strategy for MEDLINE

We implemented the search strategy on 24 January 2018. The search yielded 4640 results.

1 exp Tuberculosis/ or exp Mycobacterium tuberculosis/

2 (tuberculosis or tb or pulmonary consumption or consumption, pulmonary or pulmonary phthisis or tuberculoses).ti,ab.

3 1 or 2

4 exp Mass screening/ or exp Mass chest x-ray/ or exp Contact tracing/ or exp Health surveys/ or exp Cross-sectional studies/ or exp Epidemiologic studies/ or exp Systematic review/

5 (Mass chest x ray$ or screen$ or mass screening$ or population screening$ or survey$ or screening survey$ or household survey or health survey$ or cross-sectional or detect$ or active case or case detect$ or case finding or tuberculosis case finding or active case finding or contact tracing or contact examination or contact screening or contact investigation or employment testing or intensified case finding or prevalence stud$ or prevalen* or inciden* or algorithm or undiagnosed or checking or pre-entry or passive or TB suspect$ or notification or notified).ti,ab.

6 4 or 5

7 (view$ or barrier$ or block$ or obstacle$ or hinder$ or constrain$ or facilitat$ or enabl$ or benefit$ or opportun$ or attitude$ or opinion$ or belief$ or perceiv$ or perception$ or aware$ or personal view$ or motivat$ or reason$ or incentiv$ or cost$ or cost-effective$ or cost analys$ or cost comparison or cost-minimization or cost measure$ or affordab$ or resource$ or resource allocation or resource$ or efficiency).ti,ab.

For peer review only - http://bmjopen.bmj.com/site/about/guidelines.xhtml

8 exp Attitude/ or exp Motivation/ or exp Cost/ or exp Cost analysis/ or exp Resource allocation/

9 7 or 8

10 exp Policy Making/ or exp Health Plan Implementation/ or exp Health Priorities/

11 (policy mak$ or policy develop$ or policy analys$ or health polic$ or policy implement$ or health plan implement$ or priorit$ or health priorit$).ti,ab.

12 10 or 11

13 9 or 12

14 ((((((((((((((((hard$ adj2 reach) or hard$) adj2 locate) or hard$) adj2 find) or hard$) adj2 treat) or difficult) adj2 locate) or Difficult) adj2 engage) or social$ exclu$ or social inequalit$ or difficult$) adj2 reach) or difficult$) adj2 find) or difficult$) adj2 treat).ti,ab.

15 ((geograph$ or transport$ or physical) and barrier$).ti,ab.

16 ((low$ or poor$ or negative) and (quality adj2 life)).ti,ab.

17 ((vulnerable or disadvantaged or at risk or high risk or low socioeconomic status or neglect$ or marginal$ or forgotten or non-associative or unengaged or hidden or excluded or transient or inaccessible or underserved or stigma$ or inequitable) and (people or population$ or communit$ or neighbourhood$1 or neighborhood$1 or group$ or area$1 or demograph$ or patient$ or social$)).ti,ab.

18 vulnerable populations/

19 17 or 18

For peer review only

20 poverty area/

21 (refuser$1 or non-user$1 or discriminat$ or shame or prejud$ or racism or racial discriminat$).ti,ab.

22 social support/ or *social conditions/ or stigma/ or Social Isolation/ or *quality of life/ or Prejudice/ or Socioeconomic Factors/

23 (immobile or (disabled and (house bound or home bound)) or ((house or home) and bound)).ti,ab.

24 Homebound Persons/

25 23 or 24

26 ((hous$ and (quality or damp$ or standard$ or afford$ or condition$ or dilapidat$)) or ((emergency or temporary or inadequate or poor$ or overcrowd$ or over-crowd$ or over-subscribed) and (hous$ or accommodation or shelter$ or hostel$ or dwelling$))).ti,ab.

27 housing/st

28 26 or 27

29 (rough sleep$ or runaway$1 or ((homeless$ or street or Destitut$) and (population or person$1 or people or group$ or individual$1 or shelter$ or hostel$ or accommodation$1))).ti,ab.

30 exp homeless persons/

31 29 or 30

32 ((drug$ or substance) and (illegal or misus$ or abuse or intravenous or IV or problem use$ or illicit use$ or addict$ or dependen$ or dependant or delinquency)).ti,ab.

For peer review only

33 *Substance-Related Disorders/ or Drug users/

34 32 or 33

35 ((alcohol$ and (misus$ or abuse or problem$ use$ or problem drink$ or illicit use$ or addict$ or dependen$ or dependant or delinquency)) or alcoholic$1).ti,ab.

36 *Alcohol- Related Disorders/ or Alcoholics/

37 35 or 36

38 (prostitution or sex work$ or transactional sex$ or prostitute$1).ti,ab.

39 Prostitution/

40 38 or 39

41 (poverty or deprivation or financial hardship$).ti,ab.

42 ((low-income or low income or low pay or low paid or poor or deprived or debt$ or arrear$) and (people or person$1 or population$1 or communit$ or group$ or social group$ or neighbourhood$1 or neighborhood$1 or famil$)).ti,ab.

43 poverty/

44 (low$ and social class$).ti,ab.

45 (traveller$1 or gypsies or gypsy or Romany or roma).ti,ab.

46 gypsies/

47 45 or 46

48 (mental$ and (health or ill or illness)).ti,ab.

For peer review only - http://bmjopen.bmj.com/site/about/guidelines.xhtml

49 *mental health/ or Mentally Ill Persons/

50 48 or 49

51 ((((health care worker$1 or health care) adj2 service provi$) or health-care) adj2 provi$).ti,ab.

52 (outreach adj2 worker$1).ti,ab.

53 Community health aides/

54 52 or 53

55 (support adj2 worker$1).ti,ab.

56 (case adj2 worker$1).ti,ab.

57 (social adj2 worker$1).ti,ab.

58 social care professional$1.ti,ab.

59 ((social care adj2 service provi$) or (social-care adj2 provi$)).ti,ab.

60 (((language$ or communicat$) and (barrier$ or understand$ or strateg$ or proficien$)) or translat$ or interpret$ or (cultur$ and competen$)).ti,ab.

61 Communication Barriers/ or *Language/

62 60 or 61

63 (((immigrant$ or migrant$ or asylum or refugee$ or undocumented or foreign born or born) adj overseas) or (displaced and (people or person$1))).ti,ab.

For peer review only - http://bmjopen.bmj.com/site/about/guidelines.xhtml

For peer review only

64 ((((Emigration.mp. and Immigration/) or refugees/ or Transients.mp.) and migrants/) or Emigrants.mp.) and immigrants/ [mp=title, abstract, original title, name of substance word, subject heading word, keyword heading word, protocol supplementary concept word, rare disease supplementary concept word, unique identifier, synonyms]

65 63 or 64

66 exp Diabetes Mellitus/

67 diabet$.tw,ot.

68 (IDDM or NIDDM or MODY or T1DM or T2DM or T1D or T2D).tw,ot.

69 (non insulin$ depend$ or noninsulin$ depend$ or non insulin?depend$ or noninsulin?depend$).tw,ot.

70 (insulin$ depend$ or insulin?depend$).tw,ot.

71 or/66-70

72 14 or 15 or 16 or 19 or 20 or 21 or 22 or 25 or 28 or 31 or 34 or 37 or 40 or 41 or 42 or 43 or 44 or 47 or 50 or 51 or 54 or 55 or 56 or 57 or 58 or 59 or 62 or 65 or 71

73 3 and 6 and 13 and 72

74 limit 73 to yr="1968 -Current"

75 animals/ not humans/

76 74 not 75

For peer review only

For peer review only - http://bmjopen.bmj.com/site/about/guidelines.xhtml

Additional file 3: Abridged version of the data charting table

The abridged data charting table contains information on author, year of publication and study design (column 1), the country where the study was implemented and the target population (column 2), ACF antecedents (column 3), ACF components (column 4), factors influencing ACF policy implementation at the level of the health system (column 5) and the individual and community level (column 6).

	1	2	3	4	5	6
**ID**	**Author, year, design**	**Country, target population**	**ACF antecedents**	**ACF components**	**Factors influencing ACF policy implementation:** health system context	**Factors influencing ACF policy implementation:** individual and community context
1	Abubakar, 2012, review	Low-burden, migrants			Screening site characteristics; diagnostic test availability; molecular assay costs	
2	Adejumo, 2016, qualitative	Nigeria, community			CHW age, work experience, knowledge	
3	Akkerman, 2016, letter to editor	the Netherlands, asylum-seekers			High-volume CXR readers; cooperation between public health and clinical workers	

For peer review only - http://bmjopen.bmj.com/site/about/guidelines.xhtml

4	Alvarez*, 2011, descriptive	Low-burden, migrants	despite lack of evidence, ACF is major global health priority			
5	Ayakaka, 2017, qualitative	Uganda, contacts		Education and incentivization for health workers; persuasion; enablement	CHW travel costs, pay, skills; personalized, enabling services; staff shortages; fear of TB; patient communication; language barrier; difficulty locating households; lack of ACF guidelines	Fear of stigma; discrimination; among contacts: limited knowledge, mistrust, travel costs, avoidant behavior; privacy provided by home evaluation
6	Ayles, 2013, quantitative	South Africa, Zambia, contact			Linkage and retention in care	Empowerment to seek care early
7	Azman*, 2014, quantitative	China, India, South Africa, community				
8	Baxter, 2017, review	Worldwide, contacts			Using molecular epidemiological testing; ensuring adequate systems; local expertise and capability; communication; coordination; prompt	Stigma; language barrier; high mobility

For peer review only

					action; addressing fear; error; lack of resources	
9	Bell*, 2013, quantitative	USA, migrants				
10	Bloss, 2011, case study	USA, outbreak			Institutional knowledge about TB control; limited human resources	Community trust and rapport
11	Van den Bosch, 2000, descriptive	UK, migrants		Incentivization for screening participants; health education for general public		Change of address; false address; language barrier; mistrust of authorities; fear of TB and stigma
12	Brewin, 2006, qualitative	UK, migrants			Integration of health and social services; waiting time	Feelings of reassurance, responsibility, anxiety linked to ACF; lack of public health information
13	Chemtob, 2003, qualitative	Israel, migrants			Adequate health facilities; funds for transportation; attitudes; dialogue with	Socio-cultural barriers; anti-immigrant sentiments; fear of deportation; no standard term for TB;

For peer review only - http://bmjopen.bmj.com/site/about/guidelines.xhtml

For peer review only

					immigrants and providers in planning and implementation	empowerment to assume greater responsibility for health promotion
14	Cook, 2012, descriptive	Canada, aboriginal community			Foundation of routine ACF including interview skills, dedicated resources to undertake ACF, follow-up	Community buy-in
15	Corbett, 2013, review	Africa, PLHIV	Radiological screening widely implemented before 1970s	Target group: populations with high prevalence	Sensitivity of diagnostic tests; communication and interaction with the community; transportation	
16	Corbett, 2010, quantitative	Zimbabwe, congregate setting	ACF implemented in HIC since 1920s; ACF discouraged due to costs and weak treatment programmes			
17	Datiko, 2017, quantitative	Ethiopia, community		Linkage to laboratory services and treatment		

For peer review only - http://bmjopen.bmj.com/site/about/guidelines.xhtml

				through a transport network		
18	de Vries, 2017, review	Worldwide, high-risk		Awareness; cultural sensitivity; access to high-quality translation services; cross-cultural training of providers	Structural barriers; cultural and language barriers	Low perceived susceptibility; stigma and its social consequences
19	Dierberg, 2016, quantitative	India, refugees			Use of Xpert MTB/RIF; available time and human resources; lack of a laboratory technician; trained providers	High mobility; awareness; stigma
20	Eang, 2012, quantitative	Cambodia, contacts	Growing interest in ACF in LMIC			
21	Erkens*, 2008, quantitative	the Netherlands, migrants				
22	Mhimbira, 2017, review	Worldwide, contacts		Health promotion activities;		

For peer review only - http://bmjopen.bmj.com/site/about/guidelines.xhtml

				education for health workers		
23	Fox, 2018, quantitative	Vietnam, contacts	ACF in high-prevalence settings remains limited			
24	Fox, 2013, review	Worldwide, contacts, congregate setting	Growing interest ACF in LMICs			
25	Fox, 2015, quantitative	Vietnam, contacts	WHO recommendations for ACF		Proximity of services	Discrimination; lack of knowledge; misconceptions; attitudes and (traditional) beliefs; difficulty remembering appointments
26	Getnet, 2017, quantitative	Ethiopia, pastoralists			Proximity of services; diagnostic algorithm and tools used; quality of diagnostic services; provider expertise; burden on CHWs; health education for TB patients	Health seeking behavior; lack of confidence in CHWs; personal or cultural beliefs; gender norms

For peer review only - http://bmjopen.bmj.com/site/about/guidelines.xhtml

27	Godfrey-Faussett, 2003, review	Worldwide, high HIV prevalence setting	Movement towards decentralized diagnostic centers		Fear; stigma	
28	Golub, 2005, review	Worldwide, contacts	Mass radiography as ACF strategy in HICs before 1960s; WHO recommendation against indiscriminate mass screening (1974); exploration of new ACF strategies; recognized global urgency		Workload and cost; method of case detection; training of laboratory technicians, clinicians and field staff; appropriate bacteriological examination; laboratory network	
29	O'Hara, 2015, quantitative	South Africa, health care workers			Cost and confidentiality of services; waiting times	Sex, occupation, time since a previous TB test

For peer review only - http://bmjopen.bmj.com/site/about/guidelines.xhtml

30	Harper, 1996, quantitative	Nepal, remote community			Cost; lack of staff and funding; absenteeism; use of 'alternative' services; accessibility of services	Low confidence amongst users and general under-usage of services
31	Hinderaker, 2011, quantitative	Low-income, low-income community	Testing of different ACF approaches since 1960s, WHO targets set 2005	Social mobilization; health education for general public; communication; private sector engagement; health systems strengthening; incentivization for health workers and TB patients	Regular supervision; availability of district laboratory supervisor	
32	Hogan, 2005, quantitative	UK, migrants			Service capacity; lack of national guidance; inconsistency in implementing ACF	

For peer review only - http://bmjopen.bmj.com/site/about/guidelines.xhtml

33	Honarvar, 2014, quantitative	Iran, migrants				Stigma; high mobility; poverty; low literacy; tribal behaviors; traditional beliefs; gender norms; language barrier; self-discrimination
34	Karki, 2017, quantitative	Papua New Guinea, community	Role of ACF uncertain since 1970s; WHO recommendation against indiscriminate mass screening (1974)		Poor health services; lack of outreach services; limited funds at district level; cost; availability of staff and the payment of allowances; staff skills and experience; health education for general public	
35	Kerrigan, 2017, qualitative	South Africa, contacts	Despite lack of evidence, ACF is major global health priority		Provider training, safety; incentivization for screening participants; lack of resources, logistics; involvement of culturally respected, valued stakeholder/organization venues; community-based mobilization and events	Stigma; preference of different screening locations; travel time and costs; gossip

For peer review only

36	Khan*, 2015, quantitative	Canada, migrants				
37	Klinkenberg, 2009, review	EU, migrants			Access to healthcare for migrants; availability of a good follow-up system; integration with healthcare	
38	Kranzer, 2010, review	Worldwide, PLHIV	Mass radiography in Czechoslovakia 1961-72 showing decrease in prevalence	Target population; screening strategy; laboratory capacity; operational feasibility; costs	Feasibility and costs; laboratory capacity	
39	Kranzer, 2012, review	South Africa, deprived community		Incentivization for screening participants		Waiting time; ability to produce a sputum sample; health status
40	Kulane, 2010, qualitative	Sweden, Somali community				Stigma; migration; migration policies; fear of deportation; misconceptions
41	Layton, 1995, review	USA, homeless			Incentivization for screening participants	

For peer review only - http://bmjopen.bmj.com/site/about/guidelines.xhtml

For peer review only

42	Li, 2017, case study	China, community			Heavy workload; inadequate providers; time and human resource constraints; provider education and competence	Acceptance; knowledge; awareness; health status; incentivization for screening participants; health education for TB patients
43	Lorent, 2015, mixed methods	Cambodia, urban poor		Health education for general public; community involvement; peer support networks	Challenging working conditions; collaboration between TB workers and CHWs; CHW compensation, familiarity, knowledge of community, remuneration; provider motivation; financial support	Acceptance; cost, fear; embarrassment; preference of different screening locations; privacy concerns; involvement of CHWs; non-acceptance of diagnosis
44	Lorent, 2014, quantitative	Cambodia, urban poor		Flexible patient-centered treatment approach	Logistics; finances; heavy workload; CHW integration, network, recognition, remuneration; use of mobile phone, collaboration with providers and laboratory technicians; infrastructure	
45	Lönnroth, 2013, review	Worldwide, high-risk	WHO recommendation against indiscriminate mass screening (1974)	Confidentiality; access to free, good quality services;	Available, high-quality services; NTP performance; health system capacity; public health law; existing platforms for	.;

For peer review only - http://bmjopen.bmj.com/site/about/guidelines.xhtml

				provider training; minimization of initial loss to follow-up; enforced notification; engagement of public/private providers; resources	outreach and health promotion activities, health and social services	
46	Lönnroth, 2017, review	Low incidence, migrants		Target groups; screening algorithms; timing; values; preferences; competing health care needs; weak evidence base		
47	Lönnroth, 2015, descriptive	Low-incidence, high-risk				Trust

For peer review only - http://bmjopen.bmj.com/site/about/guidelines.xhtml

48	MacPherson, 2006, review	Low-incidence, migrants				Fear; language barrier; culture; social factors
49	Mallick*, 2017, descriptive	India, prisoners				
50	Mayo, 1996, case study	USA, homeless			ACF location; incentivization for TB patients; providers' frustration; involvement of clients	Stigma; substance use; poor social skills; health status; poor access to services and the necessities of life; unemployment; homelessness
51	Mor, 2008, quantitative	Israel, migrants		Unification of medical procedures	Operational cost; infrastructure	
52	Morishita, 2016, quantitative	Cambodia, contacts				Cost; insurance
53	Mulder, 2011, descriptive	the Netherlands, contacts			Limited health system capacity to keep track of immigrant index cases	

For peer review only - http://bmjopen.bmj.com/site/about/guidelines.xhtml

54	Mwansa-Kambafwile, 2013, quantitative	South Africa, contacts				Discomfort linked to home visits; confidentiality; stigma; discrimination
55	Ospina, 2012, quantitative	Spain, migrants		Involvement of CHWs	CHW involvement; language barrier	Birthplace; unknown district of residence; HIV infection; homelessness; sex; incarceration history; language and cultural barrier
56	Seedat, 2014, qualitative	UK, migrants		Community, proactive services; collaboration with community organizations; combining diseases into health check-up; awareness raising	Lack of capacity, funding, advocacy, psycho-social support services, awareness; link with community organizations; cultural insensitivity; time and distance to services	Stigma; culture; gender norms; faith; language barrier; misconceptions; fear of lack of confidentiality, cost, disease status; lack of awareness, knowledge, confidence

For peer review only - http://bmjopen.bmj.com/site/about/guidelines.xhtml

57	Sekandi, 2015, quantitative	Uganda, contacts	ACF currently used in research settings		Lack of well-organized public health systems; staff shortage; limited resources for personnel time, administration and transportation	
58	Shrestha-Kuwahara, 2003, qualitative	USA, contacts			Lack of clear definitions; challenge of communicating the need to obtain personal information; limited knowledge on socio-economic issues among staff; training of, frustration among providers	Trust; understanding; knowledge; feeling moral obligations to family/friends confusion; fear of alienation, abandonment, stigma, loss of employment or housing; negative perception of interview process; misconceptions; language and cultural barriers
59	Shrivastava, 2013, quantitative	India, slums	Periodic ACF remains an integral part of TB control in high-risk groups	Involvement of CHW		
60	Smit*, 2017, quantitative	Belgium, high-risk, contacts				

61	Southern, 1999, observational	UK, homeless		Immediate examination of CXR; fast-track referral system	Non-judgmental, flexible, supportive approach	Lack of interest; fear of the outcome; waiting times; health education of screening participants
62	Suthar, 2016, perspective	Worldwide, high-risk	2030 goals important for inspiring and persuading communities to collaborate and step up TB response			
63	Swigart, 2004, qualitative	USA, homeless				Health status; knowledge about TB; costs; fear; disinterest
64	Szkwarko, 2016, descriptive	Kenya, street-connected youth and young adults			Trust; difficulties obtaining morning sputum; underrepresentation of women	

For peer review only - http://bmjopen.bmj.com/site/about/guidelines.xhtml

65	Tan, 2001, review	USA, nonimmigrant visitors			Funding and logistical difficulties; technical issues; overburdening providers	
66	Tankimovich, 2013, review	USA, homeless, migrants		Coordination and collaboration of programmes	Education and incentivization for screening participants and TB patients; accessible, affordable facilities and technology; cultural knowledge; coordination between governments, academic and private institutions	Lack of knowledge; misconceptions; contact difficulties; co-morbidities; cultural values
67	Tardin, 2009, quantitative	Switzerland, migrants				Stigma; different communication styles and health beliefs; language barrier; acceptance of diagnosis; respect towards authority; religious value; tight community network
68	Tupasi, 2000, quantitative	Philippines, community			Services free and conveniently located	Symptoms; fear of stigma

For peer review only - http://bmjopen.bmj.com/site/about/guidelines.xhtml

69	Welshman, 2006, review	UK, migrants			Dissatisfaction with work; disjunction between guidelines and practice; cost; logistics; administration; language barrier; shortage of radiologists/ radiographers	Changes of address; language barrier; mistrust of authorities
70	Welshman, 2006, editorial	UK, migrants			Logistics; administration; language barrier; shortages of radiologists/radiographers	Changes of address; language barrier; mistrust of authorities
71	Wright, 1979, quantitative	South Africa, high-risk			Limited and inadequate facilities for the examination of sputum; cost	"Degree of sophistication of the people involved"
72	Yassin, 2013, mixed methods	Ethiopia, contacts		Capacity strengthening; advocacy; communication; social mobilization; transport; treatment; CHW training,	Providers' commitment; CHWs' job satisfaction; laboratory technicians' perceived benefit of ACF; burden on laboratory; incentivization of laboratory technicians; benefit from new equipment; quality of smears prepared by CHWs	

For peer review only - http://bmjopen.bmj.com/site/about/guidelines.xhtml

				supervision, support		
73	Zenner, 2017, review	Worldwide, migrants	WHO End TB strategy; WHO against indiscriminate mass screening (1974)		Type of programme; target group; voluntary vs mandatory ACF	

* The article contains important information on additional influencing factors beyond the ones displayed in this table. Due to space limitations, we did not include them here.

For peer review only - http://bmjopen.bmj.com/site/about/guidelines.xhtml

BMJ Open

Factors influencing active tuberculosis case finding policy development and implementation: a scoping review

Journal:	*BMJ Open*
Manuscript ID	bmjopen-2019-031284.R2
Article Type:	Original research
Date Submitted by the Author:	04-Nov-2019
Complete List of Authors:	Biermann, Olivia; Karolinska Institutet, Public Health Sciences<br>Lönnroth, Knut; Karolinska Institutet, Public Health Sciences<br>Caws, Maxine; Liverpool School of Tropical Medicine, Department of Clinical Sciences; Birat Nepal Medical Trust<br>Viney, Kerri ; Karolinska Institutet, Public Health Sciences; Australian National University, Research School of Population Health, College of Health and Medicine
**Primary Subject Heading**:	Public health
Secondary Subject Heading:	Global health, Health policy
Keywords:	Tuberculosis < INFECTIOUS DISEASES, active case-finding, Health policy < HEALTH SERVICES ADMINISTRATION & MANAGEMENT, policy development, policy implementation, PREVENTIVE MEDICINE

SCHOLARONE™
Manuscripts

Factors influencing active tuberculosis case finding policy development and implementation: a scoping review

Olivia Biermann[1]* (olivia.biermann@ki.se)

Knut Lönnroth[1] (knut.lonnroth@ki.se)

Maxine Caws[2 3] (maxine.caws@lstmed.ac.uk)

Kerri Viney[1 4] (kerri.viney@anu.edu.au)

1 Department of Public Health Sciences, Karolinska Institutet, Tomtebodavägen 18a, 17177 Stockholm, Sweden

2 Department of Clinical Sciences, Liverpool School of Tropical Medicine, Pembroke Pl, Liverpool L3 5QA, United Kingdom

3 Birat Nepal Medical Trust, Lazimpat, Kathmandu, Nepal

4 Research School of Population Health, College of Health and Medicine, Australian National University, Canberra ACT 2600, Australia

*Corresponding author

For peer review only - http://bmjopen.bmj.com/site/about/guidelines.xhtml

Abstract (300 words)

Objective

To explore antecedents, components and influencing factors on ACF policy development and implementation.

Design

Scoping review, searching MEDLINE, Web of Science, the Cochrane Database of Systematic Reviews and the WHO Library from January 1968 to January 2018. We excluded studies focusing on latent TB infection, passive case-finding, childhood TB and studies about effectiveness, yield, accuracy and impact without descriptions of how this evidence has/could influence ACF policy or implementation. We included any type of study written in English, and conducted frequency and thematic analyses.

Results

Seventy-three articles fulfilled our eligibility criteria. Most (67%) were published after 2010. The studies were conducted in all WHO regions, but primarily in Africa (22%), Europe (23%) and the Western-Pacific region (12%). Forty-one percent of the studies were classified as quantitative, followed by reviews (22%) and qualitative studies (12%). Most articles focused on ACF for TB contacts (25%) or migrants (32%). Fourteen percent of the articles described community-based screening of high-risk populations. Fifty-nine percent of studies reported influencing factors for ACF implementation; mostly linked to the health system (e.g. resources) and the community/individual (e.g. social determinants of health). Only two articles highlighted factors influencing ACF policy development (e.g. politics). Six articles described WHO's ACF-related recommendations as important antecedent for ACF. Key components of successful ACF implementation include health system capacity, mechanisms for integration, education and collaboration for ACF.

For peer review only

Conclusion

We identified some main themes regarding the antecedents, components and influencing factors for ACF policy development and implementation. While we know much about facilitators and barriers for ACF policy implementation, we know less about *how* to strengthen those facilitators and *how* to overcome those barriers. A major knowledge gap remains when it comes to understanding which contextual factors influence ACF policy development. Research is required to understand, inform and improve ACF policy development and implementation.

Key words

Tuberculosis, active case-finding, community, policy development, policy implementation

Article summary

Strengths and limitations of this study

- The scoping review design was able to provide collated and comprehensive insights into the peer-reviewed scientific literature on the topic, and included a wide scope of factors influencing ACF policy development and implementation.
- The review may have provided a more in-depth insight into the topic by including additional databases, searching grey literature and references of included studies, and contacting authors for more information.
- We did not do a critical appraisal of the individual sources of evidence or within sources of evidence, nor did we describe sources of funding for the included sources of evidence.
- We limited the inclusion to studies written in English.

For peer review only

For peer review only - http://bmjopen.bmj.com/site/about/guidelines.xhtml

- Reporting of ACF policy development and implementation varied in completeness across the included articles, and as such, our data are limited by the details described in the literature.

Manuscript (Word count: 5567 words)

Background

Systematic screening for tuberculosis (TB) is defined by the World Health Organization (WHO) as the "systematic identification of people with suspected active tuberculosis (TB), in a predetermined target group, using tests, examinations or other procedures that can be applied rapidly" [1]. Active case-finding (ACF) is synonymous with systematic screening for active TB, although it usually implies screening outside of health facilities.

Tuberculosis is a major global health challenge, particularly in low- and middle-income countries. The WHO End TB Strategy and the Sustainable Development Goals aim at finding the "missing cases" and ending the global TB epidemic by 2030. This will require intensified activity to increase TB case detection, specifically in "hard-to-reach" groups [2]. In 2019, there was a 3 million gap between estimated incident and notified TB cases globally, reflecting a combination of an underdiagnosis of cases and underreporting of cases who have been detected [3]. Many people with TB are diagnosed only after long delays [4], causing suffering and economic hardship for TB patients and TB-affected households, and sustained transmission [1].

For peer review only - http://bmjopen.bmj.com/site/about/guidelines.xhtml

Active case finding is mostly provider-initiated. It targets people in high-risk groups who may not seek health care actively [1]. Potential benefits for patients include reduced morbidity, mortality and economic consequences due to earlier diagnosis, while society can benefit from reduced transmission and a reduced burden of TB, which often affects the most economically productive members of a society. TB screening in high-risk groups has been implemented in many settings within the TB program or in the context of research and can significantly improve TB case notification [5-7].

However, if not well targeted and implemented, ACF can be costly leading to diversion and waste of scarce resources, potentially compromising passive case-finding infrastructure and weakening health systems. It can also cause harm to individuals, e.g. by increasing the risk of a false positive diagnosis and providing TB treatment to individuals without TB, or increased stigma and discrimination arising from uncovering TB, which need to be weighed against the benefit of identifying cases in the community that would otherwise go untreated [1,2].

Questions remain about both *if* ACF in general is worthwhile, and *how* to best plan and implement outreach screening through ACF in a given context as a synergistic way, i.e. integrated into the health care system, and not delivered separately in a parallel system. The evidence base is weak concerning the benefits and cost-effectiveness of ACF and how it varies between risk groups [8]. The strength of evidence to support alternative ACF approaches is low, with few head-to-head trial results being available to inform practice. Many differences exist in stakeholder's values and preferences concerning the rationale, outcomes and possible secondary effects of ACF. Despite the relatively weak evidence, WHO has a guideline on systematic screening and the Global Fund [9] and TB REACH [10] provide increasing funding for ACF, while a growing number of countries are increasing investment

For peer review only - http://bmjopen.bmj.com/site/about/guidelines.xhtml

in and have national policies for ACF. For example, Vietnam's National Strategic Plan 2015-2020 includes ACF in remote and congregate settings, and in high-risk groups including contacts, prisoners, miners, the elderly, homeless people, young adults, migrants, factory workers, communities, people living with Human Immunodeficiency Virus (HIV), multidrug-resistant patients, young males, smokers and intravenous drug users [11].

The aim of this study was to explore antecedents, components and influencing factors for ACF policy development and implementation, to inform and improve future ACF policy processes. We did not aim to review the evidence on ACF per se; therefore, we explore yield, accuracy and impact only in the context of how they influence ACF policy development or implementation.

Methods

We conducted a scoping review, using an a-priori protocol based on the following research question: *Which are the antecedents, components and influencing factors (barriers and facilitators) in developing and implementing ACF policies?* The scoping review methodology was deemed appropriate given the breadth of the question. The protocol was based on guidance from the Joanna Briggs Methods Manual for Scoping Reviews [12]. The inclusion criteria, the screening manual and the data-charting table were developed by one author (OB), reviewed by two authors (KV and KL) to ensure face validity, and pilot-tested. All team members were consulted at various stages of the scoping review to provide input on the search, data extraction and charting, and the interpretation of the results. The charted data was summarized in tables, and then condensed resulting in Tables 1 and 2. The Preferred Reporting Items for Systematic reviews and Meta-Analyses Extension for Scoping Reviews

For peer review only - http://bmjopen.bmj.com/site/about/guidelines.xhtml

(PRISMA-ScR) was used to guide reporting [13]. The completed checklist is available in *Additional file 1*. The protocol is available from the corresponding author upon request.

Data sources and search

We searched MEDLINE, Web of Science, The Cochrane Database of Systematic Reviews and the WHO Library from January 1968 (year of the first publication on ACF in MEDLINE) until January 2018. The search strategy was developed in collaboration with a medical librarian from Karolinska Institutet (Carl Gornitzki), and further refined through team discussion. The search strategy for MEDLINE is available in *Additional file 2*. The latter was standardized but adapted to fit the other database searches. The respective search strategies are available from the corresponding author upon request. The results of the literature search were imported into Rayyan.

Inclusion criteria

Eligible studies focused on the detection of active TB disease. The review included studies describing or analysing ACF policy development and implementation. Facilitators and barriers linked to access or treatment were included, if there was a clear link to ACF. Furthermore, the review included studies analysing the use of evidence in ACF policy development and implementation. Studies of any design, conducted in any setting or country, and those published in the English language were eligible for inclusion. We excluded studies focusing only on latent TB infection, passive case-finding and childhood TB, and studies about effectiveness, yield, accuracy and impact without descriptions of how this evidence has/could influence ACF policy. The inclusion of different ACF approaches implies that while many lessons can be learned across different settings, some might not be generalizable.

For peer review only - http://bmjopen.bmj.com/site/about/guidelines.xhtml

Screening, data abstraction and charting

One reviewer (OB) initially reviewed titles to remove duplicates, and those studies not focusing on TB. Two reviewers (OB and KV) independently reviewed titles and abstracts, and then full-text articles for inclusion. Conflicts were resolved through discussion, based on the inclusion/exclusion criteria.

The data-charting table was developed in Microsoft Excel. An abridged version of the table is available in *Additional file 3*. Data was extracted on study design, country, target population for ACF, benefits of ACF, risks of ACF, ACF antecedents, ACF policy development and implementation (use of evidence, country-level to individual-level facilitators and barriers, stakeholders involved), lessons learned, future perspectives and future research. Relevant data was charted by one author (OB), while a second author (KV) verified this step by charting data from a random sample of studies for comparison. Differences in data-charting were resolved by discussion.

Patient and public involvement

This study is related to a qualitative study of experts and a survey of National TB Programme managers combined with a policy document review on the topic of ACF policy development and implementation which are currently under way to harness tacit knowledge on the topic. The research questions and variables for these studies were informed by the results of this scoping review. The results of this study will be presented to researchers and policy-makers in the field via targeted issue briefs. We will also share the results with the public via a video and short messages on social media.

Results

For peer review only - http://bmjopen.bmj.com/site/about/guidelines.xhtml

Literature search

After screening 2943 titles and abstracts and 271 full-text articles, 73 unique articles fulfilled our eligibility criteria (*Figure 1*) [14-86]. The reasons for excluding full-text articles are provided in Fig. 1. What all included studies have in common is the goal of finding ‘missing’ TB cases through ACF, while the approaches vary greatly; from contact investigation to screening people in homeless shelters (Table 1). An example of a study included in this review is a qualitative study by Ayakaka et al., which explored influencing factors for TB contact investigation in Kampala, Uganda [14]. The stakeholders who were interviewed described key barriers for ACF as being limited knowledge about TB among contacts, stigma, mistrust of health centre staff among index patients and contacts, and high travel costs for lay health workers and contacts. At the same time, key facilitators for ACF comprised personalized and enabling services provided by lay health workers. To overcome barriers and strengthen facilitators, the researchers identified education, incentivization and restructuring of the service environment as relevant interventions [14].

Characteristics of the included articles (*n*=73)

The breadth of studies identified reflect the complexity and diversity of ACF policies and their characteristics. There has been a gradual increase in the number of articles on ACF since the first identified (e.g. from 1979), and 67% (*n*=49/73) were published in the period 2010-2018 (*Table 1*). The studies were conducted in all WHO regions, i.e. the African region (22%, *n*=16/73), the Americas (North America) (14%, *n*=10/73), the Eastern Mediterranean region (1%, *n*=1/73), the European region (23%, *n*=17/73), South-East Asia (7%, *n*=5/73) and the Western Pacific region (12%, *n*=9/73). The most common types of articles were classified as quantitative papers (41%, *n*=30/73), reviews (22%, *n*=16/73) and qualitative studies (12%,

For peer review only - http://bmjopen.bmj.com/site/about/guidelines.xhtml

n=9/73). The most frequent ACF target groups were contacts (25%, *n*=18/73) and migrants from high-incidence countries (32%, *n*=23/73), while 14% percent (*n*=10/73) of the articles focused on screening of defined communities. The remaining studies focused on different target groups which are specified in *Table 1.*

Table 1 Characteristics of the articles included in the scoping review (n=73)

(Please see end of text file.)

Fig 1. PRISMA flow diagram

(Please see separate Word file entitled "Biermann_fig 1".)

Antecedents

Active case-finding has been implemented for many decades primarily in high-income countries, starting with mass screening campaigns in the general population in the 1950s and 1960s, then moving towards specific risk populations in recent decades, such as migrants from high-incidence countries and prison populations [15]. In low- and middle-income countries, the interest in ACF has increased in recent years, mainly as a response to a sustained case detection gap documented in annual Global TB Reports produced by WHO [3] and the emergence of new WHO guidelines [16].

Generally, TB programmes have moved from traditionally vertical approaches and exclusively facility-based case finding among people seeking care with TB symptoms (so-called "passive case-finding") towards being closer to the client through decentralized, community-based solutions and outreach activities, including ACF – similar to community-based testing services that have increased access to HIV testing and care [17,18].

For peer review only - http://bmjopen.bmj.com/site/about/guidelines.xhtml

In 1976, the WHO Expert Committee on TB recommended that countries abandon *indiscriminate* mobile mass radiography, as evidence showed the inefficiency of population-wide screening in settings that had seen TB rates drop dramatically since the second World War [19]. It should be noted that the Expert Committee emphasized that TB screening should still be done in selected risk groups [19,20].

The current WHO guidelines on systematic screening for active TB were released in 2013 [1]. These guidelines still discourage *indiscriminate* mass screening and strongly recommend ACF only in selected high-risk groups, while conditionally recommending screening in other high-risk groups. Conditional recommendations are those for which the benefits are expected to outweigh the risks, while the trade-offs, such as cost-effectiveness, feasibility or affordability may be uncertain. For example, WHO has made a conditional recommendation (very low-quality evidence) for screening certain clinical risk groups in settings where the TB prevalence is over 100 per 100,000 population (while not recommending such screening in countries with a prevalence of less than 100 per 100,000 population) [1].The 2013 WHO guidelines – reflected in the latest 2015 WHO's End TB Strategy [2] and aligned with the Sustainable Development Goals – put ACF back on the agenda. The guidelines provide principles for how to test and expand an approach that had been largely missing in previous global TB strategies, including the Directly Observed Treatment, Short Course (DOTS) Strategy from 1994 [87] and the Stop TB Strategy 2006-2015 (although contact investigation and screening people living with HIV was included in the latter) [88]. Moreover, the guidelines emphasize the importance of basing ACF policy on local epidemiology and to perform careful evaluations of ACF implementation, yield and impact to improve the ACF evidence base [20]. Today, ACF is considered a global health priority [21,22].

Components

For peer review only - http://bmjopen.bmj.com/site/about/guidelines.xhtml

According to WHO [1], the main components of ACF include 1) having high-quality TB diagnosis, treatment, care, management and support for patients, as well as the capacity to scale these up (if needed), before screening is initiated; 2) prioritizing risk groups; 3) choosing a suitable screening algorithm; 4) following established ethical principles; 5) optimizing synergies with the delivery of other health and social services; and 6) monitoring and re-assessing ACF approaches.

The components of ACF, as well as those of the wider health system that ACF depends on, are well-described in the literature. It is important for high-quality diagnosis, treatment, care, management, patient support and capacity for scale-up to be available [19]. Risk groups must be prioritized based on assessments of benefits and risks, feasibility, acceptability, the number needed to screen to detect a case of TB and the cost-effectiveness of ACF [19], while also considering the heterogeneity of the risk group [23].

The overall ACF approach must be flexible, patient-centred [15,24] and culturally sensitive [25]. In resource poor settings, ACF is often backed by multi-channel financing mechanisms, including from international donors [26]. Record-keeping, monitoring and evaluation are described as being essential [27,28]. Incentives are sometimes deemed necessary, e.g. for health workers [5,29], laboratory technicians [30], screening participants [11,31,32,33,34,35] and TB patients [29,35,36]. The articles also emphasized ACF-specific health education as paramount – for patients [31,37,38,39], their families [40], providers [24] and community health workers (CHW) [36,37]. The training of CHWs should be followed by supervision [41,42]. In addition, community involvement [32,39,42,43] and social mobilization [29] are major parts of ACF. One study described how the involvement of community leaders was a key component to improve trust among patients and contacts [31].

Lastly, the studies underlined how vital it is to collaborate with other disease programmes [33,34,43], agencies and sectors [26,27], and to integrate ACF within existing systems

For peer review only - http://bmjopen.bmj.com/site/about/guidelines.xhtml

[18,44]. The latter includes collaboration between providers and community-based organizations [43], and between HIV and TB services [45].

Influencing factors

In the following section, we summarize influencing factors for ACF policy development and implementation. We chose to speak of "influencing factors" rather than facilitators and barriers, as one and the same factor might be a facilitator in one context and a barrier in another, e.g. health workers' satisfaction might be a powerful facilitator for ACF [42], while the lack thereof could be a strong barrier as well [46].

Influencing factors for ACF policy development

Active case-finding policy development processes were not well described in the literature. Policy development comprises the investment of governments and donors in ACF. Only two articles reported facilitators and/or barriers for ACF policy development: politics [46,47] and laws [47]. Welshman [46] describes how, in the mid-1950s, the Ministry of Health in the United Kingdom subverted pressure from stakeholders who were in favour of compulsory ACF at ports of entry – the Ministry "argued that the problem [of TB in migrants] was a minor one" so a policy was not developed. On the other hand, laws such as the 1958 Commonwealth Migration Act were described, which made screening compulsory, and thus influenced ACF policy development in the United Kingdom [47].

Influencing factors for ACF policy implementation

Policy implementation processes in the area of ACF are widely researched. In total 43, articles reported barriers and/or facilitators for ACF implementation (*Table 2*). Most articles

For peer review only - http://bmjopen.bmj.com/site/about/guidelines.xhtml

mentioned factors at the level of the health system, as well as the individual and community level.

Table 2 Barriers and facilitators for active case-finding policy implementation (n=43)

(Please see end of text file.)

Health system level

It is fundamental to consider health system factors when putting ACF policy into practice and identifying which factors influence ACF policy implementation the most. We derived six major themes from the articles: 1) availability of financial resources, 2) existing systems and structures, 3) availability of diagnostic tests, 4) staff experience and motivation, 5) collaboration between different actors, and 6) implementation of a person-centred approach.

Availability of financial resources

Many articles (35%, *n*=15/43) mentioned financial resources as an influencing factor for ACF policy implementation. Finances were described as being scarce at different levels of the health system [40], for the provision of staff [39,42], drugs and equipment [48].

Existing systems and structures

Existing systems and structures were reported as influencing factors for ACF policy implementation by 26% (*n*=11/43) of the articles. Studies elaborated on the availability of health and social services [14,19] and laboratory networks [21] that could be used for ACF implementation.

Availability of diagnostic tests of sufficient sensitivity and specificity

Nineteen percent (*n*=8/43) of the articles elaborated on the availability of diagnostic tests and services as key influencing factor for ACF policy implementation – with many articles

For peer review only - http://bmjopen.bmj.com/site/about/guidelines.xhtml

referring to the availability of molecular diagnostic tests such as Xpert MTB/RIF® [27,36,49,50]. Decentralization of health care services, national coverage of diagnostics and task sharing were perceived as facilitators of ACF policy implementation, but unavailable in many settings [33].

Staff experience and motivation

Thirty-three percent (*n* =14/43) of the studies described staff experience and motivation as influencing factors for ACF policy implementation. One study elaborated on how, on the one hand, staff members would feel motivated by increased case detection and awareness-raising through ACF. On the other hand, they would be frustrated when being “shouted at, ignored, (and) harassed” by target communities, in this case people living in poor urban settlements [39]. The over-burdening of staff was highlighted by 16% (*n*=7/43) of the articles, e.g. leading to absenteeism [51], and thus inhibiting ACF implementation.

Collaboration between different actors

A variety of studies (16%, *n*=7/43) described collaboration as an important influencing factor for ACF policy implementation. One example is the collaboration between community workers, health centre staff and laboratory technicians in delivering diagnostic services as part of ACF [16]. Moreover, Akkerman et al. described the positive impact on diagnostic accuracy and the proportion of follow-up medical interventions when there is close collaboration with a limited number of experienced, high-volume chest radiograph readers when screening asylum seekers and other high-risk groups [52].

Implementation of a person-centred approach

The need for a person-centred approach to ACF policy implementation was referred to by 19% (*n*=8/43) of the articles, e.g. adopting approaches including health care providers being non-judgmental and supportive of patients and their families during diagnosis and follow-up, [53] and assuring confidentiality [22,52]. Another 12% (*n*=5/43) of the articles emphasized

For peer review only - http://bmjopen.bmj.com/site/about/guidelines.xhtml

health education for the general public, screening participants, as well as TB patients and their families as a necessity.

Individual and community

Active case-finding policies should be *for* individuals and communities and their influence on successful implementation is elaborately described in the literature. Three overarching themes emerged from the studies: 1) stigma and discrimination, 2) individual characteristics and socio-cultural factors, and 3) knowledge and awareness.

Stigma and discrimination

Many studies (37%, *n*=16/43) reported stigma and discrimination linked to TB as influencing factors for ACF policy implementation. The consequences could be the avoidant behaviour of a contact of an index TB case, such as providing incorrect phone numbers and/or wrong directions to their homes [14], or misconceptions about TB [24,43]. Issues of fear, either of TB disease [23,39,53], the process of ACF [54], or the wider implications of being identified as a TB case, e.g. through migrant screening programmes, where being diagnosed with TB case has in some instances resulted in deportation [34,38,55,56]. Studies also reported the lack of trust of patients and their families as key barrier for ACF policy implementation [41,51] – not only for early diagnosis, but for the sustained commitment to addressing social determinants of TB [57].

Individual characteristics and socio-cultural factors

Twenty-eight percent (*n*=12/43) of the articles mentioned the social characteristics of the persons screened as key influencing factors for ACF policy implementation, e.g. for ensuring equitable access to care [21,39,58] and promoting conducive health-seeking behaviour [23,41,43]. Moreover, the articles frequently reported socio-cultural factors and language (33%, *n*=14/43) as influencing factors for ACF policy implementation. Culture was

For peer review only - http://bmjopen.bmj.com/site/about/guidelines.xhtml

commonly described as a barrier to ACF policy implementation [34,41,59], e.g. if cultural beliefs would negatively influence the receptiveness to community health workers' advice. Only one study mentioned culture as a facilitator for putting ACF into practice, i.e. if culture meant respect toward professional authority [59].

Knowledge and awareness

Lastly, 19% (*n*=8/43) of the articles reported factors related to knowledge and awareness as influencing factors for ACF policy implementation. One such factor could be the understanding of the purpose of ACF [24]. Also, knowledge and awareness are closely linked to persisting stigma and discrimination, e.g. Ayakaka et al. [14] describe how health workers attributed TB-associated stigma to a lack of general knowledge in the community about TB.

Discussion

Characteristics of the included articles

We identified 73 articles addressing antecedents, components and/or influencing factors for ACF policy development and implementation. The included studies originated from all WHO regions. Africa and Asia are home to 90% of the 30 high TB burden countries (53% and 37% respectively) [89], which may explain why studies were more frequently conducted from these continents: 22% of the studies were conducted in Africa, 12% in the Western-Pacific region and 7% in South-East Asia. Studies conducted in the European region (23%) were predominantly about different forms of migrant screening. There was no study from Latin America among the 73 included articles, and there were no articles in the Spanish language among those excluded.

Most studies were published after 2010, which may be a result of programmes implementing WHO guidelines on systematic screening [1] as well as the growing number of prevalence

For peer review only

surveys in different settings [39]. We classified 41% of the articles as quantitative, e.g. cross-sectional and cohort studies, effectiveness and cost-effectiveness studies, randomized controlled trials and programme evaluations. The next most common study types were reviews (22%) and qualitative studies (12%). The first qualitative studies on our topic were published in 2003 [24,54]. We only identified two mixed-methods studies, one published in 2013 [42] and one in 2015 [39]. The mixed-methods design combined with quantitative ACF evaluation might become more commonly used to explore the complexity of ACF policy development and implementation in the future, as it has the potential to both increase contextual understanding and reduce biases.

Amongst the broad range of possible target groups for ACF, most of the articles focused either on TB contacts or migrants. The focus on the latter was mainly in low-incidence countries and may be a result of long-standing national health and migration policies supporting these types of screening. Yet, other studies covered vulnerable groups, such as homeless people, people living with HIV or people living in congregate settings, as well as the urban poor. Some articles included analysis of more than one high-risk group. There were no studies on indiscriminate mass screening. However, there were studies targeting broad communities, e.g. communities with high HIV prevalence.

Antecedents

Despite the growing number of studies (e.g. operational studies), there is still a relative lack of evidence about the health benefits for individuals, the epidemiological impact and cost-effectiveness of different ACF approaches. Nonetheless, the interest in ACF has clearly increased in recent years. The persistent case detection gap, the 2013 WHO guidelines on systematic screening [1], and ACF being mentioned as a core component of the End TB Strategy [2] seem to have motivated countries to include ACF in national TB strategies.

For peer review only - http://bmjopen.bmj.com/site/about/guidelines.xhtml

444 Universal Health Coverage and the Sustainable Development Goals have only marginally been mentioned as important for stepping up the TB response [33]. However, the Sustainable Development Goals – which aim to “leave no one behind” – might have contributed to a greater interest in ACF to leave no undiagnosed TB patients behind.

448 Not having enough evidence to drive ACF policy development and implementation means that decisions to embark on ACF rely largely on stakeholders’ tacit knowledge, experience, values and preferences. Demonstrated screening yield in the same or other settings could also motivate further ACF implementation, although the number of cases detected may not be a relevant public health impact measure in itself. The above-mentioned WHO guidelines use the term “indirect evidence” [1]. Strong “direct evidence” exists that early diagnosis and correct TB treatment reduce morbidity, mortality and transmission. This can constitute, for example, “indirect evidence” for ACF, based on the logic that if ACF leads to early detection and treatment then it should also lead to better health outcomes and less transmission, despite the lack of trial data demonstrating a direct health impact of ACF. Similar reasoning can be used to describe potential harms associated with ACF, e.g. based on modelling of the risk of false positive TB diagnoses in different epidemiological contexts. “Indirect evidence”, tacit knowledge, experience, values and preferences are likely to be important antecedents for national policy.

Components

The components of ACF policy, and of the system which ACF is part of, are complex. The articles reported on the prerequisites of the given system, its resources [46], capacity [42,30] and tools [90]. However, they also elaborated on the need for integrating ACF into health systems [41,60], as well as educating and engaging health care providers [5,21], communities [12,29,39,44], screening participants [35] and TB patients and their families [25,34,35] for

For peer review only - http://bmjopen.bmj.com/site/about/guidelines.xhtml

ACF implementation to succeed. All components seem indispensable for successful ACF policy development and implementation. Yet, in real-life settings, rarely all components are in place.

Which ACF components are essential and which are non-essential? The 2013 WHO guidelines on systematic screening list six key principles for screening for active TB, the first principle being: “Before screening is initiated, high-quality TB diagnosis, treatment, care, management and support for patients should be in place, and there should be the capacity to scale these up further to match the anticipated rise in case detection that may occur as a result of screening“ [1]. Yet, countries might not even completely adhere to this very first principle, e.g. due to a lack of resources, before a country embarks on ACF policy development and implementation.

Influencing factors

Scarce research on ACF policy development

Our findings indicate a paucity of research focusing specifically on ACF policy development. The only two factors described – politics and laws – were mentioned by the same author in two different articles and concerned migrant TB screening in a low-incidence country [46,47]. Even though not documented as such in the literature, data on the epidemiological situation of a country and local evidence regarding impact are assumed to be key factors influencing ACF policy development [1]. These types of data and evidence are needed to identify high-risk groups [90] and devise appropriate screening algorithms. An assessment of the epidemiological situation might be based on data from regularly collected datasets or prevalence surveys, and WHO has developed a tool to help countries estimate screening yield and costs based on such data to identify appropriate risk groups and approaches [91].

For peer review only - http://bmjopen.bmj.com/site/about/guidelines.xhtml

Interestingly, the evolution of the funding landscape for ACF, e.g. through TB REACH, has not been explicitly mentioned as an influencing factor for ACF policy development or implementation, though it has been a likely game-changer. TB REACH is a case-finding initiative funded largely by Global Affairs Canada and coordinated by the Stop TB Partnership [92]. The initiative has not only funded ACF activities but raised awareness about the importance of ACF. It has contributed to the local evidence base, while the uptake of the evidence generated by TB REACH may be considered but not mentioned in the studies included in this review. Four articles mentioned TB REACH as funder of their research, with the disclaimer that the initiative had no role in study design, data collection and analysis [16,42,60,61]. Other publications acknowledged its role in implementing ACF when describing the study methods, e.g. that TB REACH funded door-to-door screening [39] or the implementation of different ACF models [62] and that the initiative upgraded an ACF strategy by introducing new diagnostics which still have limited availability in many settings (e.g. Xpert MTB/RIF®) [36]. It is important to reflect on and document the influence that TB REACH, other international funding mechanisms and technical agencies have on ACF policy development and implementation.

ACF policy and social determinants of health

Many barriers and facilitators for implementing ACF policies lie at the level of communities and individuals. These factors include social determinants such as the educational level [14,35], employment status [27,63] and access to care [64] of the target population. These factors can limit an individual's or a community's participation in ACF. ACF policies cannot be put in practice successfully if implementation barriers prevail. However, rather than talking about "missing cases" and communities that are "hard to reach", it may be useful to consider if health services are missing or hard to reach instead.

For peer review only - http://bmjopen.bmj.com/site/about/guidelines.xhtml

Individuals or communities may be ACF target groups precisely because health services are missing or hard to reach. As such, ACF could be a way to by-pass access barriers, e.g. by using mobile units. Yet, even when designing services to be more accessible, target populations may still not come forward, take the next step of referral to complete the diagnostic pathway or adhere to treatment due to socioeconomic barriers.

We argue that active case-finding polices thus must be designed and implemented with careful consideration of social determinants in a given context to avoid possible harm. Whitehead et al. [93] described how interventions designed to help vulnerable populations may be "implemented in such a way as to stigmatize the very people the programme was designed to help and, in so doing, push them to avoid the help on offer."

A comprehensive view on ACF policies

A comprehensive approach in developing and implementing ACF policies is required that includes health education as an important component to ACF policies, and that ensures collaboration between key stakeholders, not only between patients and providers, but between providers and local health authorities, between agencies, and across sectors and disease programmes.

Although this review focused on the factors that influence ACF policy and implementation, it is important to also reflect on how ACF policy influences its context in return, e.g. not only factors at the level of the community and the individual, but at the level of the health system and beyond; How may the ACF policy increase or decrease stigma in a community? How may ACF policy influence the human and financial resources available in a health system? Only then may we see the real impact of ACF and be able to make equitable decisions in policy development and implementation processes.

For peer review only - http://bmjopen.bmj.com/site/about/guidelines.xhtml

For peer review only

Future research

This scoping review demonstrated that while we know much about facilitators and barriers for ACF policy implementation, we know less about how to strengthen those facilitators and how to overcome those barriers. A major knowledge gap remains when it comes to understanding which contextual factors influence ACF policy development.

Limitations

Our scoping review has some limitations. To increase the feasibility, we limited the search to MEDLINE, Web of Science, The Cochrane Database of Systematic Reviews and the WHO Library. We did not do a critical appraisal of the individual sources of evidence or within sources of evidence, nor did we describe sources of funding for the included sources of evidence. Such appraisals and descriptions would have allowed us to not only discover the scarcity of research, but to characterize the quality of and gaps in the evidence base and enable the development of robust recommendations. We leave these questions for future systematic reviews to address.

We acknowledge that we may have been able to provide a more in-depth overview of antecedents, components and influencing factors for ACF policy development and implementation by including additional databases, searching grey literature and references of included studies, and contacting authors for more information, which could be included in a future systematic review. However, we believe that our review still adds value to the current body of knowledge on ACF, by providing collated and comprehensive insights into the peer-reviewed scientific literature. We also limited the inclusion to studies written in English, which may have resulted in the exclusion of eligible studies in other languages, e.g. Spanish. Furthermore, the results from this scoping review are only up to date as of 31 January 2018. The data were extracted by one reviewer only. However, the data are likely valid, as a pilot-

For peer review only

For peer review only - http://bmjopen.bmj.com/site/about/guidelines.xhtml

test was conducted prior to embarking on data extraction with members of the study team. A second reviewer verified a random selection of the data.

Another limitation was that the included articles often did not distinguish between the implementation of an ACF policy versus the implementation of an ACF programme or intervention. This is likely due to inconsistent use of the terms in the literature. As such, our results are likely applicable to both. Furthermore, the reporting of ACF policy development and implementation varied in their completeness across the included articles, and as such, our data are limited by the details described in the literature, e.g. most papers described steps to implementation, but did not provide details on non-response or unsuccessful practices. There is a risk of publication bias.

Conclusions

We identified some main themes regarding the antecedents, components and influencing factors for ACF policy development and implementation. However, evidence remains scarce especially concerning policy development. Research on ACF is required to understand, inform and improve policy development and implementation.

List of abbreviations

ACF – Active Case-Finding

ART – Antiretroviral Therapy

CHW – Community Health Worker

DOTS – Directly Observed Treatment, Short Course

HIV – Human Immunodeficiency Virus

LTBI – Latent Tuberculosis Infection

PCF – Passive Case-Finding

For peer review only - http://bmjopen.bmj.com/site/about/guidelines.xhtml

PRISMA - Preferred Reporting Items for Systematic reviews and Meta-Analyses

PRISMA-ScR – Preferred Reporting Items for Systematic reviews and Meta-Analyses extension for Scoping Reviews

TB – Tuberculosis

TiAb – Title and abstract

WHO – World Health Organization

Ethics approval and consent to participate

N/A

Consent for publication

N/A

Availability of data and material

The materials supporting the conclusions of this article are included within the article and its additional files. No additional data are available.

Additional files

Additional file 1: PRISMA Extension for Scoping Reviews (PRISMA-ScR) checklist.

Additional file 2: Literature search strategy used for Medline; additional search strategies available from the authors.

Additional file 3: Abridged data charting table.

Competing interests

We have no competing interests to declare.

Funding

This work was supported by the EU-Horizon 2020-funded IMPACT-TB project (grant 733174).

Author contributions

OB, KL, MC and KV conceived the study. OB developed the search strategy together with a medical librarian from Karolinska Institutet. OB and KV screened titles and abstracts, and then full-text articles. OB extracted, charted, analysed and interpreted the data. KV verified the data extraction by charting data from a random sample of studies for comparison. OB wrote the first draft of the manuscript. KL, MC and KV were consulted at various stages of the scoping review to provide input on the search strategy, data extraction, charting, analysis and the interpretation of the results, and provided comments on the manuscript. All authors read and approved the final manuscript.

For peer review only - http://bmjopen.bmj.com/site/about/guidelines.xhtml

Acknowledgements

OB is funded by the EU-Horizon 2020-funded IMPACT-TB project (grant 733174). The authors thank Carl Gornitzki, medical librarian at Karolinska Institutet, for supporting the development of the search strategy, and Gabriella Ekman, writing instructor at Karolinska Institutet's University Library for her valuable feedback in writing this manuscript.

References

1. World Health Organization. Systematic screening for active TB: principles and recommendations. Switzerland, Geneva: World Health Organization; 2013.
2. World Health Organization. Implementing the End TB Strategy: the essentials. Switzerland, Geneva: World Health Organization; 2015.
3. World Health Organization. Global TB Report 2019. Switzerland, Geneva: World Health Organization; 2019.
4. Storla DG, Yimer S, Bjune GA. A systematic review of delay in the diagnosis and treatment of TB. BMC Public Health. 2008;8:15. doi: 10.1186/1471-2458-8-15.
5. Blok L, Sahu S, Creswell J, Alba S, Stevens R, Bakker MI. Comparative meta-analysis of TB contact investigation interventions in eleven high burden countries. PLoS One. 2015;10:3:e0119822. doi: 10.1371/journal.pone.0119822.
6. Creswell J, Codlin AJ, Andre E, Micek MA, Bedru A, Carter EJ, et al. Results from early programmatic implementation of Xpert MTB/RIF testing in nine countries. BMC Infect Dis. 2014;14:2. doi: 10.1186/1471-2334-14-2.

For peer review only - http://bmjopen.bmj.com/site/about/guidelines.xhtml

7. Creswell J, Sahu S, Blok L, Bakker MI, Stevens R, Ditiu L. A multi-site evaluation of innovative approaches to increase TB case notification: summary results. PLoS One. 2014;9:4:9:e94465. doi: 10.1371/journal.pone.0094465.
8. Kranzer K, Afnan-Holmes H, Tomlin K, Golub JE, Shapiro AE, Schaap A, et al. The benefits to communities and individuals of screening for active TB disease: a systematic review. Int J Tuberc Lung Dis. 2013;17:432–46. doi: 10.5588/ijtld.12.0743.
9. Global Fund, World Health Organization and Stop TB Partnership Strategic Initiative. https://www.who.int/tb/joint-initiative/en/ (2019). Accessed 8 Apr 2019.
10. TB REACH. http://www.stoptb.org/global/awards/tbreach/ (2019). Accessed 8 Apr 2019.
11. National TB Control Program. National Strategic Plan for TB control for the period 2015-2020. Vietnam, Hanoi: Ministry of Health; 2014.
12. Joanna Briggs Institute. Methodology for JBI scoping reviews. Adelaide: Joanna Briggs Institute; 2015.
13. Tricco A, Lillie E, Zarin W, O'Brien KK, Colquhoun H, Levac D, et al. PRISMA Extension for Scoping Reviews (PRISMA-ScR): Checklist and Explanation. Ann Intern Med. 2018;169:467-473. doi: 10.7326/M18-0850.
14. Ayakaka I, Ackerman S, Ggita JM, Kajubi P Dowdy D, Haberer EF, et al. Identifying barriers to and facilitators of tuberculosis contact investigation in Kampala, Uganda: a behavioral approach. Implementation Sci. 2017;12:33. doi: 10.1186/s13012-017-0561-4.
15. Eang MT, Satha P, Yadav RP, Morishita F, Nishikiori N, van-Maaren P, Lambregts-van Weezenbeek C. Early detection of tuberculosis through community-based active case finding in Cambodia. BMC Public Health. 2012;12:469. doi: 10.1186/1471-2458-12-469.

For peer review only - http://bmjopen.bmj.com/site/about/guidelines.xhtml

16. Lorent N, Choun K, Thai S, Kim T, Huy S, Pe R, et al. Community-Based Active Tuberculosis Case Finding in Poor Urban Settlements of Phnom Penh, Cambodia: A Feasible and Effective Strategy. PLoS One. 2014;9:3:e92754. doi: 10.1371/journal.pone.0092754.

17. Godfrey-Fausset P, Ayles H. Can we control tuberculosis in high HIV prevalence settings? Tuberculosis. 2003;83:68-76. doi: 10.1016/S1472-9792(02)00083-5.

18. Corbett EL, MacPherson. Tuberculosis screening in high human immunodeficiency virus prevalence settings: turning promise into reality. Int J Tuberc Lung Dis. 2013;17:9:1125-1138. doi: 10.5588/ijtld.13.0117.

19. Lönnroth K, Corbett E, Golub J, Godfrey-Faussett P, Uplekar M, Weil D, Raviglione M. Systematic screening for active tuberculosis: rationale, definitions and key considerations. Int J Tuberc Lung Dis. 2013;17:3:289-298. doi: 10.5588/ijtld.12.0797 STATE.

20. Zenner D, Hafezi H, Potter J, Capone S, Matteelli A. Effectiveness and cost-effectiveness of screening migrants for active tuberculosis and latent tuberculosis infection. Int J Tuberc Lung Dis. 2017;21:9:965-976. doi: 10.5588/ijtld.16.0935.

21. Golub JE, Mohan CI, Comstock GW, Chaisson RE. Active case finding of tuberculosis: historical perspective and future prospects. Int J Tuberc Lung Dis. 2005;9:11:1183-1203. [PMID: 16333924]

22. Kerrigan D, West N, Tudor C, Hanrahan C, Lebina L, Msandiwa, et al. Improving active case finding for tuberculosis in South Africa: informing innovative implementation approaches in the context of the Kharitode trial through formative research. Health Res Policy Sys. 2017;15:42. doi: 10.1186/s12961-017-0206-8.

For peer review only

For peer review only - http://bmjopen.bmj.com/site/about/guidelines.xhtml

23. Swigat V, Kolb R. Homeless persons' decisions to accept or reject public health disease-detection services. Public Health Nurs. 2004;21:2:162-170. doi: 10.1111/j.0737-1209.2004.021210.x.

24. Shrestha-Kuwahara R, Wilce M, DeLuca N, Taylor Z. Factors associated with identifying tuberculosis contacts. Int J Tuberc Lung Dis. 2003;7:12:S510-516

25. de Vries SG, Cremers AL, Heuvelings CC, Greve PF, Visser BJ, Bélard S, et al. Barriers and facilitators to the update of tuberculosis diagnostic and treatment services by hard-to-reach populations in countries of low and medium tuberculosis incidence: a systematic review of qualitative literature. Lancet Infect Dis. 2017;17:e128-43. doi: 10.1016/S1473-3099(16)30531-X.

26. Li J, Liu X-Q, J S-W, Li X, Yu F, Wang Y, et al. Improving tuberculosis case detection in underdeveloped multi-ethnic regions with high disease burden: a case study of integrated control program in China. Infect Dis Poverty. 2017;6:151. doi: 10.1186/s40249-017-0365-4

27. Baxter S, Goyder E, Chambers D, Johnson M, Preston L, Booth A. Interventions to improve contact tracing for tuberculosis in specific groups and in wider populations: an evidence synthesis. Health Services and Delivery Research. 2017;5:1. doi: 10.3310/hsdr05010.

28. Van den Bosch CA, Roberts JA. Tuberculosis screening of new entrants; how can it be made more effective? Journal Public Health Med. 2000;22:2:220-223.

29. Hinderaker SG, Rusen ID, Chiang C-Y, Yan L, Heldal E, Enarson DA. The FIDELIS initiative: innovative strategies for increased case finding. Int J Tuberc Lung Dis. 2011;15:1:71-76.

For peer review only

For peer review only - http://bmjopen.bmj.com/site/about/guidelines.xhtml

30. Erkens C, Slump E, Kamphorst M, Keizer S, van Gerven PJHJ, Bwire R, Berkel M, et al. Coverage and yield of entry and follow-up screening for tuberculosis among new immigrants. Eur Respir J. 2008;32:153-161. doi: 10.1183/09031936.00137907.

31. Tupasi TE, Radhakrishna S, Co VM, Villa MLA, Quelapio MID, Mangubat NV, et al. Bacillary disease and health seeking behavior among Filipinos with symptoms of tuberculosis: implications for control. Int J Tuberc Lung Dis. 2000;4:12:1126-1132.

32. Cook VJ, Shah L, Gardy J. Modern contact investigation methods for enhancing tuberculosis control in Aboriginal communities. Int J Circumpolar Health. 2012;71:18643. doi: 10.3402/ijch.v71i0.18643.

33. Suthar AB, Zachariah R, Harries AD. Ending tuberculosis by 2030: can we do it? Int J Tuberc Lung Dis. 2016;20:9:1148-1154. doi: 10.5588/ijtld.16.0142.

34. Tankimovich M. Barriers to and interventions for improved tuberculosis detection and treatment among homeless and immigrant populations: a literature review. J Community Health Nurs. 2013;30:83-95. doi: 10.1080/07370016.2013.778723.

35. Ayles H, Muyoyeta M, Du Toit E, Schaap A, Floyd S, Simwinga M, Shanaube K, et al. Effect of household and community interventions on the burden of tuberculosis in southern Africa: the ZAMSTAR community-randomised trial. Lancet. 2013;382:1183-94. doi: 10.1016/ S0140-6736(13)61131-9.

36. Morishita F, Yadav R-P, Eang MT, Saint S, Nishikiori N. Mitigating Financial Burden of Tuberculosis through Active Case Finding Targeting Household and Neighbourhood Contacts in Cambodia. PLoS One. 2016;11:9:e0162796. doi: 10.1371/ journal.pone.0162796.

37. Fox GJ, Loan LP, Nhung NV, Loi NT, Sy DN, Britton WJ, Marks GB. Barriers to adherence with tuberculosis contact investigation in six provinces of Vietnam: a

For peer review only

752 nested case-control study. BMC Infect Dis. 2015;15:103. doi: 10.1186/s12879-015-
753 0816-0.
754 38. Kulane A, Ahlberg BM, Berggren I. "It is more than the issue of taking tablets": The
755 interplay between migration policies and TB control in Sweden. Health Policy.
756 2010;97:26-31. doi: 10.1016/j.healthpol.2010.02.014.
757 39. Lorent N, Choun K, Malhotra S, Koeut P, Thai S, Khun KE, et al. Challenges from
758 Tuberculosis Diagnosis to Care in Community-Based Active Case Finding among the
759 Urban Poor in Cambodia: A Mixed-Methods Study. PLoS One. 2015;10:7:e0130179.
760 doi: 10.1371/journal.pone.0130179.
761 40. Karki B, Kittel G, Bolokon I, Duke T. Active community-based case finding for
762 tuberculosis with limited resources: estimating prevalence in a remote area of Papua
763 New Guinea. Asia Pac J Public Health. 2017;28:1:17-27. doi:
764 10.1177/1010539516683497.
765 41. Getnet F, Hashi A, Mohamud S, Mowlid H, Klinkenberg E. Low contribution of
766 health extension workers in identification of persons with presumptive pulmonary
767 tuberculosis in Ethiopian Somali Region pastoralists. BMC Health Serv Res.
768 2017;17:193. doi: 10.1186/s12913-017-2133-3.
769 42. Yassin MA, Datiko DG, Tulloch O, Markos P, Aschalew M, Shargie EB, et al.
770 Innovative community-based approached doubled tuberculosis case notification and
771 improve treatment outcome in Southern Ethiopia. PLoS One. 2013;8:5:e63174. doi:
772 doi:10.1371/journal.pone.0063174.
773 43. Seedat F, Hargreaves S, Friedland JS. Engaging New Migrants in Infectious Disease
774 Screening: A Qualitative Semi-Structured Interview Study of UK Migrant Community
775 Health-Care Leads. PLoS One. 2014;9:10:e108261. doi:
776 10.1371/journal.pone.0108261.

For peer review only

44. Sekandi JN, Dobbin K, Oloya J, Okwera A, Whalen CC, Corso PS. Cost-Effectiveness Analysis of Community Active Case Finding and Household Contact Investigation for Tuberculosis Case Detection in Urban Africa. PLoS One. 2015;10:2:e0117009. Doi: 10.1371/journal.pone.0117009.

45. Kranzer K, Lawn SD, Meyer-Rath G, Vassall A, Raditlhalo E, Govindasamy D, et al. Feasibility, Yield, and Cost of Active Tuberculosis Case Finding Linked to a Mobile HIV Service in Cape Town, South Africa: A Cross-sectional Study. PLoS Med. 2012;9:8:e1001281. doi: 10.1371/journal.pmed.1001281.

46. Welshman J. Compulsion, localism, and pragmatism: the micro-politics of tuberculosis screening in the United Kingdom, 1950-1965. Soc Hist Med. 2006;19:2:295-312. doi: 10.1093/shm/hkl002.

47. Welshman J, Bashford A. Tuberculosis, migration, and medical examination: lessons from history. J Epidemiol Community Health. 2006;60:282-284. doi: 10.1136/jech.2005.038604.

48. Mor Z, Lerman Y, Leventhal A. Pre-immigration screening process and pulmonary tuberculosis among Ethiopian migrants in Israel. Eur Respir J. 2008;32:413-418. doi: 10.1183/09031936.00145907.

49. Abubakar I, Stagg H, Cohen T, Mangtani P, Rodrigues LC, Pimpin L, Watson JM, Squire SB, Zumla A. Controversies and unresolved issues in tuberculosis prevention and control: a low-burden-country perspective. J Infect Dis. 2012;205:S293-300. doi: 10.1093/infdis/jir886.

50. Dierberg KL, Dorjee K, Salvo F, Cronin WA, Boddy J, Cirillo D, Sadutshang T, et al. Improved detection of tuberculosis and multidrug-resistant tuberculosis among Tibetan refugees, India. Emerg Infect Dis. 2016;22:3. doi: 10.3201/eid2203.140732.

For peer review only

For peer review only - http://bmjopen.bmj.com/site/about/guidelines.xhtml

51. Harper I, Fryatt R, White A. Tuberculosis case finding in remote mountainous areas – are microscopy camps of any value? Experience from Nepal. Tuber Lung Dis. 1996;77:384-388.

52. Akkerman OW, de Lange WCM, Schölvinck EH, Wolters B, Aartsma Y, van der Werf TS, van Hest R. Implementing tuberculosis entry screening for asylum seekers: the Groningen experience. Eur Respir J. 2016;48:261-264. doi: 10.1183/13993003.00112-2016.

53. Southern A, Premaratne N, English M, Balazs J, O'Sullivan D. Tuberculosis among homeless people in London: an effective model of screening and treatment. Int J Tuber Lung Dis. 1999;3:11:1001-1008.

54. Chemtob D, Leventhal A, Weiler-Ravell D. Screening and management of tuberculosis in immigrants: the challenge beyond professional competence. Int J Tuberc Lung Dis. 2003;7:10:959-966.

55. Brewin P, Jones A, Kelly M, McDonald M, Beasley E, Sturdy P, et al. Is screening for tuberculosis acceptable to immigrants? A qualitative study. J Public Health. 2006;28:3:253-260. doi: 10.1093/pubmed/fdl031.

56. MacPherson DW, Gushulak BD. Balancing prevention and screening among international migrants with tuberculosis: Population mobility as the major epidemiological influence in low-incidence nations. Public Health. 2006;120:712-723. doi: 10.1016/j.puhe.2006.05.002.

57. Lönnroth K, Migliori GB, Abubakar I, D'Ambrosio L, de Vries G, Diel R, Douglas P, et al. Towards tuberculosis elimination: an action framework for low-incidence countries. Eur Respir J. 2015;45:928-952. doi: 10.1183/09031936.00214014.

58. Mayo K, White S, Oates SK, Franklin F. Community collaboration: prevention and control of tuberculosis in a homeless shelter. Public Health Nurs. 1997;13:2:120-127.

For peer review only

For peer review only - http://bmjopen.bmj.com/site/about/guidelines.xhtml

59. Tardin A, Dominicé Dao M, Ninet B, Janssens J-P. Tuberculosis cluster in an immigrant community: case identification issues and a transcultural perspective. Trop Med Int Health. 2009;14:9:995-1002. doi: 10.1111/j.1365-3156.2009.02325.x.

60. Datiko DG, Yassin MA, Theobald SJ, Blok L, Suvanand S, Creswell J, Cuevas LE. Health extension workers improve tuberculosis case finding and treatment outcome in Ethiopia: a large-scale implementation study. BMJ Glob Health. 2017;2:e000390. doi: 10.1136/ bmjgh-2017-000390.

61. Khan K, Hirji MM, Miniota J, Hu W, Wang J, Gardam M, Rawal S, et al. Domestic impact of tuberculosis screening among new immigrants to Ontario, Canada. CMAJ. 2015;187:16:e473-E481.2015. doi: 10.1503/cmaj.150317.

62. Adejumo AO, Azuogu B, Okorie O, Lawal OM, Onazi OJ, Gidado M, Daniel M, Okeibunor JC, Klinkenberg E, Mitchell EMH. Community referral for presumptive TB in Nigeria: a comparison of four models of active case finding. BMC Public Health. 2016;16:177. doi: 10.1186/s12889-016-2769-7.

63. Azman AS, Golub JE, Dowdy DW. How much is tuberculosis screening worth? Estimating the value of active case finding for tuberculosis in South Africa, China, and India. BMC Med. 2014;12:216. doi: 10.1186/s12916-014-0216-0.

64. Bell TR, Molinari NAM, Blumensaadt S, Selent MU, Arbisi M, Shah N, Christiansen D, et al. Impact of port of entry referrals on initiation of follow-up evaluations for immigrants with suspected tuberculosis: Illinois. J Immigr Minor Health. 2013;15:673-679. doi: 10.1007/s10903-013-9779-7.

65. O'Hara NN, Roy L, O'Hara LM, Spiegel JM, Lynd LD, FitzGerald JM, et al. Healthcare Worker Preferences for Active Tuberculosis Case Finding Programs in South Africa: A Best-Worst Scaling Choice Experiment. PLoS One. 2015;10:7: e0133304. doi: 10.1371/journal.pone.0133304.

For peer review only

For peer review only - http://bmjopen.bmj.com/site/about/guidelines.xhtml

66. Wright CW. An opinion survey of tuberculosis case-finding procedures. S Afr Med J. 1979;56:6:224-8.

67. Layton MC, Cantwell MF, Dorsinville GJ, Valway SE, Onorato IM, Frieden TR. Tuberculosis screening among homeless persons with AIDS living in single-room-occupancy hotels. Am J Public Health. 1995;85:11:1555-1559.

68. Hogan H, Coker R, Gordon A, Meltzer M, Pickles H. Screening of new entrants for tuberculosis: responses to port notifications. J Public Health. 2005;27:2:192-195. doi: 10.1093/pubmed/fdi019.

69. Klinkenberg E, Manissero D, Semenza JC, Verver S. Migrant tuberculosis screening in the EU/EEA: yield, coverage and limitations. Eur Respir J. 2009;34:1180-1189. doi: 10.1183/09031936.00038009.

70. Tan L, Altman RD, Nielsen NH. Screening nonimmigrant visitors to the United States for Tuberculosis. Arch Intern Med. 2001;161:334-340. doi: 10.1080/07370016.2013.778723.

71. Alvarez GG, Gushulak B, Rumman KB, Altpeter E, Chemtob D, Douglas P, et al. A comparative examination of tuberculosis immigrant medical screening programs from selected countries with high immigration and low tuberculosis incidence rates. BMC Infect Dis. 2011;11:3. doi: 10.1186/1471-2334-11-3.

72. Bloss E, Newbill K, Peto H, Rice MJ, Ainsowrth G, Travnicek R, Holcombe M, et al. Challenges and opportunities in a tuberculosis outbreak investigation in Southern Mississippi, 2005-2007. South Med J. 2011;104:11. doi: 10.1097/SMJ.0b0.3e318232679e.

73. Corbett EL, Bandason T, Duong T, Dauya E, Makamure B, Churchyard GJ, Williams BG, et al. Comparison of two active case-finding strategies for community-based diagnosis of symptomatic smear-positive tuberculosis and control of infectious

For peer review only - http://bmjopen.bmj.com/site/about/guidelines.xhtml

876 tuberculosis in Harare, Zimbabwe (DETECTB): a cluster-randomised trial. Lancet.
877 2010;376:1244-53. doi: 10.1016/S0140- 6736(10)61425-0.
878 74. Mhimbira FA, Cuevas LE, Dacombe R,Mkopi A, Sinclair D. Interventions to increase
879 tuberculosis case detection at primary healthcare or community-level services.
880 Cochrane Database Syst Rev. 2017;11:CD011432. doi:
881 10.1002/14651858.CD011432.pub2.
882 75. Fox GJ, Nhung NV, Sy DN, Hoa NLP, Anh LTN, Anh NT, Hoa NB, et al. Household-
883 contact investigation for detection of tuberculosis in Vietnam. N Engl J Med.
884 2018;378:221-9. doi: 10.1056/NEJMoa1700209.
885 76. Fox GJ, Barry SE, Britton WJ, Marks GB. Contact investigation for tuberculosis: a
886 systematic review and meta-analysis. Eur Respir J. 2013;41:140-156. doi:
887 10.1183/09031936.00070812.
888 77. Honarvar B, Odoomi N, Rezaei A, Haghighi HB, Karimi M, Hosseini A, Mazarei S, et
889 al. Pulmonary tuberculosis in migratory nomadic populations: the missing link in
890 Iran’s National Tuberculosis Programme. Int J Tuberc Lung Dis. 2014;18:3:272-276.
891 doi: 10.5588/ijtld.13.0650.
892 78. Kranzer K, Houben RMG, Glynn JR, Bekker L-G, Wood R, Lawn SD. Yield of HIV-
893 associated tuberculosis during intensified case finding in resource-limited settings: a
894 systematic review and meta-analysis. Lancet Infect Dis. 2010;10:93-102. doi:
895 10.1016/S1473-3099(09)70326-3.
896 79. Lönnroth K, Mor Z, Erkens C, Bruchfeld J, Nathavitharana RR, van der Werf MJ,
897 Lange C. Tuberculosis in migrants in low-incidence countries: epidemiology and
898 intervention entry points. Int J Tuberc Lung Dis. 2017;21:6:624-636. doi:
899 10.5588/ijtld.16.0845.

For peer review only - http://bmjopen.bmj.com/site/about/guidelines.xhtml

80. Mallick G, Shewade HD, Agrawal TK, Kumar AMV, Chadha SS. Enhanced tuberculosis case finding through advocacy and sensitisation meetings in prisons of Central India. Public Health Action. 2017;7:1:67-70. doi: 10.5588/pha.16.0109.

81. Mulder C, Erkens CGM, Kouw PM, Huisman EM, Meijer-Veldman W, Borgdorff MW, van Leth F. Missed opportunities in tuberculosis control in The Netherlands due to prioritization of contact investigations. Eur J Public Health. 2011;22:2:177-182. doi: 10.1093/eurpub/ckr017.

82. Mwansa-Kambafwile J, McCarthy K, Gharbaharan V, Venter FWD, Maitshotlo B, Black A. Tuberculosis Case Finding: Evaluation of a Paper Slip Method to Trace Contacts. PLoS One. 2013;89:e75757. doi: 10.1371/journal.pone.0075757.

83. Ospina JE, Orcau A, Millet J-P, Sánchez F, Casals M, Caylà JA. Community health workers improve contact tracing among immigrants with tuberculosis in Barcelonaa. BMC Public Health. 2012;12:158. doi: 10.1186/1471-2458-12-158.

84. Smit GSA, Apers L, Arrazola de Onate W, Beutels P, Dorny P, Forier A-M, et al. Cost-effectiveness of screening for active cases of tuberculosis in Flanders, Belgium. Bull World Health Organ. 2017;95:1:27-35. doi: 10.2471/BLT.16.169383.

85. Szkwarko D, Mercer T, Kimani S, Braitstein P, Buziba N, Carter EJ. Implementing intensified tuberculosis case-finding among street-connected youth and young adults in Kenya. Public Health Action. 2016;6:2. doi: 10.1371/journal.pone.0182185.

86. Shrivastava SR, Shrivastava PS. Tuberculosis: Active Case Finding Survey in an Urban area of India, in 2012s. J Res Health Sci. 2013;13:1:19-23.

87. World Health Organization. What is DOTS? A Guide to Understanding the WHO-recommended TB Control Strategy Known as DOTS. Switzerland, Geneva: World Health Organization; 1999.

For peer review only - http://bmjopen.bmj.com/site/about/guidelines.xhtml

88. World Health Organization. Building on and enhancing DOTS to meet the TB-related Millennium Development Goals. Switzerland, Geneva: World Health Organization; 2006.

89. World Health Organization. Use of high burden country lists for TB by WHO in the post-2015 era. Switzerland, Geneva: World Health Organization; 2015.

90. World Health Organization. Towards TB elimination: an action framework for low-incidence countries. WHO/ HTM/TB/2014.13. Switzerland, Geneva: World Health Organization; 2014.

91. Miller CR, Mitchell EMH, Nishikiori N, Zwerling A, Lönnroth K. A web-based tool for prioritizing risk groups and selecting algorithms for screening for active tuberculosis. (Submitted)

92. Stop TB Partnership. http://www.stoptb.org/ (2019). Accessed 25 Mar 2019.

93. Whitehead M, Dahlgren G. Concepts and principles for tackling social inequities in health: Levelling up Part 1. World Health Organization Collaborating Centre for Policy Research on Social Determinants of Health, University of Liverpool. Copenhagen: World Health Organization Regional Office for Europe; 2006.

For peer review only

Tables

Table 1 Characteristics of the articles included in the scoping review (n=73)

**Study characteristics**	**References**	**Number (%)**
**Year of publication**		
1979	66	1 (1%)
1995-1999	51,53,58,67	4 (5%)

2000-2009	17,21,23,24,28,30,31,46,47,48,54,56,59,68,69,70	16 (22%)
2010-2018	14,15,16,18,19,20,22,25,26,27,29,32,33,35,36,37,38, 39,40,41,42,44,45,49,50,52,55,57,60,61,62,63,64,65, 71,72,73,74,75,76,77,78,79,80,81,82,83,85,85	49 (67%)
**Region**		
Africa	14,20,22,35,41,42,44,45,60,62,63,65,66,73,82,85	16 (22%)
Americas (North America)	23,24,32,34,58,61,64,67,70,72	10 (14%)
Eastern Mediterranean	77	1 (1%)
Europe	28,30,38,43,46,47,48,52,53,54,55,59,68,69,81,83,84	17 (23%)
South-East Asia	50,51,63,80,86	5 (7%)
Western Pacific	15,16,31,36,39,40,63,75,76	9 (12%)
Global perspective	19,21,27,33,74,76,78	7 (10%)
Other*	17,29,49,71	4 (5%)
**Study design**		
Quantitative	15,16,29,30,31,35,36,37,40,41,44,45,48,49,50,51,59, 61,63,64,65,66,67,68,73,75,77,83,84,86	30 (41%)
Review	17,18,19,20,25,27,34,46,49,56,69,70,74,76,78,79	16 (22%)
Qualitative	14,22,23,38,43,55,54,62,71	9 (12%)
Descriptive	28,32,71,81,85	5 (7%)
Case study	26,58,72	3 (4%)
Mixed methods	39,42	2 (3%)
Other**	33,47,53,52	4 (5%)
**Target group**		
Contacts	14,15,21,22,24,27,32,35,36,37,42,44,74,75,76,81,82,84	18 (25%)
Migrants	20,28,30,34,38,43,46,47,48,49,54,55,56,59,61,64,68, 69,71,73,77,79,83	23 (32%)

For peer review only - http://bmjopen.bmj.com/site/about/guidelines.xhtml

Community	17,26,29,31,40,45,51,60,62,63	10 (14%)
High risk groups	19,25,33,57,66	5 (7%)
Homeless	23,53,58,67	4 (5%)
Asylum seekers or refugees	50,52	2 (3%)
People living with HIV	18,78	2 (3%)
Urban poor	16,39	2 (3%)
Other***	41,70,72,80,85,86	6 (8%)

944 **High HIV prevalence countries [17], high TB incidence countries [29], low-burden countries [49,71]*

945 ***Letter to editor [52], observational study [53], perspective [33], editorial [47]*

946 ****Population affected by TB outbreak [72], pastoralists [41], health care workers [65], prisoners [80], people living in slums [86], street connected youth and young adults [85], non-immigrant visitors [70]*

950 *Table 2 Barriers and facilitators for active case-finding policy implementation (n=43)*

**Reported factors**	**References**	**Number (%)**
Health system level		
Limited financial resources	16,21,22,26,27,32,39,40,43,46,48,51,54, 66,78	15 (35%)
Existing systems and structures	14,19,21,27,38,43,44,53,54,69,78	11 (26%)
Availability of diagnostic tests and services	16,19,27,34,41,49,50,66	8 (19%)
Staff experience, expertise, motivation	14,17,24,26,27,39,40,41,43,46,55,58,52,71	14 (33%)
Over-burdening staff	16,21,26,39,41,51,70	7 (16%)

For peer review only - http://bmjopen.bmj.com/site/about/guidelines.xhtml

Using person-centred approaches	14,22,23,27,34,43,53,65	8 (19%)
Collaboration and integration	14,16,18,39,43,34,52	7 (16%)
Health education	34,40,41,42,43	5 (12%)
Health information and research system level		
Data, systems, supervision	19,24,27,28,29,42,48	7 (16%)
Individual and community level		
Stigma and discrimination	14,22,23,24,25,27,28,31,37,38,43,50,58, 59,77,82	16 (37%)
Individual characteristics	23,31,34,37,39,43,51,58,65,66,77,83	12 (28%)
Socio-cultural factors	24,25,27,28,34,41,43,46,47,54,56,59,77,83	14 (33%)
Fear	23,24,34,38,39,43,53,55,54,56	10 (23%)
Mistrust	14,24,41,43,46,47,51,53,57,72	10 (23%)
Knowledge and awareness	14,23,24,26,37,43,53,58	8 (19%)

For peer review only - http://bmjopen.bmj.com/site/about/guidelines.xhtml

Fig 1. PRISMA flow diagram

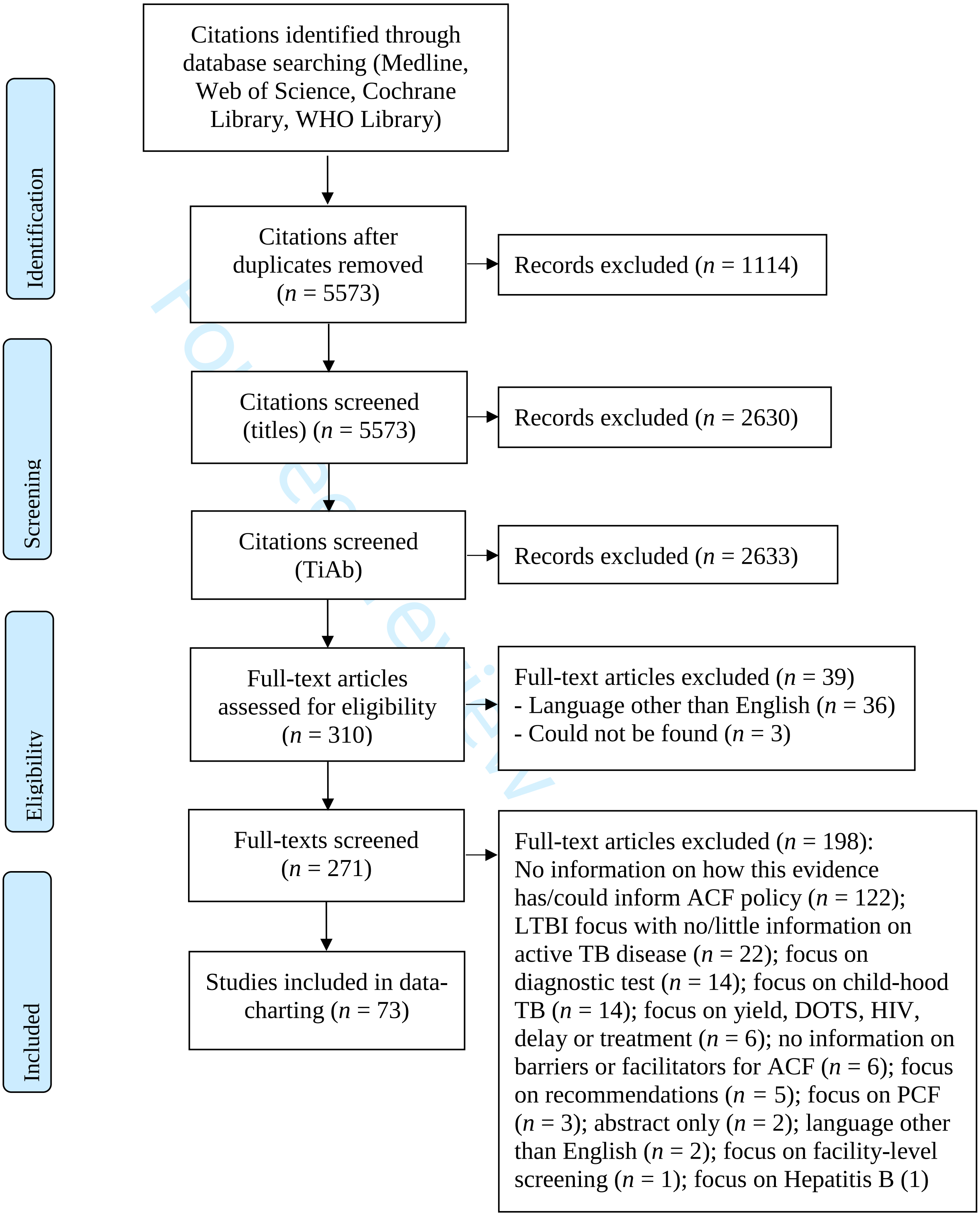

Abbreviations mentioned in Fig. 1: TiAb = Title and abstract; LTBI = Latent Tuberculosis Infection; DOTS = Directly Observed Treatment, Short Course; HIV = Human Immunodeficiency Virus; PCF = Passive Case-Finding

Preferred Reporting Items for Systematic reviews and Meta-Analyses extension for Scoping Reviews (PRISMA-ScR) Checklist

SECTION	ITEM	PRISMA-ScR CHECKLIST ITEM	REPORTED ON PAGE #
**TITLE**			
Title	1	Identify the report as a scoping review.	1
**ABSTRACT**			
Structured summary	2	Provide a structured summary that includes (as applicable): background, objectives, eligibility criteria, sources of evidence, charting methods, results, and conclusions that relate to the review questions and objectives.	2-3
**INTRODUCTION**			
Rationale	3	Describe the rationale for the review in the context of what is already known. Explain why the review questions/objectives lend themselves to a scoping review approach.	5-6
Objectives	4	Provide an explicit statement of the questions and objectives being addressed with reference to their key elements (e.g., population or participants, concepts, and context) or other relevant key elements used to conceptualize the review questions and/or objectives.	5-6
**METHODS**			
Protocol and registration	5	Indicate whether a review protocol exists; state if and where it can be accessed (e.g., a Web address); and if available, provide registration information, including the registration number.	No
Eligibility criteria	6	Specify characteristics of the sources of evidence used as eligibility criteria (e.g., years considered, language, and publication status), and provide a rationale.	7
Information sources*	7	Describe all information sources in the search (e.g., databases with dates of coverage and contact with authors to identify additional sources), as well as the date the most recent search was executed.	6
Search	8	Present the full electronic search strategy for at least 1 database, including any limits used, such that it could be repeated.	Additional file 2
Selection of sources of evidence†	9	State the process for selecting sources of evidence (i.e., screening and eligibility) included in the scoping review.	7-8
Data charting process‡	10	Describe the methods of charting data from the included sources of evidence (e.g., calibrated forms or forms that have been tested by the team before their use, and whether data charting was done independently or in duplicate) and any processes for obtaining and confirming data from investigators.	6
Data items	11	List and define all variables for which data were sought and any assumptions and simplifications made.	7-8
Critical appraisal of individual sources of evidence§	12	If done, provide a rationale for conducting a critical appraisal of included sources of evidence; describe the methods used and how this information was used in any data synthesis (if appropriate).	No

For peer review only

SECTION	ITEM	PRISMA-ScR CHECKLIST ITEM	REPORTED ON PAGE #
Synthesis of results	13	Describe the methods of handling and summarizing the data that were charted.	7-8
**RESULTS**			
Selection of sources of evidence	14	Give numbers of sources of evidence screened, assessed for eligibility, and included in the review, with reasons for exclusions at each stage, ideally using a flow diagram.	8
Characteristics of sources of evidence	15	For each source of evidence, present characteristics for which data were charted and provide the citations.	9
Critical appraisal within sources of evidence	16	If done, present data on critical appraisal of included sources of evidence (see item 12).	No
Results of individual sources of evidence	17	For each included source of evidence, present the relevant data that were charted that relate to the review questions and objectives.	Additional file 3
Synthesis of results	18	Summarize and/or present the charting results as they relate to the review questions and objectives.	10-16
**DISCUSSION**			
Summary of evidence	19	Summarize the main results (including an overview of concepts, themes, and types of evidence available), link to the review questions and objectives, and consider the relevance to key groups.	16-21
Limitations	20	Discuss the limitations of the scoping review process.	22-23
Conclusions	21	Provide a general interpretation of the results with respect to the review questions and objectives, as well as potential implications and/or next steps.	23-24
**FUNDING**			
Funding	22	a) Describe sources of funding for the included sources of evidence, as well as sources of funding for the scoping review. b) Describe the role of the funders of the scoping review.	a) No; b) 25

JBI = Joanna Briggs Institute; PRISMA-ScR = Preferred Reporting Items for Systematic reviews and Meta-Analyses extension for Scoping Reviews.
* Where *sources of evidence* (see second footnote) are compiled from, such as bibliographic databases, social media platforms, and Web sites.
† A more inclusive/heterogeneous term used to account for the different types of evidence or data sources (e.g., quantitative and/or qualitative research, expert opinion, and policy documents) that may be eligible in a scoping review as opposed to only studies. This is not to be confused with *information sources* (see first footnote).
‡ The frameworks by Arksey and O'Malley (6) and Levac and colleagues (7) and the JBI guidance (4, 5) refer to the process of data extraction in a scoping review as data charting.
§ The process of systematically examining research evidence to assess its validity, results, and relevance before using it to inform a decision. This term is used for items 12 and 19 instead of "risk of bias" (which is more applicable to systematic reviews of interventions) to include and acknowledge the various sources of evidence that may be used in a scoping review (e.g., quantitative and/or qualitative research, expert opinion, and policy document).

From: Tricco AC, Lillie E, Zarin W, O'Brien KK, Colquhoun H, Levac D, et al. PRISMA Extension for Scoping Reviews (PRISMA-ScR): Checklist and Explanation. Ann Intern Med. ;169:467–473. doi: 10.7326/M18-0850

For peer review only - http://bmjopen.bmj.com/site/about/guidelines.xhtml

Additional file 2: Literature search strategy for MEDLINE

We implemented the search strategy on 24 January 2018. The search yielded 4640 results.

1 exp Tuberculosis/ or exp Mycobacterium tuberculosis/

2 (tuberculosis or tb or pulmonary consumption or consumption, pulmonary or pulmonary phthisis or tuberculoses).ti,ab.

3 1 or 2

4 exp Mass screening/ or exp Mass chest x-ray/ or exp Contact tracing/ or exp Health surveys/ or exp Cross-sectional studies/ or exp Epidemiologic studies/ or exp Systematic review/

5 (Mass chest x ray$ or screen$ or mass screening$ or population screening$ or survey$ or screening survey$ or household survey or health survey$ or cross-sectional or detect$ or active case or case detect$ or case finding or tuberculosis case finding or active case finding or contact tracing or contact examination or contact screening or contact investigation or employment testing or intensified case finding or prevalence stud$ or prevalen* or inciden* or algorithm or undiagnosed or checking or pre-entry or passive or TB suspect$ or notification or notified).ti,ab.

6 4 or 5

7 (view$ or barrier$ or block$ or obstacle$ or hinder$ or constrain$ or facilitat$ or enabl$ or benefit$ or opportun$ or attitude$ or opinion$ or belief$ or perceiv$ or perception$ or aware$ or personal view$ or motivat$ or reason$ or incentiv$ or cost$ or cost-effective$ or cost analys$ or cost comparison or cost-minimization or cost measure$ or affordab$ or resource$ or resource allocation or resource$ or efficiency).ti,ab.

For peer review only

For peer review only - http://bmjopen.bmj.com/site/about/guidelines.xhtml

8 exp Attitude/ or exp Motivation/ or exp Cost/ or exp Cost analysis/ or exp Resource allocation/

9 7 or 8

10 exp Policy Making/ or exp Health Plan Implementation/ or exp Health Priorities/

11 (policy mak$ or policy develop$ or policy analys$ or health polic$ or policy implement$ or health plan implement$ or priorit$ or health priorit$).ti,ab.

12 10 or 11

13 9 or 12

14 (((((((((((((((((hard$ adj2 reach) or hard$) adj2 locate) or hard$) adj2 find) or hard$) adj2 treat) or difficult) adj2 locate) or Difficult) adj2 engage) or social$ exclu$ or social inequalit$ or difficult$) adj2 reach) or difficult$) adj2 find) or difficult$) adj2 treat).ti,ab.

15 ((geograph$ or transport$ or physical) and barrier$).ti,ab.

16 ((low$ or poor$ or negative) and (quality adj2 life)).ti,ab.

17 ((vulnerable or disadvantaged or at risk or high risk or low socioeconomic status or neglect$ or marginal$ or forgotten or non-associative or unengaged or hidden or excluded or transient or inaccessible or underserved or stigma$ or inequitable) and (people or population$ or communit$ or neighbourhood$1 or neighborhood$1 or group$ or area$1 or demograph$ or patient$ or social$)).ti,ab.

18 vulnerable populations/

19 17 or 18

For peer review only - http://bmjopen.bmj.com/site/about/guidelines.xhtml

20 poverty area/

21 (refuser$1 or non-user$1 or discriminat$ or shame or prejud$ or racism or racial discriminat$).ti,ab.

22 social support/ or *social conditions/ or stigma/ or Social Isolation/ or *quality of life/ or Prejudice/ or Socioeconomic Factors/

23 (immobile or (disabled and (house bound or home bound)) or ((house or home) and bound)).ti,ab.

24 Homebound Persons/

25 23 or 24

26 ((hous$ and (quality or damp$ or standard$ or afford$ or condition$ or dilapidat$)) or ((emergency or temporary or inadequate or poor$ or overcrowd$ or over-crowd$ or over-subscribed) and (hous$ or accommodation or shelter$ or hostel$ or dwelling$))).ti,ab.

27 housing/st

28 26 or 27

29 (rough sleep$ or runaway$1 or ((homeless$ or street or Destitut$) and (population or person$1 or people or group$ or individual$1 or shelter$ or hostel$ or accommodation$1))).ti,ab.

30 exp homeless persons/

31 29 or 30

32 ((drug$ or substance) and (illegal or misus$ or abuse or intravenous or IV or problem use$ or illicit use$ or addict$ or dependen$ or dependant or delinquency)).ti,ab.

For peer review only

33 *Substance-Related Disorders/ or Drug users/

34 32 or 33

35 ((alcohol$ and (misus$ or abuse or problem$ use$ or problem drink$ or illicit use$ or addict$ or dependen$ or dependant or delinquency)) or alcoholic$1).ti,ab.

36 *Alcohol- Related Disorders/ or Alcoholics/

37 35 or 36

38 (prostitution or sex work$ or transactional sex$ or prostitute$1).ti,ab.

39 Prostitution/

40 38 or 39

41 (poverty or deprivation or financial hardship$).ti,ab.

42 ((low-income or low income or low pay or low paid or poor or deprived or debt$ or arrear$) and (people or person$1 or population$1 or communit$ or group$ or social group$ or neighbourhood$1 or neighborhood$1 or famil$)).ti,ab.

43 poverty/

44 (low$ and social class$).ti,ab.

45 (traveller$1 or gypsies or gypsy or Romany or roma).ti,ab.

46 gypsies/

47 45 or 46

48 (mental$ and (health or ill or illness)).ti,ab.

For peer review only

For peer review only - http://bmjopen.bmj.com/site/about/guidelines.xhtml

49 *mental health/ or Mentally Ill Persons/

50 48 or 49

51 ((((health care worker$1 or health care) adj2 service provi$) or health-care) adj2 provi$).ti,ab.

52 (outreach adj2 worker$1).ti,ab.

53 Community health aides/

54 52 or 53

55 (support adj2 worker$1).ti,ab.

56 (case adj2 worker$1).ti,ab.

57 (social adj2 worker$1).ti,ab.

58 social care professional$1.ti,ab.

59 ((social care adj2 service provi$) or (social-care adj2 provi$)).ti,ab.

60 (((language$ or communicat$) and (barrier$ or understand$ or strateg$ or proficien$)) or translat$ or interpret$ or (cultur$ and competen$)).ti,ab.

61 Communication Barriers/ or *Language/

62 60 or 61

63 (((immigrant$ or migrant$ or asylum or refugee$ or undocumented or foreign born or born) adj overseas) or (displaced and (people or person$1))).ti,ab.

For peer review only

64 ((((Emigration.mp. and Immigration/) or refugees/ or Transients.mp.) and migrants/) or Emigrants.mp.) and immigrants/ [mp=title, abstract, original title, name of substance word, subject heading word, keyword heading word, protocol supplementary concept word, rare disease supplementary concept word, unique identifier, synonyms]

65 63 or 64

66 exp Diabetes Mellitus/

67 diabet$.tw,ot.

68 (IDDM or NIDDM or MODY or T1DM or T2DM or T1D or T2D).tw,ot.

69 (non insulin$ depend$ or noninsulin$ depend$ or non insulin?depend$ or noninsulin?depend$).tw,ot.

70 (insulin$ depend$ or insulin?depend$).tw,ot.

71 or/66-70

72 14 or 15 or 16 or 19 or 20 or 21 or 22 or 25 or 28 or 31 or 34 or 37 or 40 or 41 or 42 or 43 or 44 or 47 or 50 or 51 or 54 or 55 or 56 or 57 or 58 or 59 or 62 or 65 or 71

73 3 and 6 and 13 and 72

74 limit 73 to yr="1968 -Current"

75 animals/ not humans/

76 74 not 75

For peer review only

Additional file 3: Abridged version of the data charting table

The abridged data charting table contains information on author, year of publication and study design (column 1), the country where the study was implemented and the target population (column 2), ACF antecedents (column 3), ACF components (column 4), factors influencing ACF policy implementation at the level of the health system (column 5) and the individual and community level (column 6).

	1	2	3	4	5	6
**ID**	**Author, year, design**	**Country, target population**	**ACF antecedents**	**ACF components**	**Factors influencing ACF policy implementation:** health system context	**Factors influencing ACF policy implementation:** individual and community context
1	Abubakar, 2012, review	Low-burden, migrants			Screening site characteristics; diagnostic test availability; molecular assay costs	
2	Adejumo, 2016, qualitative	Nigeria, community			CHW age, work experience, knowledge	
3	Akkerman, 2016, letter to editor	the Netherlands, asylum-seekers			High-volume CXR readers; cooperation between public health and clinical workers	

For peer review only - http://bmjopen.bmj.com/site/about/guidelines.xhtml

4	Alvarez*, 2011, descriptive	Low-burden, migrants	despite lack of evidence, ACF is major global health priority			
5	Ayakaka, 2017, qualitative	Uganda, contacts		Education and incentivization for health workers; persuasion; enablement	CHW travel costs, pay, skills; personalized, enabling services; staff shortages; fear of TB; patient communication; language barrier; difficulty locating households; lack of ACF guidelines	Fear of stigma; discrimination; among contacts: limited knowledge, mistrust, travel costs, avoidant behavior; privacy provided by home evaluation
6	Ayles, 2013, quantitative	South Africa, Zambia, contact			Linkage and retention in care	Empowerment to seek care early
7	Azman*, 2014, quantitative	China, India, South Africa, community				
8	Baxter, 2017, review	Worldwide, contacts			Using molecular epidemiological testing; ensuring adequate systems; local expertise and capability; communication; coordination; prompt action; addressing fear; error; lack of resources	Stigma; language barrier; high mobility

For peer review only - http://bmjopen.bmj.com/site/about/guidelines.xhtml

For peer review only

9	Bell*, 2013, quantitative	USA, migrants				
10	Bloss, 2011, case study	USA, outbreak			Institutional knowledge about TB control; limited human resources	Community trust and rapport
11	Van den Bosch, 2000, descriptive	UK, migrants		Incentivization for screening participants; health education for general public		Change of address; false address; language barrier; mistrust of authorities; fear of TB and stigma
12	Brewin, 2006, qualitative	UK, migrants			Integration of health and social services; waiting time	Feelings of reassurance, responsibility, anxiety linked to ACF; lack of public health information
13	Chemtob, 2003, qualitative	Israel, migrants			Adequate health facilities; funds for transportation; attitudes; dialogue with immigrants and providers in planning and implementation	Socio-cultural barriers; anti-immigrant sentiments; fear of deportation; no standard term for TB; empowerment to assume greater responsibility for health promotion

For peer review only - http://bmjopen.bmj.com/site/about/guidelines.xhtml

14	Cook, 2012, descriptive	Canada, aboriginal community			Foundation of routine ACF including interview skills, dedicated resources to undertake ACF, follow-up	Community buy-in
15	Corbett, 2013, review	Africa, PLHIV	Radiological screening widely implemented before 1970s	Target group: populations with high prevalence	Sensitivity of diagnostic tests; communication and interaction with the community; transportation	
16	Corbett, 2010, quantitative	Zimbabwe, congregate setting	ACF implemented in HIC since 1920s; ACF discouraged due to costs and weak treatment programmes			
17	Datiko, 2017, quantitative	Ethiopia, community		Linkage to laboratory services and treatment through a transport network		
18	de Vries, 2017, review	Worldwide, high-risk		Awareness; cultural sensitivity; access to high-quality	Structural barriers; cultural and language barriers	Low perceived susceptibility; stigma and its social consequences

For peer review only - http://bmjopen.bmj.com/site/about/guidelines.xhtml

				translation services; cross-cultural training of providers		
19	Dierberg, 2016, quantitative	India, refugees			Use of Xpert MTB/RIF; available time and human resources; lack of a laboratory technician; trained providers	High mobility; awareness; stigma
20	Eang, 2012, quantitative	Cambodia, contacts	Growing interest in ACF in LMIC			
21	Erkens*, 2008, quantitative	the Netherlands, migrants				
22	Mhimbira, 2017, review	Worldwide, contacts		Health promotion activities; education for health workers		
23	Fox, 2018, quantitative	Vietnam, contacts	ACF in high-prevalence settings remains limited			

For peer review only - http://bmjopen.bmj.com/site/about/guidelines.xhtml

24	Fox, 2013, review	Worldwide, contacts, congregate setting	Growing interest ACF in LMICs			
25	Fox, 2015, quantitative	Vietnam, contacts	WHO recommendations for ACF		Proximity of services	Discrimination; lack of knowledge; misconceptions; attitudes and (traditional) beliefs; difficulty remembering appointments
26	Getnet, 2017, quantitative	Ethiopia, pastoralists			Proximity of services; diagnostic algorithm and tools used; quality of diagnostic services; provider expertise; burden on CHWs; health education for TB patients	Health seeking behavior; lack of confidence in CHWs; personal or cultural beliefs; gender norms
27	Godfrey-Faussett, 2003, review	Worldwide, high HIV prevalence setting	Movement towards decentralized diagnostic centers		Fear; stigma	
28	Golub, 2005, review	Worldwide, contacts	Mass radiography as ACF strategy in HICs before 1960s; WHO		Workload and cost; method of case detection; training of laboratory techni-	

For peer review only - http://bmjopen.bmj.com/site/about/guidelines.xhtml

For peer review only

			recommendation against indiscriminate mass screening (1974); exploration of new ACF strategies; recognized global urgency		cians, clinicians and field staff; appropriate bacteriological examination; laboratory network	
29	O'Hara, 2015, quantitative	South Africa, health care workers			Cost and confidentiality of services; waiting times	Sex, occupation, time since a previous TB test
30	Harper, 1996, quantitative	Nepal, remote community			Cost; lack of staff and funding; absenteeism; use of 'alternative' services; accessibility of services	Low confidence amongst users and general under-usage of services
31	Hinderaker, 2011, quantitative	Low-income, low-income community	Testing of different ACF approaches since 1960s, WHO targets set 2005	Social mobilization; health education for general public; communication; private sector engagement;	Regular supervision; availability of district laboratory supervisor	

For peer review only - http://bmjopen.bmj.com/site/about/guidelines.xhtml

				health systems strengthening; in-centivization for health workers and TB patients		
32	Hogan, 2005, quantitative	UK, migrants			Service capacity; lack of national guid-ance; inconsistency in implementing ACF	
33	Honarvar, 2014, quantitative	Iran, migrants				Stigma; high mobility; poverty; low literacy; tribal behaviors; traditional beliefs; gender norms; language bar-rier; self-discrimination
34	Karki, 2017, quantitative	Papua New Guinea, commu-nity	Role of ACF uncertain since 1970s; WHO rec-ommendation against indiscriminate mass screening (1974)		Poor health services; lack of outreach services; limited funds at district level; cost; availability of staff and the pay-ment of allowances; staff skills and ex-perience; health education for general public	

For peer review only - http://bmjopen.bmj.com/site/about/guidelines.xhtml

35	Kerrigan, 2017, qualitative	South Africa, contacts	Despite lack of evidence, ACF is major global health priority		Provider training, safety; incentivization for screening participants; lack of resources, logistics; involvement of culturally respected, valued stakeholder/organization venues; community-based mobilization and events	Stigma; preference of different screening locations; travel time and costs; gossip
36	Khan*, 2015, quantitative	Canada, migrants				
37	Klinkenberg, 2009, review	EU, migrants			Access to healthcare for migrants; availability of a good follow-up system; integration with healthcare	
38	Kranzer, 2010, review	Worldwide, PLHIV	Mass radiography in Czechoslovakia 1961-72 showing decrease in prevalence	Target population; screening strategy; laboratory capacity; operational feasibility; costs	Feasibility and costs; laboratory capacity	

For peer review only

39	Kranzer, 2012, review	South Africa, deprived community		Incentivization for screening participants		Waiting time; ability to produce a sputum sample; health status
40	Kulane, 2010, qualitative	Sweden, Somali community				Stigma; migration; migration policies; fear of deportation; misconceptions
41	Layton, 1995, review	USA, homeless			Incentivization for screening participants	
42	Li, 2017, case study	China, community			Heavy workload; inadequate providers; time and human resource constraints; provider education and competence	Acceptance; knowledge; awareness; health status; incentivization for screening participants; health education for TB patients
43	Lorent, 2015, mixed methods	Cambodia, urban poor		Health education for general public; community involvement; peer support networks	Challenging working conditions; collaboration between TB workers and CHWs; CHW compensation, familiarity, knowledge of community, remuneration; provider motivation; financial support	Acceptance; cost, fear; embarrassment; preference of different screening locations; privacy concerns; involvement of CHWs; non-acceptance of diagnosis

For peer review only - http://bmjopen.bmj.com/site/about/guidelines.xhtml

44	Lorent, 2014, quantitative	Cambodia, urban poor		Flexible patient-centered treatment approach	Logistics; finances; heavy workload; CHW integration, network, recognition, remuneration; use of mobile phone, collaboration with providers and laboratory technicians; infrastructure	
45	Lönnroth, 2013, review	Worldwide, high-risk	WHO recommendation against indiscriminate mass screening (1974)	Confidentiality; access to free, good quality services; provider training; minimization of initial loss to follow-up; enforced notification; engagement of public/private providers; resources	Available, high-quality services; NTP performance; health system capacity; public health law; existing platforms for outreach and health promotion activities, health and social services	.;

For peer review only - http://bmjopen.bmj.com/site/about/guidelines.xhtml

46	Lönnroth, 2017, review	Low incidence, migrants		Target groups; screening algo-rithms; timing; val-ues; preferences; competing health care needs; weak evidence base		
47	Lönnroth, 2015, descriptive	Low-incidence, high-risk				Trust
48	MacPherson, 2006, review	Low-incidence, migrants				Fear; language barrier; culture; social factors
49	Mallick*, 2017, descriptive	India, prisoners				
50	Mayo, 1996, case study	USA, homeless			ACF location; incentivization for TB pa-tients; providers' frustration; involve-ment of clients	Stigma; substance use; poor social skills; health status; poor access to services and the necessities of life; unemployment; homelessness

For peer review only - http://bmjopen.bmj.com/site/about/guidelines.xhtml

For peer review only

51	Mor, 2008, quantitative	Israel, migrants		Unification of medical procedures	Operational cost; infrastructure	
52	Morishita, 2016, quantitative	Cambodia, contacts				Cost; insurance
53	Mulder, 2011, descriptive	the Netherlands, contacts			Limited health system capacity to keep track of immigrant index cases	
54	Mwansa-Kambafwile, 2013, quantitative	South Africa, contacts				Discomfort linked to home visits; confidentiality; stigma; discrimination
55	Ospina, 2012, quantitative	Spain, migrants		Involvement of CHWs	CHW involvement; language barrier	Birthplace; unknown district of residence; HIV infection; homelessness; sex; incarceration history; language and cultural barrier
56	Seedat, 2014, qualitative	UK, migrants		Community, proactive services; collaboration with	Lack of capacity, funding, advocacy, psycho-social support services, aware-	Stigma; culture; gender norms; faith; language barrier; misconceptions; fear of lack of confidentiality, cost,

For peer review only - http://bmjopen.bmj.com/site/about/guidelines.xhtml

For peer review only

				community organizations; combining diseases into health check-up; awareness raising	ness; link with community organizations; cultural insensitivity; time and distance to services	disease status; lack of awareness, knowledge, confidence
57	Sekandi, 2015, quantitative	Uganda, contacts	ACF currently used in research settings		Lack of well-organized public health systems; staff shortage; limited resources for personnel time, administration and transportation	
58	Shrestha-Kuwahara, 2003, qualitative	USA, contacts			Lack of clear definitions; challenge of communicating the need to obtain personal information; limited knowledge on socio-economic issues among staff; training of, frustration among providers	Trust; understanding; knowledge; feeling moral obligations to family/friends confusion; fear of alienation, abandonment, stigma, loss of employment or housing; negative perception of interview process; misconceptions; language and cultural barriers

For peer review only - http://bmjopen.bmj.com/site/about/guidelines.xhtml

For peer review only

59	Shrivastava, 2013, quantitative	India, slums	Periodic ACF remains an integral part of TB control in high-risk groups	Involvement of CHW		
60	Smit*, 2017, quantitative	Belgium, high-risk, contacts				
61	Southern, 1999, observational	UK, homeless		Immediate examination of CXR; fast-track referral system	Non-judgmental, flexible, supportive approach	Lack of interest; fear of the outcome; waiting times; health education of screening participants
62	Suthar, 2016, perspective	Worldwide, high-risk	2030 goals important for inspiring and persuading communities to collaborate and step up TB response			
63	Swigart, 2004, qualitative	USA, homeless				Health status; knowledge about TB; costs; fear; disinterest

For peer review only - http://bmjopen.bmj.com/site/about/guidelines.xhtml

64	Szkwarko, 2016, descriptive	Kenya, street-connected youth and young adults			Trust; difficulties obtaining morning sputum; underrepresentation of women	
65	Tan, 2001, review	USA, nonimmigrant visitors			Funding and logistical difficulties; technical issues; overburdening providers	
66	Tankimovich, 2013, review	USA, homeless, migrants		Coordination and collaboration of programmes	Education and incentivization for screening participants and TB patients; accessible, affordable facilities and technology; cultural knowledge; coordination between governments, academic and private institutions	Lack of knowledge; misconceptions; contact difficulties; co-morbidities; cultural values
67	Tardin, 2009, quantitative	Switzerland, migrants				Stigma; different communication styles and health beliefs; language barrier; acceptance of diagnosis; respect towards authority; religious value; tight community network

For peer review only - http://bmjopen.bmj.com/site/about/guidelines.xhtml

68	Tupasi, 2000, quantitative	Philippines, community			Services free and conveniently located	Symptoms; fear of stigma
69	Welshman, 2006, review	UK, migrants			Dissatisfaction with work; disjunction between guidelines and practice; cost; logistics; administration; language barrier; shortage of radiologists/ radiographers	Changes of address; language barrier; mistrust of authorities
70	Welshman, 2006, editorial	UK, migrants			Logistics; administration; language barrier; shortages of radiologists/radiographers	Changes of address; language barrier; mistrust of authorities
71	Wright, 1979, quantitative	South Africa, high-risk			Limited and inadequate facilities for the examination of sputum; cost	"Degree of sophistication of the people involved"
72	Yassin, 2013, mixed methods	Ethiopia, contacts		Capacity strengthening; advocacy; communication; social mobilization; transport; treat-	Providers' commitment; CHWs' job satisfaction; laboratory technicians' perceived benefit of ACF; burden on laboratory; incentivization of laboratory technicians; benefit from new equipment; quality of smears prepared by CHWs	

For peer review only - http://bmjopen.bmj.com/site/about/guidelines.xhtml

				ment; CHW training, supervision, support		
73	Zenner, 2017, review	Worldwide, migrants	WHO End TB strategy; WHO against indiscriminate mass screening (1974)		Type of programme; target group; voluntary vs mandatory ACF	

* The article contains important information on additional influencing factors beyond the ones displayed in this table. Due to space limitations, we did not include them here.

For peer review only

For peer review only - http://bmjopen.bmj.com/site/about/guidelines.xhtml
